# Piezoelectric Materials for Energy Harvesting and Sensing Applications: Roadmap for Future Smart Materials

**DOI:** 10.1002/advs.202100864

**Published:** 2021-07-13

**Authors:** Susmriti Das Mahapatra, Preetam Chandan Mohapatra, Adrianus Indrat Aria, Graham Christie, Yogendra Kumar Mishra, Stephan Hofmann, Vijay Kumar Thakur

**Affiliations:** ^1^ Technology & Manufacturing Group Intel Corporation 5000 West Chandler Boulevard Chandler Arizona 85226 USA; ^2^ Surface Engineering and Precision Centre School of Aerospace Transport and Manufacturing Cranfield University Cranfield MK43 0AL UK; ^3^ Institute of Biotechnology Department of Chemical Engineering and Biotechnology University of Cambridge Cambridge CB2 1QT UK; ^4^ Mads Clausen Institute NanoSYD University of Southern Denmark Alsion 2 Sønderborg 6400 Denmark; ^5^ Division of Electrical Engineering Department of Engineering University of Cambridge Cambridge CB2 1PZ UK; ^6^ Biorefining and Advanced Materials Research Center Scotland's Rural College (SRUC) Kings Buildings Edinburgh EH9 3JG UK; ^7^ Department of Mechanical Engineering School of Engineering Shiv Nadar University Delhi Uttar Pradesh 201314 India

**Keywords:** energy harvesting, flexible devices, nanostructured materials, piezoelectric nanogenerator, polymer nanocomposites, polyvinylidene fluoride copolymers

## Abstract

Piezoelectric materials are widely referred to as “smart” materials because they can transduce mechanical pressure acting on them to electrical signals and vice versa. They are extensively utilized in harvesting mechanical energy from vibrations, human motion, mechanical loads, etc., and converting them into electrical energy for low power devices. Piezoelectric transduction offers high scalability, simple device designs, and high‐power densities compared to electro‐magnetic/static and triboelectric transducers. This review aims to give a holistic overview of recent developments in piezoelectric nanostructured materials, polymers, polymer nanocomposites, and piezoelectric films for implementation in energy harvesting. The progress in fabrication techniques, morphology, piezoelectric properties, energy harvesting performance, and underpinning fundamental mechanisms for each class of materials, including polymer nanocomposites using conducting, non‐conducting, and hybrid fillers are discussed. The emergent application horizon of piezoelectric energy harvesters particularly for wireless devices and self‐powered sensors is highlighted, and the current challenges and future prospects are critically discussed.

## Graphical abstract

This paper presents a comprehensive review of the energy harvesting performance of different types of piezoelectric materials. These materials include nanostructured materials, polymers, polymer nanocomposites synthesized using different types of fillers and piezoelectric films. The fabrication techniques, energy harvesting mechanisms, and applications of piezoelectric nanogenerators built using these materials are discussed thoroughly.

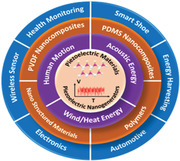

## 1 Introduction

Anthropogenic environmental pollution and climate change‐related to energy production and consumption present a key challenge to humanity and our technological future. The development of renewable, environmentally friendly, and cost‐effective energy sources has increasingly become important to meet the energy demands of the future.^[^ ^1^ ^]^ There are various renewable energy types present in our environment ranging from kinetic energy to solar energy and bioenergy, and extensive research has been conducted on energy harvesting technologies to scavenge energy from readily available sources like vibration, human motion, water, air, heat, light, chemical reactions into useful electrical energy for powering small scale devices.^[^ ^2^, ^3^, ^4^, ^5^ ^]^ While there remain many challenges for such energy harvesting methods including variable and unpredictable ambient conditions, excellent progress in wireless communication, and low power integrated circuits (ICs) have decreased power consumption requirements and further attracted portable energy harvesting approaches.^[^ ^2^ ^]^ For example, the power requirement of the latest generation of personal wireless communication devices is of the order of tens of mW.^[^ ^6^, ^7^ ^]^ Advanced wireless technologies and microelectronics have facilitated the development of a new generation of wearable devices such as, body‐mounted sensors, fitness trackers, smart clothing, augmented reality sensors, etc. In addition, the concept of the Internet of Things (IoT) has led to placing smart equipment in remote areas such as, health care devices inside the human body, where it is difficult and sometimes impossible to charge batteries. Therefore, energy harvesting has become necessary to sustain such self‐powered systems.

Among ambient energy sources, low‐intensity kinetic energy from random displacements, human activities such as, finger tapping, running, walking, heartbeat, respiration; structural vibrations from manufacturing equipment and transportation vehicles; low Reynolds‐number flow of fluids like wind, water are abundantly available. Recent research has focused on kinetic energy harvesting based on three main transduction mechanisms: Electromagnetic, piezoelectric, and triboelectric. The advantages and disadvantages of these energy harvesting methods have been widely discussed in the literature,^[^ ^8^ ^]^ with most studies focusing on piezoelectric transduction motivated by its superior power densities, high energy conversion efficiency, simpler architectures, and high scalability.^[^ ^9^, ^10^ ^]^ As a result, applications of piezoelectric materials have increased tremendously across numerous fields including sensors,^[^ ^11^, ^12^, ^13^, ^14^, ^15^, ^16^, ^17^, ^18^ ^]^ actuators,^[^ ^19^, ^20^, ^21^ ^]^ nanogenerators,^[^ ^22^, ^23^, ^24^, ^25^, ^26^, ^27^, ^28^, ^29^, ^30^ ^]^ MEMS devices,^[^ ^31^, ^32^, ^33^ ^]^ portable electronics,^[^ ^34^, ^35^ ^]^ and biomedicine.^[^ ^36^, ^37^, ^38^, ^39^, ^40^ ^]^

Currently, the most widely used classes of piezoelectric materials range from lithium niobate (LiNbO_3_),^[^ ^41^ ^]^ lead magnesium niobate‐lead titanate (PMN‐PT),^[^ ^42^ ^]^ zinc oxide (ZnO),^[^ ^43^ ^]^ lead zirconate titanate (PZT),^[^ ^44^, ^45^, ^46^ ^]^ barium titanate (BaTiO_3_),^[^ ^47^ ^]^ to polyvinylidene fluoride (PVDF), and its copolymers.^[^ ^25^, ^48^, ^49^ ^]^ Although lead (Pb) based ceramics such as, PZT exhibit high piezoelectric coefficients, their rigidity, brittleness, and toxicity limit their applications in flexible and stretchable devices.^[^ ^13^ ^]^ Due to Pb toxicity, there is increasing concern about the use of PZT based devices in consumer products such as various smart systems, medical imaging devices, automobiles, and sound generators.^[^ ^13^ ^]^ PZT based ceramics are also unsuitable for high‐temperature applications. On the contrary, Pb free ceramics such as, KNbO_3_, NaNbO_3_, etc., are biocompatible which allows their versatile utilization in sensors and actuators transplanted directly into living bodies. Additionally, their properties can be easily tailored which make them the best alternative in contrast to Pb based PZT ceramics. Polymers possess high flexibility, excellent stability, and biocompatibility, which makes them favorable for integration into flexible devices. The peak power density values of piezoelectric energy harvesters (PEHs) made using zinc oxide (ZnO) nanowires is up to 11 mW cm^–3^,^[^ ^50^ ^]^ PZT nanowires up to 2.8 mW cm^–3^,^[^ ^51^ ^]^ BaTiO_3_/P(VDF‐HFP) nanocomposites up to 0.48 Wcm^–3^.^[^ ^52^ ^]^ For the complete list of power density values, readers can refer to **Tables** **1**–**3**.

**Table 1:** Summary of piezoelectric nanostructured materials for energy harvesting applications

Nanostructured material	Length [*L*]/Diameter [*D*]/Thickness [*T*] of nanostructure	Synthesis method	Piezoelectric strain coefficient d_33_ [pC N^–1^]	Poling conditions	Applied load/pressure/strain%/load resistance	Output voltage [V]	Output current/current density	Power/power density	References
1D ZnO nanostructures									
ZnO nanowires	*L* = 0.2–0.5 µm	Vapor liquid‐solid process	–	No poling	5 nN	(6–9) 10^–3^	–	≈10 pW µm^–2^	[73, 74]
Phosphorus doped‐ ZnO nanowires	*D* = 50 nm *L* = 600 nm	Thermal vapor deposition	–	No poling	90–120 nN	0.05–0.09	–	–	[80]
ZnO nanowires	*D* = 100–800 nm *L* = 100–500 µm	Thermal vapor deposition	–	No poling	From finger tapping and running hamster	0.1–0.15	≈0.0005	–	[81]
ZnO nanorods	*D* = 100 nm *L* = 1.5–2 mm	Aqueous solution using Zn(NO_3_)_2_.6H_2_O and HMTA	–	No poling	0.9 kgf	–	≈1 µA cm^–2^	–	[84]
ZnO nanorods	*D* = 100 nm *L* = 1.5–2 mm	Aqueous solution using Zn(NO_3_)_2_.6H_2_O and HMTA	–	No poling	0.9 kgf	–	4.76 µA cm^–2^	–	[85]
ZnO nanorods	*L* ≈ 2 µm *D* < 100 nm	Aqueous solution using Zn(NO_3_)_2_.6H_2_O and HMTA	–	No poling	Bending and rolling	–	2 µA cm^–2^	–	[86]
ZnO nanowires	*D* = 150 nm *L* = 2 µm	Aqueous solution using Zn(NO_3_)_2_.6H_2_O and HMTA	–	No poling	0.12% strain	10	0.6 µA	10 mW cm^–3^	[87]
ZnO nanowires	–	Hydrothermal process	–	No poling	–	20	6 µA	0.2 W cm^–3^	[88]
ZnO nanowires	–	Hydrothermal process	–	No poling	Punching by human palm	58	134 µA	0.78 W cm^–3^	[89]
ZnO nanowires	*L* = 30 µm	Vapor deposition	–	No poling	0.11% strain	2	50 nA	–	[90]
ZnO nanorods	Aspect ratio = 20:1	Aqueous solution using Zn(NO_3_)_2_.6H_2_O and HMTA	–	No poling	0.9 mN	4 × 10^–5^	4 nA	0.76 µW cm^–2^	[91]
ZnO nanorods	*L* = 2 µm *D* = 50–60 nm	Aqueous solution using Zn(NO_3_)_2_.6H_2_O and HMTA	–	No poling	50 g acceleration	1.07	1.88 mA cm^–2^	434 µW cm^–2^	[93]
ZnO nanowires	*D* = 300 nm *L* = 4 µm	Wet chemical method	–	No poling	0.19% strain	1.2	26 nA	≈70 nW cm^–2^	[94]
ZnO nanowires	*D* = 200 nm *L* = 50 µm	Physical vapor deposition	–	No poling	0.1% strain	2.03	107 nA	≈11 mW cm^–3^	[50]
ZnO nanowires & Au‐coated ZnO NWs	*D* = 50–200 nm *L* = 3.5 µm	Hydrothermal process	–	No poling	–	0.001	5 pA	–	[95]
ZnO nanowires & Pd‐coated ZnO NWs	–	Hydrothermal process	–	No poling	–	0.003	17 pA	–	[96]
ZnO nanorods	–	Hydrothermal process	–	No poling	–	0.01	10 nA	–	[97]
ZnO nanowires	*D* = 100 nm *L* = 1 µm	Hydrothermal process	–	No poling	Sound waves at 100 dB, 100 Hz	8	2.5 µA	–	[98]
ZnO nanorods	*D* ≈ 150 nm *L* ≈ 1.5 µm	Template‐free electrochemical deposition	11.8	No poling	80 nN	1.5	0.4 µA	–	[99]
2D ZnO nanostructures									
ZnO nanosheets	width ≈ 80 nm *L* ≈ 3 µm	Aqueous solution using Zn(NO_3_)_2_.6H_2_O and HMTA	–	No poling	4 kgf	≈0.7	≈17 µA cm^–2^	≈11.8 µW cm^–2^	[106]
Vanadium doped ZnO nanosheets	Width = 900 nm–1.0 µm *T* = 15–20 nm	Aqueous solution using Zn(NO_3_)_2_.6H_2_O, HMTA and V_2_O_5_	4	No poling	0.5 kgf	–	1.0 µA cm^–2^	–	[107]
ZnO nanowall and nanowall‐nanowire hybrid	*T* = 200 nm, *L* = 2.4 µm *T* = 90 nm, *L* = 200 nm	Chemical vapor deposition	–	No poling	0.5 kgf	0.002	≈500 nA cm^–2^	–	[108]
ZnO nanowalls	*T* = 60–80 nm *L* = 2–3 µm	Hydrothermal process	–	No poling	Folding by human finger	2.5	80 nA	0.2 µW cm^–2^	[109]
ZnO nanorods and nanowalls	*D* = 42 ± 5 nm *T* ≈ 38 ± 28 nm, *L* ≈ 950 ± 370 nm	Chemical bath deposition	7.01 2.63	No poling	–	–	–	–	[110]
Nanostructures of other piezoelectric materials									
PZT nanofibers	*D* ≈ 60 nm *L* ≈ 500 µm	Electrospinning	500‐600	4 V µm^–1^ above 140 °C for 24 h	6 MΩ load resistance	1.63	–	0.03 µW	[118]
PZT nanofibers	*D* = 370 nm	Electrospinning	–	4 kV mm^–1^ at 130 °C for 15 min	100 MΩ load resistance	6	45 nA	200 µW cm^–3^	[119]
PZT nanowires	–	Electrospinning	–	4 V µm^–1^ at 130 °C for 10 min	100 MΩ load resistance	3.2	50 nA	170 µW cm^–3^	[115]
PZT nanowires	*L* = 420 µm	Electrospinning	–	5 kV mm^–1^ at 130 °C for 15 min	0.53 MPa	209	23.5 µAcm^–2^	–	[120]
PZT nanowires	*D* = 500 nm *L* = 5 µm	Chemical epitaxial growth on Nb‐doped SrTiO_3_ substrate	152	100 kV cm^–1^ in a dielectric fluid	Bending strain	0.7	4 µAcm^–2^	2.8 mW cm^–3^	[51]
ZnO nanowires/PZT heterojunction	*L* ≈ 3 µm *D* = 50 nm	Hydrothermal process and magnetron sputtering	–	Corona poling at 11 kV for 30 min	0.9 kgf	–	270 nA	–	[122]
PMN‐PT nanowires	*D* = 400nm *L* = 200–800 nm	Hydrothermal process	381	No poling	–	–	–	–	[123]
Mn doped (Na_0.5_K_0.5_)NbO_3_ (NKN) nanofibers	*D* ≈ 130 nm	Electrospinning under 13 kV	40.06	60 kV cm^–1^ at 100 °C for 30 min	Bending strain	0.3	50 nA	–	[124]
BaTiO_3_ nanowires	*D* ≈ 90 nm *L* ≈ 1 µm	Two‐step hydrothermal process	–	120 kV cm^–1^ at RT for 24 h	1 g RMS acceleration	0.085	≈0.316 nA	6.27 µW cm^–3^	[126]
BaTiO_3_ nanowires	*D* ≈ 600–630 nm *L* ≈ 45 µm	Two‐step hydrothermal process	–	≈75 kV cm^–1^ at RT for 12 h	0.25 g RMS acceleration	0.34	–	–	[127]
0.93(Na_0.5_Bi_0.5_)TiO_3_‐0.07BaTiO_3_ nanofibers	–	Sol–gel electrospinning	109	–	Human finger	30	80 nA	–	[128]
GaN nanowires	*L* = 121 ± 37 nm *D* = 88 ± 21 nm	Plasma‐assisted molecular beam epitaxy	–	No poling	173 nN	0.44	–	–	[130]
GaN nanowires	*D* = 45 ± 20 nm *L* = 1 µm ± 120 nm	Plasma‐assisted molecular beam epitaxy	–	No poling	1.5 N	0.35	–	≈12.7 mW cm^−3^	[131]

**Table 2:** Piezoelectric properties of polymers

Polymer	Piezoelectric coefficients d*_ij_* [pC N^–1^]			Electromechanical coupling coefficients		Relative Permittivity [*ε*_r_]
	d_33_	d_31_	d_14_	k_33_	k_31_	
PVDF	−24 to −34^[^ ^143^ ^]^	8–22^[^ ^144^ ^]^ 60^[^ ^145^ ^]^	–	0.2^[^ ^146^ ^]^	0.12^[^ ^136^ ^]^	6–12^[^ ^147^ ^]^
P(VDF‐TrFE)	24 to 40^[^ ^148^, ^149^ ^]^	12^[^ ^150^ ^]^ to 25^[^ ^148^ ^]^	–	0.29^[^ ^146^ ^]^	0.16^[^ ^142^ ^]^	18^[^ ^151^ ^]^
P(VDF‐CTFE)	140^[^ ^152^ ^]^	–	–	0.36^[^ ^153^ ^]^	–	13^[^ ^154^ ^]^
P(VDF‐HFP)	24^[^ ^155^ ^]^	30^[^ ^156^ ^]^ to 43^[^ ^157^ ^]^	–	0.36^[^ ^158^ ^]^	0.187^[^ ^157^ ^]^	11^[^ ^158^ ^]^
Polyamide 11	4^[^ ^159^ ^]^	14^[^ ^160^ ^]^ (at 100–200 °C)	–	–	0.049^[^ ^136^ ^]^	5^[^ ^159^ ^]^
Polyimide	2.5–16.5^[^ ^31^ ^]^	–	–	0.048–0.15^[^ ^31^ ^]^	–	4^[^ ^61^ ^]^
Polylactic acid (PLA)	–	1.58^[^ ^161^ ^]^	9.82^[^ ^142^ ^]^	–	–	3–4^[^ ^162^ ^]^
Cellulose	5.7 ± 1.2 (cellulose nanofibril)^[^ ^163^ ^]^	1.88–30.6^[^ ^164^ ^]^	−35–60^[^ ^165^, ^166^ ^]^	–	–	–
Polyurethane	–	27.2^[^ ^167^ ^]^	–	–	–	4.8^[^ ^167^ ^]^ to 6.8^[^ ^168^ ^]^
Polyurea	19 at 60 °C^[^ ^169^ ^]^ 21 at 180 °C^[^ ^169^ ^]^	10^[^ ^142^ ^]^	–	–	0.08^[^ ^142^ ^]^	–
Polyacrylonitrile (PAN)	–	2^[^ ^136^ ^]^	–	–	–	–
Parylene‐C	2^[^ ^31^ ^]^	–	–	0.02^[^ ^31^ ^]^	–	–
Liquid crystal polymers	−70^[^ ^65^ ^]^	–	–	–	–	–

**Table 3:** Summary of piezoelectric polymer nanocomposites for energy harvesting applications

Polymer Matrix	Fillers			Nanocomposite synthesis method	Piezoelectric coefficient d_33_/d_31_ [pC N^–1^]	Poling conditions	Applied load/pressure/strain%/load resistance	Output performance			References
	Filler name	Length [*L*]/Diameter [*D*]/Thickness of fillers [*T*]	Content					Voltage [*V*]	Current/current density	Power/power density	
PVDF nanocomposites based on non‐conducting fillers											
P(VDF‐HFP)	BaTiO_3_ nanoparticles	*D* = 200nm	30 wt%	Solvent evaporation	–	100 kV cm^–1^ at 100 °C for 20 h	0.23 MPa	75	15 µA	–	[270]
P(VDF‐HFP)	BaTiO_3_ nanoparticles	*D* = 200nm	30 wt%	Solvent evaporation	180	100 kV cm^–1^ at 100 °C for 20 h	0.23 MPa	110	22 µA	0.48 W cm^–3^	[52]
PVDF	BaTiO_3_ nanoparticles	*D* = 100nm	10 wt%	Solvent evaporation assisted 3D printing	18	No poling	≈2.7 N	4	–	–	[271]
P(VDF‐TrFE)	BaTiO_3_ nanoparticles	*D* = 100 nm	0–40 wt%	Ball milling and spin casting	–	100 MV m^–1^ at RT for 6 h	0.5 N mm^–2^	9.8 at 40 wt%	0.69 µA 1.4 µA cm^–2^	13.5 µW cm^–2^	[273]
P(VDF‐TrFE)	BaTiO_3_ nanoparticles	*D* = 100 nm	0–35 wt%	Electrospinning at 18 kV	–	No poling	600 N	25 at 15 wt%	0.67 µA cm^–2^	2.28 µW cm^–2^	[274]
PVDF	BaTiO_3_ nanoparticles	*D* = 150 nm	30 wt%	Solvent evaporation	–	2 kV cm^–1^ for 8 h	10 MPa	150	1.5 µA	–	[251]
PVDF	BaTiO_3_ nanoparticles	*D* = 1 µm	30 vol%	Hot pressing and calcination	25	5 kV mm^–1^ at 120 °C for 30 min	–	–	–	–	[253]
P(VDF‐TrFE)	BaTiO_3_ nanoparticles	*D* = 10–100, L=500 nm	0–20 wt%	Ultra‐sonication	–	150 MV cm^–1^ at RT for 1 h	Finger tapping	≈0.6	≈0.5 µA	0.28 µW at 1 MΩ resistance	[275]
P(VDF‐TrFE)	BaTiO_3_ nanoparticles	*D* = 10–100, L=500 nm	0–20 wt%	Electrospinning at 20–35 kV	–	No poling	Finger tapping	5.02 at 20 wt%	–	25 µW	[276]
PVDF	BaTiO_3_ nanoparticles	–	1–10 wt%	Solvent casting	–	1 kV for 30 min at RT	100 MΩ	7.2 at 10 wt%	38 nA	0.8 µW cm^–2^	[277]
PVDF	BaTiO_3_ nanoparticles	*D* = 200 nm	0–16 wt%	Electrospinning	–	8 V for 1 h	6 mm cyclic deflection	0.48 at 16 wt%	–	–	[278]
PVDF	BaTiO_3_ fibers	*D* = 0.8 µm *L* = 4.0 µm	30 vol%	Electrospinning	11.4	35 kV mm^–1^ at 100 °C for 2 h	–	–	–	–	[279]
P(VDF‐TrFE)	Polydopamine modified BaTiO_3_ nanoparticles	*D* = 120.65 nm	20 wt%	Electrospinning at 25 kV voltage	–	No poling	700 N	6	1.5 µA	8.78 mW m^–2^	[280]
P(VDF‐TrFE)	BaTiO_3_ nanoparticles	*D* = 200 nm	0–50 wt%	Ultra‐sonication	35.3	50 MV m^–1^	0.5 MPa	13.2 at 20 wt%	0.33 µA	12.7 µW cm^–2^	[282]
PVDF	BaTiO_3_ nanoparticles	*D* = 100 nm	55 wt%	Solvent evaporation	–	15 MV m^–1^ at 100 °C for 1 h	2 N	10	2.5 µA	–	[283]
PVDF	BaTi_2_O_5_ nanorods	*L* = few microns	2.5–20 vol%	Hot pressing	–	20 kV mm^–1^ at 80 °C for 6 h.	22 MΩ acceleration = 10 g	37.5 at 5 vol%	1.7 µA	27.4 µW cm^–3^	[254]
PVDF	ZnO nanoparticles	*D* = 100 nm	1–9 wt%	Solution mixing	–	50 kV cm^–1^ at 60 °C	–	–	–	–	[284]
PVDF	ZnO nanoparticles	*D* = 70 nm	1 wt%	Solution casting	‐6.4	No poling	8.43 kPa	28	450 nA	0.4 µW	[285]
PVDF	ZnO nanoparticles	–	0.2 mol.%	Sol–gel technique	900	5 MV m^–1^ for 2 h in vacuum	1600 N m^–2^	4	–	–	[286]
PVDF	ZnO nanoparticles	*D* = 15 nm	15 wt%	Electrospinning at 16 kV	–	No poling	–	1.1	–	–	[288]
P(VDF‐TrFE)	ZnO nanoparticles	*D* = 30 ± 10 nm	1.5–12.5 wt%	Spin casting	32.2 at 7.5 wt% ZnO	No poling	65g load	7.5 V at 7.5 wt% ZnO	–	–	[289]
P(VDF‐TrFE)	ZnO nanoparticles	*D* = 20–60 nm	10 wt%	Ultrasonic mixing	19‐22	Corona poling at 14 kV for 5 min	–	–	–	–	[290]
PVDF	ZnO nanoparticles	*D* ≈ 50–150 nm	0.85 vol. %	In situ process	50	No poling	28 N	24.5	1.7 µA	32.5 mW cm^–3^	[293]
PVDF	ZnO nanorods	–	15 wt%	Ultra‐sonication and drop casting	‐1.17	No poling	15 kPa	1.81	0.57 µA	0.21 µW cm^–2^	[294]
PVDF	ZnO nanowires	*D* = 0.1 µm *L* = 2.8 µm	–	Spin coating	–	1.2 MV cm^–1^	3.2% strain	0.4	30 nA	–	[295]
P(VDF‐HFP)	Co‐doped ZnO nanorods	–	0.5–2 wt%	Electrospinning at 12 kV	–	No poling	2.5 N	2.8 at 2 wt%	–	–	[296]
P(VDF‐HFP)	Ni‐doped ZnO nanoparticles	–	0.5–2 wt%	Solution casting	20 at 0.5 wt%	No poling	2.5 N	1 at 0.5 wt%	19–21 nA	–	[297]
PVDF	Fe doped ZnO nanoparticles	Star size ≈ 1.2 µm	2 wt%	Solution casting	9.44	No poling	2.5 N	2.4	25 nA	1.17 µW cm^–2^	[298]
P(VDF‐HFP)	Cellulose NC and Fe‐doped ZnO	*W* = 44 nm *L* = 1.2 µm	2 wt% 2 wt%	Electrospinning	–	No poling	2.5 N	12	1.9 µA cm^–2^	490 µW cm^–3^	[299]
PVDF	Cellulose nanocrystals	–	1, 3, 5 wt%	Electrospinning at 15 kV	–	No poling	Force by hammer	6.3 at 5 wt%	2 µA	–	[300]
PVDF	Zirconate titanate nanoparticles	*D* ≈ 12 nm	0.5–2 wt%	Solution casting	–	No poling	16.5 kPa	25.7 at 2 wt%	1.2 µA	8.22 µW cm^–2^	[301]
PVDF	Zinc ferrite modified with TEOS	*D* = 32 nm	0.5–1.5 wt%	Drop casting and hot pressing	–	1.3 V for 2 h	Human finger impact	2.2 for 1.5 wt%	–	–	[302]
PVDF	Zinc ferrite modified with PEG	*D* = 50 nm	2–12 wt%	Drop casting and hot pressing	–	No poling	Finger tapping	18 at 12 wt%	–	–	[303]
PVDF	Zinc ferrite spheres, cubic and rod‐like	–	1–7 wt%	Drop casting and hot pressing		No poling	Finger tapping	39.1 for 3 wt% nano‐rods	–	2.96 µW mm^–3^	[304]
PVDF	GaFeO_3_ nanoparticles	–	10–30 wt%	Solvent casting	–	No poling	–	4 at 30 wt%	4 nA	–	[305]
PVDF	PZT powders	–	10–30 vol%	Solution casting	84 for 30 vol%	10 kV mm^–1^ at 80 °C in silicone oil bath	–	–	–	–	[306]
PVDF	KNN nanorods	–	0–6 wt%	Melt mixing and melt spinning	–	Corona poling at 80 °C with 15 kV voltage	Finger tapping	3.7 at 4 wt%	0.326 µA	–	[312]
PVDF	SiO_2_ nanoparticles	*D* = 20–30 nm	0.5–2 wt%	Electrospinning at 13kV voltage	–	No poling	13.9 N	24.6 at 0.5 wt% SiO_2_	–	–	[313]
PVDF	NiO nanoparticles	*D* = 44 nm	0.25–1 wt%	Solution casting	–	No poling	–	–	–	–	[317]
PVDF	SiO_2_ coated NiO nanoparticles	*D* = 3 µm	1–15 wt%	Solution casting	–	No poling	0.3 MPa	53	≈0.3 µA cm^–2^	685 W m^–3^	[318]
P(VDF‐TrFE)	MgO nanoparticles	*D* < 50 nm	0–8 wt%	Solution casting	−65 at 2 wt%	No poling	Finger tapping	2	–	–	[319]
PVDF	CoFe_2_O_4_ nanoparticles	*D* = 30–70 nm	5 wt%	Ultra‐sonication and solvent evaporation	33	Corona poling at 80 °C for 0.5 h	–	–	–	–	[320]
PVDF	Fe_3_O_4_ nanoparticles	*D* = 6 nm	0.5–2 wt%	Solution mixing	37 at 2 wt%	35 MV m^–1^ at 60°C for 1 h					[321]
PVDF	Ce‐doped Fe_2_O_3_ Ce‐doped Co_3_O_4_ nanoparticles	*D* = 39 nm *D* = 29 nm	2 wt%	Electrospinning at 12 kV	–	No poling	2.5 N	20 15	0.010 0.005 µA cm^–2^	700.64 334.39 µA cm^–3^	[323]
P(VDF‐HFP)	Li doped montmorillonite (Mt)	–	7–50 wt%	Solution casting	45 at 15 wt%	No poling	Pressing by fingers	5	50 nA	–	[328]
PVDF	Micro‐CaCO_3_ and Mt particles	–	30–40 wt% 0–3 wt%	Twin screw extrusion and stretching	30.6 at 40 wt% CaCO_3_ and 3 wt% Mt	Corona poling at 90 V µm^–1^, RT for 10 min	0.98 N	–	–	–	[329]
PVDF	Laponite nano‐clay	–	0.1–0.5 wt%	Solvent evaporation	–	No poling	300 N	6 at 0.5 wt%	70 nA	–	[330]
PVDF	Nano‐clay	Fiber *D* = 330 ± 30 nm	0–20 wt%	Electrospinning at 12.5 kV	–	No poling	Finger tapping	70 at 15 wt%	≈20 nA	68 mW cm^–2^	[331]
PVDF	Halloysite nanotubes	–	10 wt%	Electrospinning at 20 kV	–	–	–	Highest output voltage	–	–	[332]
PVDF	Talc nanoparticles	*D* < 100 nm	0.25–1 wt%	Electrospinning at 18 kV	–	No poling	3.8 N	9.1 at 0.5 wt% talc	16.5 nA	1.12 µW cm^–2^	[333]
PVDF	Gd_5_Si_4_ nanoparticles	*D* = 470 ± 129 nm	2.5 and 5 wt%	Spin casting via phase inversion	–	No poling	2–3 N	≈1.2 at 5 wt%	–	–	[334]
PVDF	MoS_2_ nanosheets	–	–	Electrospinning	–	No poling	Finger touch	14	8 nA	–	[335]
PVDF nanocomposites based on conducting fillers											
P(VDF‐TrFE)	Ag nanoparticles	–	0.005–1 vol%	Sonication and tape casting	20.23 at 0.005 vol%	No poling	–	–	–	–	[336]
P(VDF‐TrFE)	Ag nanoparticles and Ag nanowires	*D* = 20 nm *D* = 120, 35 nm	0.4–1.2 wt%	Ultrasonication	–	No poling	–	0.07 at 1.2 wt% Ag NWs	–	–	[339]
PVDF	Ag nanoparticles	*D* = 25–40 nm	0–1 wt%	Electrospinning	–	No poling	1 MΩ load resistance	2 at 0.4 wt%	2 µA	–	[340]
PVDF	Ag nanowires	*D* = 40 nm	0–3 wt%	Electrospinning at 12 kV	30 at 1.5 wt%	No poling	–	–	–	–	[341]
PVDF	Pt nanoparticles	*D* = 600 nm of Pt/PVDF Nfs	1.5 wt%	Electrospinning at 150 kVm^–1^	44	No poling	0.3 MPa	30	6 mA cm^–2^	22 µW cm^–2^	[342]
PVDF	SnO_2_ nanosheets	Thickness ≈ 100 ± 5 µm	5 wt%	Solution casting	36.52	No poling	0.3 MPa	42	6.25 µA cm^–2^	4900 W m^–3^	[343]
PVDF	MWCNTs	*D* = 10–15 nm	0.05–1 wt%	Electrospinning at 14 kV	–	500 kV cm^–1^ at 120°C for 20 min in silicone oil bath	–	–	–	–	[263, 337]
PVDF	MWCNTs	–	0–0.05 wt%	Near field Electrospinning	−57.6	In situ poling at 1200 V mm^–1^	–	–	–	–	[203, 345]
PVDF	MWCNTs	–	3–10 wt%	Electrospinning at 18 kV	–	No poling	4 MPa	6 at 5 wt%	–	81.8 nW	[347]
PVDF	CNTs	*D* = 10–20 nm	18 wt%	Electrospinning	31.3	No poling	350 N	1.89	11 nA	–	[348]
PVDF	MWCNTs	*D* = 40–90 nm AR > 100	0–0.3 wt%	Solution casting	–	Stepwise poling at 60 MV m^–1^	–	3.7 at 0.05 wt%	–	–	[262]
PVDF	MWCNTs functionalized with IL	*D* = 20–30 nm *L* = 0.5–200 µm	0.05–1 wt%	Solvent casting and melt blending	–	–	–	–	–	–	[349]
PVDF	MWCNTs functionalized with carboxyl, amino & hydroxyl groups	*D* = 9.5 nm *L* ≈ 1 µm	1.5–5 wt%	Melt mixing	–	–	–	–	–	–	[350]
PVDF	COOH functionalized CNTs & Ag‐CNTs	*D* = 1.3–1.9 µm of fibers	1 wt%	Electrospinning at 15 kV	54 pm V^–1^ for Ag‐CNTs	No poling	–	–	–	–	[351]
PVDF	Unzipped MWCNTs	*D* = 30 nm *L* = 10 µm	0.3 wt%	Solution coagulation	38.4	No poling	–	–	–	–	[352]
PVDF	MWCNTs coated with TiO_2_	*D* = 40–60 nm *L* = 5–15 µm	0–1 wt%	Solution casting	41 at 0.3 wt%	120 V µm^–1^ at 70 °C for 1.2 h	–	–	–	–	[354]
PVDF	Buckminster fullerenes (C_60_) and SWCNTs	–	0–0.25 wt%	Ultrasonication	65 at 0.05 wt% SWCNT	20 kV at 80 °C for 20 min	0.46 N	–	–	–	[355]
P(VDF‐TrFE)	Graphene	–	0–0.15 wt%	Solution casting	34.3 ± 7.2	Stepwise from 10 to 60 MV m^–1^, 10 MV m^–1^ per step	20.37 MΩ	12.43 at 0.15 wt%	0.6 µA	148.06 W m^–3^	[357]
PVDF	Graphene nanoplatelets	–	0–5 wt%	Electrospinning at 20 kV	–	No poling	–	7.9 at 0.1 wt%	4.5 µA	–	[358]
PVDF	Graphene nanoplatelets	*L* = 2–10 µm	2–5 wt%	Solution casting	–	No poling	–	–	–	–	[359]
PVDF	Graphite nanosheets	*T* < 200 nm	1–7 mL	Solution casting	6.7 at 6 mL	50 kV mm^–1^ for 30 min at 130 °C	–	–	–	–	[360]
PVDF	Ce^3+^ doped Graphene	*D* = 80 nm of nanofibers	0.2 wt% 1 wt%	Electrospinning at 12 kV	–	No poling	6.6 kPa	11	0.07 µA	0.56 µW cm^–2^	[361]
PVDF	Graphene‐Ag doped nanosheets	*D* = 55 nm for Ag NPs	–	Solution casting	–	No poling	5.2 kPa	0.1	0.1 nA	–	[362]
PVDF	PMMA functionalized graphene	*T* = 2–4.5 nm	0.5–5 wt%	Sonication and solvent evaporation	–	No poling	–	–	–	–	[261]
PVDF	Graphene and polybenzoxazole	*D* = 60 µm	0.3 wt%	Electrospinning at 16 kV	–	No poling	–	60	–	–	[363]
PVDF	Reduced graphene oxide (rGO)	–	0–0.2 wt%	Solution casting	–	Stepwise poling at 60 MV m^–1^ at 8 min intervals	Vibration test at 30 Hz	3.28 at 0.05 wt%	–	–	[338]
PVDF	Reduced graphene oxide	*T* = 1 nm *L* = 100–600 nm	0.1–0.3 wt%	Ultrasonication and hot pressing	–	Corona poling at 12 kV, 60 °C for 30 min	–	1.3 V at 0.1 wt%	–	36 nW at 704 kΩ resistance	[364, 365]
PVDF	Reduced graphene oxide	*D* = 10–15 nm	0.1–1 wt%	Ultrasonication and compression molding	–	–	500 g	0.45 at 1 wt% rGO	0.15 µA	14 µW cm^–3^	[366]
P(VDF‐TrFE)	Reduced graphene oxide	*W* = 80 nm *L* = 300 nm	0–0.2 wt%	Drop casting	−23 at 0.1 wt%	No poling	3.2 µW	2.4 at 0.1 wt%	0.8	2 N	[367]
PVDF	Fe doped rGO	–	0.1–2 wt%	Ultrasonication	–	No poling	12 kPa	5.1 at 2 wt% Fe‐rGO	0.254 µA	–	[368]
PVDF	Ag doped rGO	–	0.1–2 wt%	Ultrasonication and centrifugation	–	No poling	1 MΩ load resistance	18 at 1 wt% Ag‐rGO	1.05 µA	28 W m^–3^	[369]
PVDF	ZnO doped rGO	–	rGO/ZnO ratio 1:1, 2:1, 4:1	Solution casting	–	No poling	–	–	–	–	[370]
PVDF	AlO doped rGO	*D* ≈ 30–40 nm	1 wt%	Solution casting	45	No poling	31.19 kPa	36	0.8 µA	27.97 µW cm^–3^	[371]
PVDF	CdS doped rGO	–	0.25 wt%	Electrospinning	–	No poling	Finger imparting	4	–	–	[372]
PVDF	Fe‐rGO and CNTs	–	–	–	–	No poling	Finger excitation	2.5 for rGO 1.2 for CNTs	0.7 µA 0.3 µA	–	[373]
PVDF	Graphene oxide nanosheets	*T* ≈ 1 nm *L* ≈ 100–800 nm	0.05–2 wt%	Solution casting	–	No poling	–	–	–	–	[374]
PVDF	Graphene oxide	*T* ≈ 13 nm *L* ≈ 0.3 µm	2 wt%	Drop casting	–	No poling	–	–	–	–	[377]
PVDF	Graphene oxide	*T* = 0.7–1.4 nm *L* = 5–100 µm	0–5 wt%	Non‐solvent induced phase separation	–	No poling	–	2.64 at 0.5 wt%	–	–	[260]
PVDF	Carboxylated and fluorinated graphene oxide	*D* = 600–700 nm	1 wt%	Electrospinning at 16 kV	63 for fluorinated GO	No poling	–	–	–	–	[378]
P(VDF‐HFP)	Carbon black nanoparticles	*D* ≈ 36 nm	0–0.8 wt%	Solution casting	–	Poling at 90 MV m^–1^	–	3.68 at 0.5 wt%	–	13 W m^–3^	[264]
P(VDF‐HFP)	Carbon black (CB) and few layer graphene (FLG)	*D* = 50–100 nm *D* = 0.5–5 nm	0–0.8 wt% 0–0.03 wt%	Ultrasonication	–	Stepwise from 20 to 90 MV m^–1^, 10 MV m^–1^ per step	2 MΩ	4.1 at 0.5 wt% CB and 0.02 wt% FLG	2 µA	51.9 W m^–3^	[265]
PVDF nanocomposites based on conducting and non‐conducting filler combination											
PVDF	BaTiO_3_ nanoparticles and MWCNTs	*D* = 700 nm *D* = 8–15 nm *L* = 10–50 µm	18 wt% 0.4 wt%	FDM 3D printing	d_31_ = 0.13 at 0.4 wt% MWCNT 18 wt% BaTiO_3_	5.4 MV m^–1^ for 15 h	80 N	0.43	0.94 nA	–	[379]
P(VDF‐HFP)	BaTiO_3_ NPs and hexagonal boron nitride nanolayers	–	3 wt% BaTiO_3_ 1 wt% h‐BN	Solution mixing	–	–	–	2.4	–	–	[380]
PVDF	BaTiO_3_and graphene quantum dots	*D* < 100 nm	2 wt% 1.5 wt%	Spin casting	–	No poling	265 mN	4.6	4.13 pA cm^–2^	11.2 µW cm^–3^	[381]
PVDF	TiO_2_ nanolayers and rGO	*D* = 15 nm *T* = 1.5 nm	2.5 wt% of each	Solution mixing	–	No poling	–	–	–	–	[382]
PVDF	TiO_2_ nanotubes and CNT	–	1–5 wt%	Solution casting	–	Corona poling at 8 kV for 7 s	2.5 N	1.3 at 1 wt%	–	–	[383]
P(VDF‐HFP)	TiO_2_‐rGO nanotubes and SrTiO_3_ nanoparticles	SrTiO_3_ *D* < 100 nm	1 wt% TiO_2_‐rGO 2 wt% SrTiO_3_	Solvent casting	7.52	Corona poling	–	2	–	–	[267]
PVDF	NaNbO_3_ nanorods and rGO	*L* = 200, *D* = 50 nm *T* ≈ few layers	0.1 wt%	Ultrasonic mixing	–	–	15 kPa	2.16	0.383 µA	–	[266]
PVDF	Fe_3_O_4_‐Graphene oxide nanoparticles‐nanosheets	Fiber *D* = 117–710 nm	0–2 wt%	Solution mixing and Electrospinning at 12 kV	1.75 at 2 wt%	No poling	1.32 N	0.23 at 2 wt%	–	–	[385]
PVDF	TiO_2_‐Fe_3_O_4_‐MWCNT nanotubes	Fiber *D* = 70 µm	0–2 wt%	Electrospinning at 12 kV	51.42 at 2 wt%	No poling	1.32 N	0.68 at 2 wt%	–	–	[386]
PVDF	MnO_2_/graphene/MWCNT hybrid	*D* = 240–300 nm	0.2–1 wt%	Solution casting and rolling	17‐33	50–80 MV m^–1^	–	–	–	–	[268]
PVDF	Graphene oxide, graphene, halloysite nanotubes	*D* = 3.4–7 nm *D* = 2–18 nm *D* = 30–70 nm, *L* = 2–3 µm	0.05–3.2 wt%	Electrospinning	5 for 0.8 halloysite	No poling	0.49 N	0.1 for 0.8 halloysite	0.1 µA	–	[387]
PVDF	PMN‐PT particles CNTs	–	30 vol% 1 vol%	Magnetic stirring and heat treatment	–	–	–	4	30 nA	–	[389]
PVDF	Cellulose rods Carbon nanotubes kaolinite clay nanoparticles	*D* = 15–30 µm; *L* = 124–400 µm; *D* = 10–30 nm *L* = 2–3 µm; *D* = 1.2 µm	0.5–2 wt%	Solution casting	–	No poling	–	–	–	–	[390]
PDMS nanocomposites based on non‐conducting fillers											
PDMS	BaTiO_3_ nanowires	*L* ≈ 4 µm *D* ≈ 156 nm	5–20 wt%	Spin casting	–	*E* = 0.5–1.5 kV at 140 °C for 12 h	Bending and unbending	7	360 nA	≈1.2 µW at 20 MΩ	[392]
PDMS	BaTiO_3_ nanoparticles and nanowires	*D* = 120 nm *L* = few microns	13 wt%	Magnetic stirring and spin casting	–	1 kV at 120 °C for 12 h	Bending and unbending	60	1.1 µA	40 µW at 500 MΩ	[393]
PDMS	BaTiO_3_ nanotubes	*D* = 11.8 nm *L* = 4.1 µm	1–4 wt%	Spin casting	–	80 kV cm^–1^ at ambient temp. for 12 h	1 MPa	5.5	350 nA *j* = 350 nA cm^–2^	–	[394]
PDMS	BaTiO_3_ nanofibers	*D* = 0.7–0.9 nm *L* = 1.7 mm	31 wt%	Ultrasonication and curing	–	5 kV mm^–1^ at 120 °C for 12 h	0.002 MPa	2.67	261.4 nA	0.1841 µW	[395]
PDMS	BaTiO_3_ nanocubes	*D* = 100–400 nm	10–25 wt%	Solution casting	–	8 kV at RT for 24 h	988.14 Pa	126.3 at 15 wt%	77.6 µA cm^–2^	≈7 mW cm^–2^ at 100 MΩ	[396]
PDMS	BaTiO_3_ nanocrystals	*D* = 50–100 nm	20 wt%	Solution casting and curing	–	2 kV at 130 °C for 12 h	–	6	300 nA	–	[397]
PDMS	ZnSO_3_ nanocubes	Edge size = 100–200 nm	10–60 wt%	Centrifugal mixing	–	No poling	0.91% strain	12 at 40 wt%	0.89 µA cm^–2^ at 40 wt%	–	[398]
PDMS	Li doped ZnO nanowires	–	10 wt%	Spin casting and curing	–	105 kV cm^–1^ at 65 °C for 20 h	0.91% strain	180	50 µA	–	[399]
PDMS	NaNbO_3_ nanowires	*D* ≈ 200 nm *L* ≈ 10 µm	1 vol%	Spin coating	–	≈80 kV cm^–1^ at RT	0.23% strain	3.2	72 nA 16 nA cm^–2^	0.6 mW cm^–3^	[401]
PDMS	LiNbO_3_ nanowires	*D* ≈ 100–250 nm *L* ≈ 50 µm	1 vol%	Mixing and spin coating	25 for LiNbO_3_ nanowires	100 kV cm^–1^ at RT	10^5^ strain cycles	0.46	0.009	–	[41]
PDMS	PZT nanotubes	*L* = 59 mm; *D* = 210 nm	1:100 vol. ratio	Mixing	–	Corona poling at 1.5 kV	980 N m^−1^	1.52	54.5 nA	37 nW cm^–2^	[44]
PDMS	ZnO or PZT hemispheres	*D* = 10 µm	–	LB deposition and magnetron sputtering	–	No poling	0.425% strain	6 3	0.2 µA cm^–2^ 0.05 µA cm^–2^	–	[402]
PDMS	PMN‐PT nanowires	*L* ≈ 10 µm	10 wt%	Mechanical mixing and curing	–	5 kV mm^–1^ at 150 °C in silicone oil bath for 24 h	–	7.8	2.29 µA	–	[403]
PDMS	BiFeO_3_ nanoparticles	–	10–40 wt%	Mixing and spin casting	–	200 kV cm^–1^ at 150 °C for 10 h	10 kPa	3 at 40 wt%	0.25 µA at 40 wt%	–	[64]
PDMS	Cellulose nanofibril	–	0.85 wt%	Spin coating	19	No poling	0.05 MPa	60.2	10.1 µA	6.3 mW cm^–3^	[405]
PDMS	FAPbBr_3_ nanoparticles	*D* = 50–80 nm	35 wt%	Centrifugal mixing	–	50 kV cm^–1^ in dry the atmosphere at RT for 12 h.	0.5 MPa	8.5	3.8 µA cm^–2^	12 µW cm^−2^ at 800 kΩ	[407]
PDMS	Aurivillius‐based oxide [CaBi_4_Ti_4_O_15_]	*D* = 10 mm *T* = 1 mm	1–12 wt%	Solution casting	–	2 kV for 1 h in silicon oil bath	300 MΩ	23 at 8 wt%	85 nA	1.09 mW m^–2^	[411]
PDMS nanocomposites based on conducting and non‐conducting filler combination											
PDMS	BaTiO_3_ NPs, SW/MWCNTs or rGO	*D* = 100 nm *D* = 5–20 nm, *L* ≈ 10 µm	12 wt% 1 wt%	Ultrasonic mixing and spin casting	–	100 kV cm^–1^ at 150 °C for 20 h	57 kPa	3.2 2	350 nA	–	[125]
PDMS	BaTiO_3_ nanofibers and MWCNTs	*L* ≈ 33.7 mm, *D* ≈ 354.1 nm 10–15 nm, *L* = 10–20 µm	10–50 wt% 0–5 wt%	Ultrasonic mixing and spin casting	–	5 kV mm^–1^ for 12 h in silicone oil bath	2 kPa	3 for 30‐BaTiO_3_/0.5‐CNTs 3.73 for 40‐BaTiO_3_/2‐CNTs	0.82 µA 1.37 µA	0.14 µW 0.33 µW	[412]
PDMS	BaTiO_3_ nanoparticles and carbon black	*D* = 1.2 ± 0.6 µm *D* = 30 nm	30 wt% 0–4.8 wt%	Ball milling and mixing	15.3	30 kV mm^–1^ at 100 °C for 20 h	Periodic beating by vibrator	7.43 at 3.2 wt% C	5.13	1.98 µW cm^–2^	[413]
PDMS	ZnO nanoparticles and MWCNTs	*D* = 30 nm *D* = 8–15 nm, *L* = 50 µm	12 wt% 1 wt%	Mechanical mixing	–	No poling	–	7.5	2500 nA	18.75 µW at 5.64 MΩ	[414]
PDMS	PZT and MWCNTs	*D* ≈ 1 µm *D* ≈ 15nm, *L* ≈ 10 µm	12 wt% 1 wt%	Ball milling and stirring	–	2 kV at 140 °C for 12 h	Periodic stress by a linear motor	100	10	–	[415]
Eco flex silicone rubber	PMN‐PT and MWCNTs	*D* ≈ 1 µm *D* ≈ 20 nm, *L* ≈ 10 µm	20 wt%	Blending and curing	–	50 kV cm^–1^ at 110 °C	Twisting, folding, pressing	4	500 nA	–	[416]
PDMS	KNLN particles and Cu nanorods	*D* = 1–3 µm *D* = 200–400 nm, *L* ≈ 5 µm	–	Magnetic stirring, spin coating + curing	–	2 kV at 150 °C for 12 h	Bending and un‐bending by linear motor	12	1.2 µA	–	[418]
PDMS	Cellulose microfiber and MWCNTs	*D* = 10 µm *D* = 9.5 nm, *L* = 1.5 µm	5 wt% 0.5 wt%	Mechanical agitation	15	No poling	40 kPa	30	500 nA	9 µW cm^–3^	[419]

This paper aims to present a holistic review of the recent developments in piezoelectric nanostructured materials, polymers, polymer nanocomposites, and piezoelectric films for implementation in energy harvesting. The paper is structured into nine sections. Following the introduction, the second section covers the theory and mechanism of piezoelectricity. The third section covers fabrication methods, piezoelectric properties, energy harvesting performance, and mechanisms of piezoelectric nanogenerators (PENGs) built using nanostructured materials. In the fourth section, the piezoelectric properties of PVDF based polymers and their copolymers along with other polymers such as, polylactic acid (PLA), polyureas, polyamides, polyacrylonitrile (PAN) are outlined. The fifth section is dedicated to the synthesis, piezoelectric properties, mechanisms, and energy harvesting capabilities of PVDF and polydimethylsiloxane (PDMS) based polymer nanocomposites prepared using different types of fillers. The sixth section discusses the piezoelectric performance of PVDF films doped with inorganic salts such as magnesium chloride, nickel chloride, iron nitrate, and zinc nitrate. The seventh section is a brief overview of energy harvesting using piezoelectric films. The eighth section describes some examples of the application of PEHs for wireless devices and self‐powered sensors. The last section summarizes the paper and provides insight into the current challenges and future perspectives of piezoelectric energy harvesting.

## 2 Piezoelectric Effect: Theory and Mechanism

**Figure 1:**
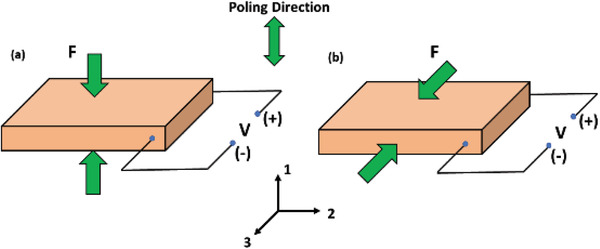
Operating modes of a piezoelectric material a) 33 mode and b) 31 mode.

Pierre Curie and Jacques Curie were the pioneers who discovered the phenomenon of piezoelectricity in 1880 while conducting studies in crystals of quartz, tourmaline, and Rochelle salt.^[^ ^53^ ^]^ There are two distinct piezoelectric effects, namely, the direct effect and inverse effect. In the direct piezoelectric effect, a material is polarized and produces voltage under an applied tensile or compressive stress. In the inverse effect, the application of electric potential induces mechanical displacement in the material. The constitutive Equations (1) and (2) for both the effects are given below.^[^ ^54^, ^55^ ^]^

(1) $$\mathrm{Direct}\;\mathrm{Effect}:S_{i}=s_{ij}^{E}\;T_{j}\;+\;d_{im}E_{m\;}\;$$

(2) $$\mathrm{Inverse}\;\mathrm{Effect}:D_{k}=d_{jk}\;T_{j}+\;\varepsilon_{km}^{T}E_{m\;}\;$$

where *T* = stress, *d* = piezoelectric constant, *S* = strain, *D* = electric displacement, and *E* = electric field. *s^E^* is the mechanical compliance at constant electric field *E*, ɛ^*T*^ is the permittivity of the material at constant stress *T*. The subscripts *i, j*, and *k* refers to the different directions in the material coordinate system. They are analogous to the Cartesian coordinate axes (*x, y, z*) and have a value of 1 to 3. The rotational motion around the three axes (1, 2, and 3) is denoted by subscript “*m*,” hence it has a value of 1 to 6. A piezoelectric energy harvesting device has two primary operating modes: mode 33, in which applied stress is in the direction of polarization (**Figure** **1**a) and mode 31, in which applied stress is perpendicular to the direction of polarization^[^ ^54^, ^56^ ^]^ (Figure 1b). The shear mode denoted by 14 is less commonly used.

**Figure 2:**
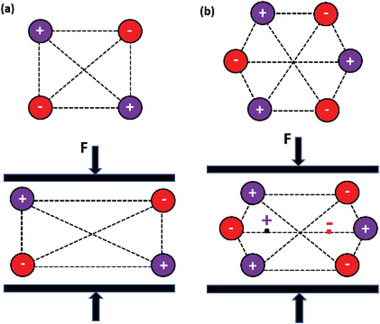
Schematic of 2 D crystal structures. a) Non‐piezoelectric square. b) Piezoelectric hexagon.

The piezoelectric effect originates from the distribution of ions in the crystalline structure of certain materials. In the absence of an external force, there exists a steady‐state equilibrium between the positive and negative electric charges in the material, therefore it remains neutral. When a square‐shaped structure is subjected to compressive stress (**Figure** **2**a), the equivalent center of charge is still at the same point, hence there is no change in polarization. For a 2 D hexagon (Figure 2b), when stress is applied, a change is triggered in the center of charge of the cations and anions that induces a change in polarization. There are 32 crystallographic classes, out of which 21 are non‐centrosymmetric (lacking center of symmetry) and 20 of them exhibit direct piezoelectricity; the 21st being the cubic class.^[^ ^57^ ^]^ In these materials, due to the absence of symmetry in the ion distribution, electrical dipoles are present, resulting in a piezoelectric response. This behavior is seen in materials such as aluminum nitride and zinc oxide (ZnO).

**Figure 3:**
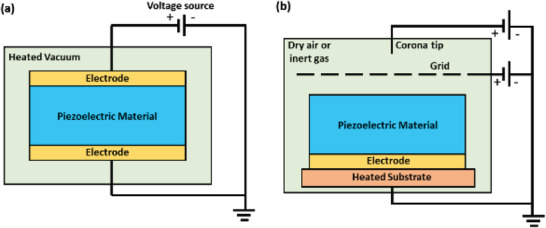
Schematic diagram showing the poling systems. a) Electrode poling. b) Corona poling.

In most other materials, the molecular dipoles are randomly oriented within their crystal structure. To obtain an effective piezoelectric response from such materials, an important operation called “poling” is done. In the process of poling, the molecular dipoles in a material are re‐oriented by exerting a high electric field at high temperature, followed by subsequent cooling keeping the same electric field to sustain the orientation state. The two well‐known methods of poling are electrode poling and corona poling. A high voltage is applied to the piezoelectric material in electrode poling by pressing conductive electrodes on two sides of the material^[^ ^58^ ^]^ (**Figure** **3**a). An electric field in the range of 5–100 MV m^–1^ is usually applied.^[^ ^59^, ^60^, ^61^, ^62^ ^]^ In the corona poling process (Figure 3b), a needle with high conductivity is maintained at extremely high voltage (8–20 kV) and is located on a grid at a lower voltage (0.2–3 kV). The piezoelectric material is situated beneath the grid and is kept in an atmosphere of dry air or inert gas.^[^ ^59^, ^61^, ^63^ ^]^ Due to ionization around the corona tip, the gas molecules get accelerated toward the piezoelectric material surface. The bottom side of the material is covered with an electrode, which is in contact with a hot substrate to achieve better control over poling. The poling temperature does not exceed 300 °C for both methods in all material cases.^[^ ^31^ ^]^

### 2.1 Piezoelectric Effect: Utilization in Energy Harvesting

**Figure 4:**
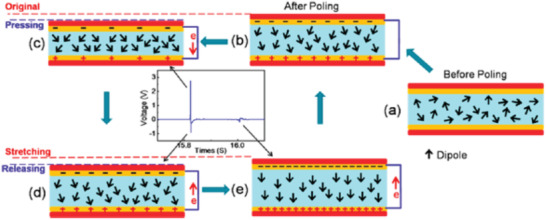
Diagram showing the working mechanism of an energy harvester. Reproduced with permission.^[^ ^64^ ^]^ Copyright 2016, American Chemical Society.

In energy harvesting, the direct piezoelectric effect is utilized, wherein an applied force leads to electrical charge generation. **Figure** **4** schematically shows the working mechanism of an energy harvester. Initially, in the absence of poling, the dipoles present in the piezoelectric material align randomly between two electrodes^[^ ^64^ ^]^ (Figure 4a). Upon application of an electrical field to the energy harvester, the dipoles align in the same direction as the applied field (Figure 4b). In the absence of external force, zero electric signal is acquired by the device because it remains in a state of equilibrium. Under the application of compressive force in the vertical direction, the material is polarized due to compressive strain, inducing piezoelectric potential between the electrodes. In the course of this process, an output signal is acquired (Figure 4c). On releasing the applied force, a slight tensile force develops that induces a reverse piezoelectric potential (Figure 4d,e).

For energy harvesting, when mechanical stress (Δ*σ*) is applied, the charge (*Q*) generated across the opposite faces of a piezoelectric material of area (*A*) is defined by Equation (3).^[^ ^65^ ^]^

(3) $$Q=d_{33}\times A\times \Delta \sigma$$

The material's piezoelectric coefficient (pC N^–1^) in 33‐mode is denoted by d_33_. Load impedance is infinite under open‐circuit conditions, hence the equation *Q = CV* is used, where *C* is the capacitance of the material ($C=A\frac{\varepsilon_{33}^{T}}{h}$) and is used to calculate voltage (*V*) from Equation (4).

(4) $$V=\frac{d_{33}}{\varepsilon_{33}^{T}}\times h\times \Delta \sigma$$

where “*h*” is material thickness and $\varepsilon_{33}^{T}$ is the permittivity at a constant stress value in 33‐mode. By substituting “*V*” from Equation (4) in the expression $\frac{1}{2}CV^{2}$, the energy *E* due to applied stress is defined by Equation (5).

(5) $$E=\frac{1}{2}\times C\frac{d_{33}^{2}}{\varepsilon_{33}^{T}}\times A\times t\times \left(\Delta \sigma^{2}\right)$$

Therefore, for a certain thickness and area, the energy obtained from a piezoelectric device can be maximized by choosing piezoelectric materials with large$\frac{d_{33}^{2}}{\varepsilon_{33}^{T}};$ it is known as harvesting Figure of merit. Under short circuit conditions, the current (*I*) is given by $I=\frac{\Delta Q}{\Delta t}$(Equation (3)) and can be written as Equation (6).

(6) $$I=d_{33}\times A\times \frac{\Delta \sigma }{\Delta t}$$

In energy harvesting applications, open and closed‐circuit measurements are frequently performed. However, there is no effective power at these conditions because the current is zero at the open circuit and there is no potential difference at the closed circuit. The instantaneous power density is calculated using the formula $p\;=\frac{V^{2}}{R\times A}\;$,^[^ ^66^, ^67^, ^68^ ^]^ where *A* is the effective area of the electrodes and the voltage across load resistance *R* is denoted by *V*.

There is a coupling coefficient, *k*, which denotes the efficiency of energy conversion in generator mode and is given by Equation (7).^[^ ^54^, ^69^ ^]^

(7) $$k^{2}=\frac{\mathrm{transformed}\;\mathrm{energy}}{\mathrm{incoming}\;\mathrm{energy}}$$

The coupling coefficient in 33‐mode and 31‐mode is denoted by $k_{33}=\frac{d_{33}}{\sqrt{\varepsilon_{33}^{T}s_{33}^{E}}}$ and$k_{31}=\frac{d_{31}}{\sqrt{\varepsilon_{33}^{T}s_{31}^{E}}}$. The greater the coupling coefficient k, the higher the mechanical energy that can be scavenged.

## 3 Piezoelectric Nanostructured Materials

In the last decade, piezoelectric nanostructured materials for energy harvesting applications have expanded rapidly.^[^ ^70^, ^71^ ^]^ The majority of the reported work is on ZnO because its nanostructures form easily at low temperatures and are crystallographically aligned.^[^ ^72^ ^]^ Recently, other materials such as PZT, BaTiO_3_, and PMN‐PT have been explored as well for nanostructured energy harvesters motivated by their high piezoelectric coefficients. This section summarizes the synthesis techniques, piezoelectric properties, and energy harvesting performance of nanostructured materials.

### 3.1 1D ZnO Nanostructures

ZnO has diverse nanostructures and exhibits both semiconducting and piezoelectric properties because of its non‐centrosymmetric crystal structure,^[^ ^73^, ^74^, ^75^, ^76^ ^]^ comprising alternating planes of O^2–^ and Zn^2+^ ions in tetrahedral coordination piled up along the *c*‐axis.^[^ ^74^ ^]^ For ZnO, the piezoelectric constant d_33_ has been reported in the range of 10–12 pC N^–1^.^[^ ^77^, ^78^ ^]^ The first study was reported by Z. L. Wang et al., in 2006,^[^ ^73^ ^]^ wherein aligned ZnO nanowire (NW) arrays were grown on Al_2_O_3_ support via a vapor–liquid–solid based process, utilizing gold (Au) as a catalyst.^[^ ^73^, ^79^ ^]^ Atomic force microscopy (AFM) using a‐Si tip coated with Pt was used to conduct piezoelectricity measurements. The majority of the Au particles on the NW tips either evaporated during the growth or dropped off when deflected by the AFM tip. The grown NW arrays had smaller lengths ranging between 0.2 and 0.5 µm and comparatively lower densities, enabling the AFM tip to solely reach a single NW without interfering with another. The mechanism of power generation was attributed to two factors‐ first, strain field generation and charge detachment throughout the NWs due to bending by AFM tip and second, Schottky barrier formed between the AFM tip and the ZnO NWs. Similarly, Lu et al. synthesized phosphorus‐doped ZnO NWs on Si substrates that produced electricity when bent by an AFM tip.^[^ ^80^ ^]^ A voltage of up to 50–90 mV was generated by the p‐type ZnO NWs, whereas the n‐type ZnO NWs generated negative output in the range of −5 to −10 mV.

Yang et al. demonstrated the transformation of biomechanical energy from the movement of a human finger and live hamster into electrical energy with the help of a ZnO NW based nanogenerator (NG).^[^ ^81^ ^]^ A single wire nanogenerator (SWG) was fabricated by firmly attaching the two ends of a ZnO NW to metal electrodes packaged on a flexible polyimide substrate.^[^ ^82^, ^83^ ^]^ A higher output voltage was obtained by integrating multiple SWGs, resulting in a voltage of ≈0.1–0.15 V using four SWGs in series.

**Figure 5:**
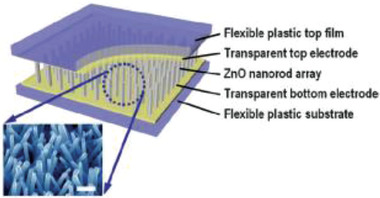
Schematic diagram of the PENG and FE‐SEM image of ZnO NR arrays grown on ITO coated PES substrate (scale bar: 300 nm). Reproduced with permission.^[^ ^84^ ^]^ Copyright 2009, Wiley‐VCH.

Choi et al., in 2009, reported a ZnO nanostructure‐based completely flexible PENG for applications in self‐powered sensors for the first time.^[^ ^84^ ^]^ ZnO nanorods (NRs) were synthesized from a solution of zinc nitrate hexahydrate [Zn(NO_3_)_2_.6H_2_O] and hexamethylenetetramine (HMTA) at 95 °C. A flexible polyethersulfone (PES) substrate coated with indium tin oxide (ITO) was utilized to grow the NR arrays (**Figure** **5**). Top electrodes comprising of ITO coated PES with and without palladium gold (PdAu) film were located on top of the ZnO NR arrays as shown in Figure 5. An output current density of *j* ≈ 1 µA cm^–2^ was obtained from the PENG of dimensions 3 cm x 3 cm, when compressed by a force of 0.9 kgf. It was inferred that the Schottky contact between the ZnO NRs and top electrodes helped in the high current generation as proposed in previous studies.^[^ ^73^ ^]^ In the next year, the same group reported a transparent, flexible PENG using single‐walled carbon nanotube (SWCNT) as the top electrode,^[^ ^85^ ^]^ with a current density of about five times that of ITO based PENG.^[^ ^84^ ^]^ The surface of CNT films had a nanosized network with a pore size greater than 100 nm, which favored the growth of ZnO NRs and increased current generation from the PENG. Later they used graphene sheets as transparent electrodes that were synthesized via chemical vapor deposition (CVD) for a fully rollable and transparent PENG.^[^ ^86^ ^]^ Vertically aligned ZnO NRs were grown on graphene sheets via low‐temperature hydrothermal technique as described above. The diameter of the ZnO NRs was <100 nm, the length was ≈2 µm and growth density was about 20 µm^–2^. The PENG produced a *j* value = 2 µA cm^–2^ and it was illustrated to be stable and reliable under external loads such as rolling and bending.

A five‐layered PENG structure was fabricated by Hu et al., consisting of ZnO NW films on the top and bottom surfaces of flexible polymer substrate and electrodes attached to the ZnO NWs.^[^ ^87^ ^]^ When the PENG was strained to 0.12% at a strain rate of 3.56% s^–1^, the outputs obtained were 10 V and 0.6 µA. It was shown to drive an autonomous wireless system for long‐distance data transmission. Later on, he improved the PENG's performance significantly by pre‐treating ZnO NWs with oxygen plasma, annealing in the presence of air and passivating their surface with specific polymers.^[^ ^88^ ^]^ A maximum output of 20 V and 6 µA was achieved from a single layer of NWs, which drove an electronic part without a battery. Zhu et al., grew ZnO NWs selectively on ITO coated silicon substrate followed by spin‐coating a layer of poly(methyl methacrylate) (PMMA) to cover them and then deposited aluminum electrodes.^[^ ^89^ ^]^ An extremely high output (58 V, 134 µA) was obtained by connecting 9 NGs in parallel. The enhanced output was attributed to the presence of PMMA preventing current leakage in the internal structure. Hu et al. assembled a PENG by dispersing conical‐shaped ZnO NWs onto a flat PMMA film.^[^ ^90^ ^]^ Upon mechanical deformation, the conical NWs generated macroscopic piezo potential along with its thickness. Under a compressive strain of 0.11% at a 3.67% s^–1^ strain rate, the PENG yielded an output of 2 V and 50 nA, which was sufficient to operate an liquid crystal display (LCD) screen.

Briscoe et al. fabricated a ZnO NR/poly(3,4‐ethylenedioxythiophene): poly(styrenesulfonate) (PEDOT: PSS) diode on an ITO coated polyethylene terephthalate (PET) substrate.^[^ ^91^, ^92^ ^]^ A p‐n junction was created between the p‐type polymer, PEDOT: PSS, and n‐type ZnO. The NRs had an aspect ratio of ≈20:1 and formed a dense array. The device had a low energy conversion efficiency (*η* = 0.0067%) at a maximum bending rate of 500 mm min^–1^. Later, Jalali et al. used a p‐type copper thiocyanate (CuSCN) as the passivating film on the surface of ZnO NRs and modified the device structure to improve the energy density of the NG by a factor of 10.^[^ ^93^ ^]^ The PENG produced a peak open‐circuit voltage (*V*_oc_) of 1.07 V with a corresponding power density of 434 µW cm^–2^ at a release acceleration of 50 g.

**Figure 6:**
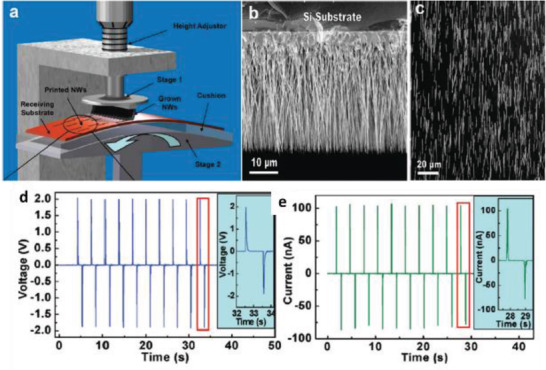
a) Experimental setup for transferring vertically grown ZnO NWs to a flexible substrate to make horizontally aligned ZnO NW arrays. b) SEM image of vertically aligned ZnO NWs grown on Si substrate by a physical vapor deposition method. c) SEM image of the as‐transferred horizontal ZnO NWs on a flexible substrate. d) *V*_oc_ and e) *I*_sc_ measured from the PENG at a strain of 0.1% and a strain rate of 5% s^–1^ with a deformation frequency of 0.33 Hz. The insets are an enlarged view of the boxed area for one cycle of deformation. Reproduced with permission.^[^ ^50^ ^]^ Copyright 2010, American Chemical Society.

Xu et al., synthesized ZnO NWs aligned parallel to the substrate using a lithographic masking technique involving a series of complicated steps.^[^ ^94^ ^]^ First, a seed layer was deposited by covering ZnO stripes partially with a chromium layer. This was followed by growing ZnO NW arrays via the solvent chemical method at 80 °C for 12 h. The PENG comprising of seven hundred rows of ZnO NWs, generated a peak *V*_oc_ of 1.26 V and *I*_sc_ of 26 nA at a 2.13% s^–1^ strain rate. This was much higher compared to a *V*_oc_ of 100 mV reported from a PENG oriented vertically in the same article. Later, Zhu et al. utilized a simple approach called “sweeping‐printing‐method,” in which vertically oriented ZnO NWs were relocated to a flexible substrate to form horizontally oriented arrays that were crystallographically aligned.^[^ ^50^ ^]^ The vertical ZnO NWs were grown on Si substrate and mounted on stage 1 as shown in **Figure** **6**a. They were then separated from the Si substrate and oriented on the host substrate due to the shear force applied by sweeping. The vertical NWs are shown in Figure 6b and the as‐transferred NWs are shown in Figure 6c. The flexible PENG produced a *V*_oc_ of 2.03 V, *I*_sc_ of 107 nA, and a corresponding power density of ≈11 mW cm^–3^ when bent (Figure 6d,e).

All the flexible devices described so far were fabricated on plastic substrates like PES, PET, Kapton film. Qin et al. presented a piezoelectric device with ZnO NWs grown radially on Kevlar fiber using a hydrothermal approach.^[^ ^95^ ^]^ A coating of tetraethoxysilane (TEOS) was used as a binding agent to attach the ZnO NWs to the fiber surface and each other. The ZnO NWs were single crystalline with diameters ranging between 50–200 nm and characteristic lengths of 3.5 µm. Two fibers, one with 300 nm Au coating and the other as grown were entangled and electricity was generated by brushing the NWs with respect to each other. The textile fiber‐based NG was able to produce a *V*_oc_ of 1 mV and a very low current of 5 pA due to a large loss in the fiber because of immensely high inner resistance. Similarly, Bai et al., developed a woven NG using two types of fibers—one with ZnO NWs and the other with Pd coated ZnO NWs.^[^ ^96^ ^]^ However, this also yielded a poor short circuit current of 17 pA due to the same reason mentioned above.

**Figure 7:**
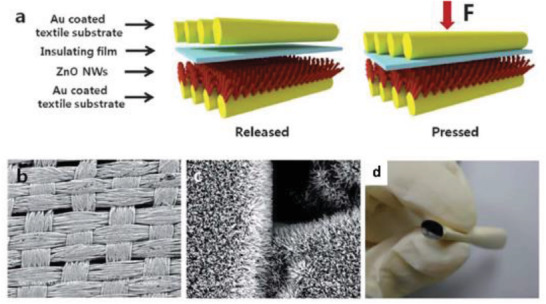
a) Schematic diagram of the textile‐based hybrid NG. b,c) Large‐area SEM images of ZnO NWs grown on textile substrate. d) Photographic image of textile substrate post rolling. Adapted with permission.^[^ ^98^ ^]^ Copyright 2012, The Royal Society of Chemistry.

Eventually, Qiu et al. proposed an ultra‐high flexible NG by growing ZnO NRs on a paper substrate.^[^ ^97^ ^]^ A small output of 10 mV and 10 nA was generated by the NG and it was demonstrated that electric output could be improved by increasing the device size. Similar work on paper substrates was reported by Lei et al., afterward.^[^ ^28^ ^]^ Kim et al. illustrated a hybrid NG on a woven textile substrate by the integration of a dielectric layer and ZnO NWs in between textile substrates as shown in **Figure** **7**a.^[^ ^98^ ^]^ The ZnO NWs were uniformly grown on the textile substrate as shown by SEM images in Figure 7b,c. Figure 7d demonstrates excellent flexibility of the rolled textile substrate. The textile‐based NG yielded an improved output voltage of 8 V and a current of 2.5 µA by utilizing sonic waves of 100 dB at 100 Hz as input. The higher output was ascribed to the synergistic piezoelectric effect of ZnO NWs and electrostatic effect of a dielectric film on the textile substrate.

Tamvakos et al., grew large arrays of ZnO NRs with high aspect ratio and excellent crystallinity by template‐free electrochemical deposition approach.^[^ ^99^ ^]^ The mean value of the d_33_ coefficient measured over many individual NRs was 11.8 pC N^–1^, which is ≈18% higher than 9.93 pC N^–1^ measured for ZnO bulk material.^[^ ^100^, ^101^, ^102^ ^]^ The d_33_ value was higher by a factor ranging between 28–167%, compared to ZnO nanostructures synthesized by the aqueous chemical method,^[^ ^103^ ^]^ hydrothermal,^[^ ^104^ ^]^ and template‐assisted vapor deposition.^[^ ^105^ ^]^

The literature suggests that the hydrothermal process using an aqueous solution of Zn(NO_3_)_2_.6H_2_O and HMTA has been most commonly used for synthesizing ZnO NWs. The other synthesis methods included thermal vapor deposition, physical vapor deposition, and template‐free electrochemical deposition. The length of ZnO NWs was in the range of 1–500 µm and their diameter between 50–300 nm. Poling treatment was not done in any of the research reported. The highest output voltage of 58 V and current of 134 µA was obtained by connecting nine PENGs in parallel upon punching by human palm.^[^ ^89^ ^]^

### 3.2 2D ZnO Nanostructures

This section briefly discusses the piezoelectric properties and energy harvesting capabilities of 2D ZnO nanostructures such as nanosheets and nanowalls, combined with reported synthesis techniques along with PENG fabrication methods.

**Figure 8:**
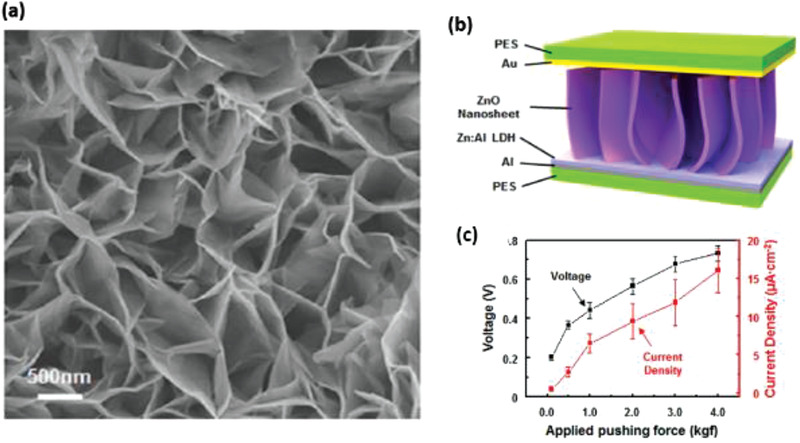
a) FE‐SEM image of the ZnO nanosheets network grown on Al. b) Schematic image of 2D ZnO nanosheet‐based NG. c) Output voltage and current density of the NG obtained by varying the applied pushing force. Reproduced with permission.^[^ ^106^ ^]^ Copyright 2013, Springer Nature.

Kim et al., illustrated the use of 2D ZnO nanosheets and an anionic nanoclay layer on an aluminum (Al) electrode to generate piezoelectric power.^[^ ^106^ ^]^ The ZnO nanosheet/anionic layer network was synthesized via an aqueous solution of zinc nitrate—HMT at 95 °C and their surface morphology was uneven as shown in **Figure** **8**a. The PENG was constructed by using gold plated PES as a top electrode, Al coated PES as bottom electrode and sandwiching the ZnO nanosheet network/anionic nanoclay heterojunction between them. Figure 8b displays a schematic of the PENG; the layered double hydroxide acted as an anionic nanoclay in this work. The voltage and current density obtained from the PENG was ≈0.7 V and 17 µA cm^–2^ when a compressive force of 4 kgf was applied (Figure 8c). It was proposed that the combined effect of deformation behavior in ZnO nanosheets, coupled semiconducting and piezoelectric properties of ZnO and self‐formation of anionic nanoclay layer was responsible for power generation. Gupta et al. synthesized ZnO nanosheets by doping ZnO NRs with vanadium for application in DC powered PENG.^[^ ^107^ ^]^ It generated an output *j* ≈ 1.0 µA cm^–2^ when the same value of compressive force was applied.

The synthesis of ZnO nanowall and nanowall‐nanowire hybrid structure on a graphene substrate was reported by Kumar et al. using CVD at 900 °C by precisely controlling the thickness of Au catalyst.^[^ ^108^ ^]^ Despite the higher sheet resistance of graphene (400 Ω) compared to ITO (60 Ω),^[^ ^85^, ^86^ ^]^ the hybrid nanowall‐nanowire NG generated a DC output voltage of 20 mV and *j* ≈ 500 nA cm^–2^ on applying 0.5 kgf compression force. Saravanakumar and Kim assembled a PENG consisting of a ZnO nanowall structure on two surfaces of the PMMA coated flexible substrate.^[^ ^109^ ^]^ The nanowall had a wall thickness of 60–80 nm, length of about 2–3 µm and showed a higher response to UV light due to vacancies being present in the nanowall structure. The PENG produced a maximum of 2.5 V output voltage and 80 nA current when deformed by a human finger.

Fortunato et al. compared the piezoelectric properties of ZnO‐NRs vertically grown over ITO substrate and ZnO nanowalls over the aluminum substrate.^[^ ^110^ ^]^ Both nanostructures were synthesized via chemical bath deposition. The obtained d_33_ values were 7.01 ± 0.33 pC N^–1^ for ZnO‐NRs and 2.63 ± 0.49 pC N^–1^ for ZnO nanowall films, indicating better piezoelectric properties of NRs compared to nanowalls. This was due to better orientation along the c‐axis and a lower defect rate of ZnO NRs compared to nanowalls.

From the discussed literature in this section, it can be inferred that 2D ZnO nanostructures exhibit lower piezoelectric outputs compared to 1D ZnO nanostructures. A maximum output of 2.5 V and 80 nA was generated by a PENG built using ZnO nanowalls when folded by a human finger.^[^ ^109^ ^]^ The synthesis methods of these 2D nanostructures were hydrothermal process using an aqueous solution of Zn(NO_3_)_2_.6H_2_O and HMTA and CVD without any poling treatment.

### 3.3 Nanostructures of Other Piezoelectric Materials

This section focuses on other nanostructured piezoelectric materials like PZT and PMN‐PT. Analogous to Sections 3.1 and 3.2, we review their synthesis methods, piezoelectric properties, fabrication techniques of PENGs along with their energy harvesting performance.

**Figure 9:**
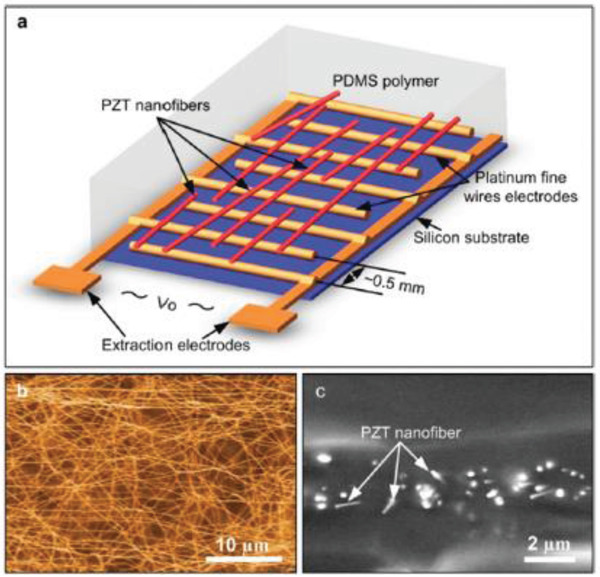
a) Schematic of the PZT nanofiber NG, b) SEM image of the electrospun PZT nanofibers. c) Cross‐sectional SEM image of the PZT nanofibers embedded in PDMS matrix. Reproduced with permission.^[^ ^118^ ^]^ Copyright 2010, American Chemical Society.

Lead zirconate titanate (PbZr_0.5_Ti_0.5_O_3_), commonly known as PZT is a well‐known piezoelectric material due to its high d_33_ coefficient of 500–600 pC N^–1^,^[^ ^111^, ^112^, ^113^, ^114^, ^115^ ^]^ which helps in generating much higher outputs compared to ZnO based materials. PZT nanofibers synthesized by electrospinning display high flexibility, mechanical strength and piezoelectric voltage constant (g_33_ = 0.079 Vm N^–1^)^[^ ^116^ ^]^ compared to the bulk, thin films or microfibers. In the electrospinning process, high voltage is applied to generate an electrically charged jet of precursor solution, which is squeezed through a small diameter needle and deposited on a collecting plate.^[^ ^117^ ^]^ Chen et al. electrospun PZT nanofibers of ≈500 µm length and 60 nm diameter by blending polyvinyl pyrrolidone (PVP) in the PZT precursor solution (**Figure** **9**b).^[^ ^118^ ^]^ Subsequently, PVP was removed by annealing the PZT/PVP fibers at 650 °C to obtain a pure perovskite phase of PZT. The PENG was integrated by depositing electrospun PZT nanofibers on interdigitated platinum electrodes set up on a silicon substrate and then enclosing it in soft PDMS matrix (Figure 9a,c). Then, poling was done by applying an electric field of 4 V µm^–1^ for 24 h at a temperature above 140 °C. The PENG was tested extensively across a wide range of loads and excitation frequencies. It generated a peak output voltage and power of 1.63 V and 0.03 µW, respectively, across a load resistance of 6 MΩ.

**Figure 10:**
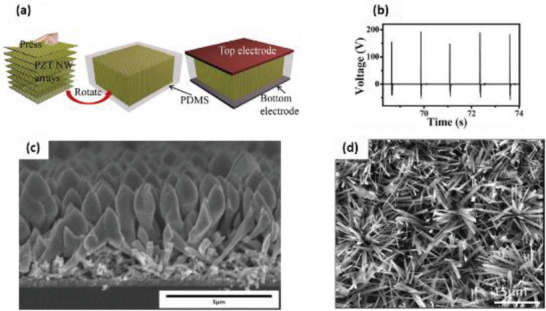
a) The fabrication process of the NG using oriented electrospun nanofibers. b) The output voltage of the NG under a periodic pressure of 0.53 MPa. a,b) Reproduced with permission.^[^ ^120^ ^]^ Copyright 2012, American Chemical Society. c) SEM image showing the hetero‐junction structure of ZnO NWs/PZT. Reproduced with permission.^[^ ^122^ ^]^ Copyright 2013, Elsevier B.V. d) SEM image of PMN‐PT nanowires. Reproduced with permission.^[^ ^123^ ^]^ Copyright 2012, American Chemical Society.

Electrospun PZT nanofiber arrays were produced by Wu et al. from PZT/PVP on multiple rows of parallel electrodes.^[^ ^119^ ^]^ The power density of the flexible NG was 200 µW cm^–3^ and it yielded a maximum *V*_oc_ of 6 V and *I*_sc_ of 45 nA when stretched. The generated power was sufficient to illuminate a LCD. Cui et al. placed electro spun PZT NWs onto magnetite (Fe_3_O_4_), connected them with silver electrodes and packaged them with PDMS to assemble a contactless NG.^[^ ^115^ ^]^ The NWs were deformed with the help of a magnet, generating a maximum current of 50 nA, the voltage of 3.2 V, corresponding to a power density of 170 µW cm^–3^. Gu et al., built an NG using arrays of electrospun PZT NWs cut from a film, rotating them by 90° and stacking several layers perpendicular to the substrate (**Figure** **10**a).^[^ ^120^ ^]^ A very high peak voltage of 209 V and *j* = 23.5 µA cm^–2^ was achieved under 0.53 MPa impact pressure (Figure 10b), much higher than previously reported values.^[^ ^89^ ^]^ Vertically aligned PZT NWs were also grown hydrothermally using polymer surfactants on titanium oxide^[^ ^121^ ^]^ and conductive Nb‐doped SrTiO_3_ substrates.^[^ ^51^ ^]^ The PZT NWs were grown epitaxially at 230 °C. A mono‐layer of PZT NWs were utilized to build the NG and it generated a peak *V*_oc_ of ≈0.7 V, *j* ≈ 4 µA cm^–2^ and power density of 2.8 mW cm^–3^ on applying impact force.^[^ ^51^ ^]^ No et al. synthesized ZnO NWs via hydrothermal process and then deposited PZT thin films on them by magnetron sputtering.^[^ ^122^ ^]^ The ZnO/PZT heterojunction structure (Figure 10c) was subjected to corona poling at 11 kV for 30 min. The NG revealed an improved current of 270 nA, compared to 0.5 nA from NG with only ZnO and 9 nA with the only PZT due to synergistic piezoelectric effects of both PZT and ZnO NWs.

Xu et al., synthesized 0.72Pb(Mg_1/3_Nb_2/3_)O_3_‐0.28PbTiO_3_ (PMN‐PT) nanowires via the hydrothermal method and reported an extremely high d_33_ value of 381 pCN^–1^ without poling.^[^ ^123^ ^]^ The morphology of PMN‐PT NWs consisted of wire‐like nanostructures with typical lengths between 200 and 800 nm as shown in Figure 10d. The width of these NWs was about 400 nm.

Manganese (Mn) doped (Na_0.5_K_0.5_)NbO_3_ (NKN) electrospun nanofibers were produced by Kang et al. utilizing acetic acid as a chelating agent at an annealing temperature of 750 °C.^[^ ^124^ ^]^ The 3 mol% Mn‐doped NKN nanofibers exhibited an enhanced d_33_ value of 40.06 pC N^–1^, which is five times greater than un‐doped nanofibers. The doped nanofibers were relocated to a PDMS coated PES substrate with interdigitated Pt electrodes to assemble the flexible PENG. Under bending strain, the PENG displayed an output performance of ≈0.3 V voltage and ≈50 nA current.

**Figure 11:**
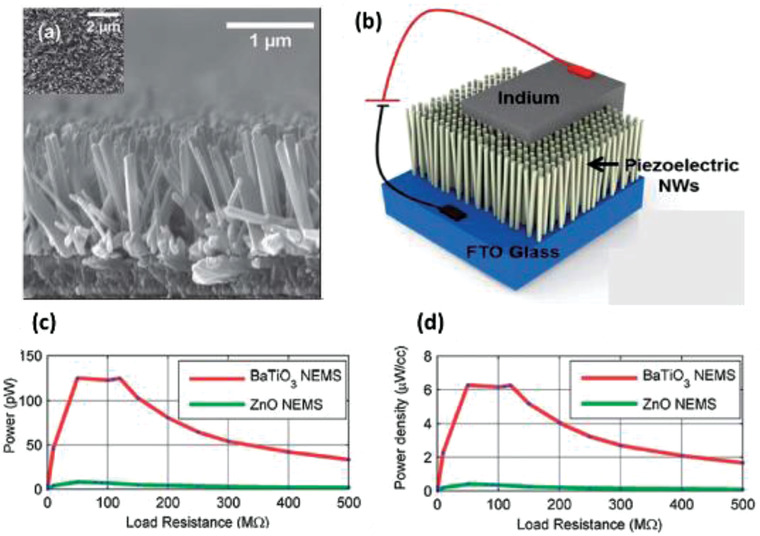
a) Cross‐sectional SEM image of the as‐synthesized BaTiO_3_ NW arrays with inset showing the top view. b) Schematic of the energy harvester constructed using BaTiO_3_ NW arrays. c) Power and d) power density of the BaTiO_3_ NW based PEH at various load resistances displaying a peak power of ≈125.5 pW and a peak power density of ≈6.27 µW cm^–3^ at an optimal resistance of 120 MΩ from 1 g acceleration. These peak power levels are much greater than the peak power from ZnO NW based PEH. Reproduced with permission.^[^ ^126^ ^]^ Copyright 2014, The Royal Society of Chemistry.

BaTiO_3_ nanostructures have also been commonly used for energy harvesting applications. The piezoelectric strain constant, d_33_, of BaTiO_3_ is ≈90 pC N^–1^.^[^ ^125^ ^]^ Koka et al., produced vertically aligned BaTiO_3_ NWs of ≈1 µm length via hydrothermal process on conductive fluorine‐doped tin oxide (FTO) substrate (**Figure** **11**a).^[^ ^126^ ^]^ The PEH was fabricated by using indium as the top electrode, FTO as the bottom electrode and BaTiO_3_ NW arrays were sandwiched between them as shown in Figure 11b. After that, it was poled by applying a high electric field of ≈120 kV cm^–1^ for 24 h. Peak power of ≈125.5 pW and power density of 6.27 µW cm^–3^ was generated by accelerating the PENG to 1 g at a load resistance of 120 MΩ (Figure 11c,d). This was nearly 16 times greater than ZnO NW based PENG reported in the same work. The enhanced performance was attributed to the larger electromechanical coupling coefficients of BaTiO_3_ in comparison to ZnO. Later on, the same group reported the growth of ultra‐long (up to 45 µm) BaTiO_3_ NWs on oxidized Ti substrate using a two‐step hydrothermal process.^[^ ^127^ ^]^ Sodium titanate NW arrays were first grown as precursors followed by converting them to BaTiO_3_ NW arrays as a result of their active ion exchanging property. The BaTiO_3_ NWs were placed on a conductive glass substrate using a Ti foil coated with PEDOT: PSS as the top electrode to construct an energy harvester. At 0.25 g acceleration, a large peak‐to‐peak voltage (Vp‐p) of 345 mV was obtained from the PENG due to the ultra‐long BaTiO_3_ NWs.

Liu et al., constructed a flexible PENG based on 0.93(Na_0.5_Bi_0.5_)TiO_3_‐0.07BaTiO_3_ (NBT‐0.07BT) nanofibers, synthesized by sol–gel electrospinning.^[^ ^128^ ^]^ The NBT‐0.07BT nanofibers have a perovskite structure with a d_33_ value up to ≈109 pC N^–1^ for a single NBT‐0.07BT nanofiber. When the dynamic load was applied using a human finger, the PENG produced a *V*_oc_ of ≈30 V and *I*_SC_ ≈ 80 nA, which powered a commercial LED.

Energy harvesting using gallium nitride (GaN) nanostructures has also been reported.^[^ ^129^, ^130^, ^131^ ^]^ Gogneau et al., studied the piezoelectric properties of GaN NWs synthesized via plasma‐assisted molecular beam epitaxy.^[^ ^130^ ^]^ When external deformation was applied to the NWs via AFM, Schottky contact was generated between the AFM‐tip and GaN NWs that was inversed with respect to the ZnO NW system.^[^ ^132^ ^]^ This showed that the piezoelectricity generation mechanism is dependent on the structural characteristics of the NWs. Jamond et al., synthesized a vertical array of GaN NWs that yielded a maximum voltage of 350 mV and a mean of 228 mV per NW, with a power density of ≈12.7 mW cm^–3^.^[^ ^131^ ^]^ The mechanism of energy generation in both cases was the same as proposed for ZnO NWs.^[^ ^73^, ^74^, ^132^, ^133^ ^]^

Based on discussed literature in this section, it can be summarized that the dominant synthesis methods for PZT, BaTiO_3_, PMN‐PT are electrospinning, hydrothermal processing and plasma‐assisted molecular beam epitaxy. Except for PMN‐PT NWs, poling was done on all materials. The highest d_33_ value of 500–600 pC N^–1^ was reported for PZT NWs.^[^ ^118^ ^]^ Also, the highest output voltage of 209 V, *j* = 23.5 µAcm^–2^ was obtained from PZT NWs based PENG under stress of 0.53 MPa.^[^ ^120^ ^]^ Table 1 summarizes the works on piezoelectric nanostructured materials for energy harvesting applications.

## 4 Piezoelectric Polymers

Polymers are carbon‐based materials that exhibit a piezoelectric effect because of their molecular structure and orientation. They are much softer than ceramics and display moderate values of strain coefficients (d_33_) and voltage coefficients (g_33_). However, their unique properties such as, design flexibility, low density, and simple processing make them appropriate for various energy harvesting applications. Polymers with semi‐crystalline structure have microscopic crystals randomly distributed within an amorphous bulk. These include PVDF,^[^ ^134^ ^]^ polyvinylidene fluoride‐trifluoro ethylene P(VDF‐TrFE),^[^ ^135^ ^]^ liquid crystal polymers,^[^ ^136^ ^]^ polyamides,^[^ ^137^ ^]^ cellulose and its derivatives,^[^ ^138^, ^163^, ^166^ ^]^ parylene C,^[^ ^60^ ^]^ PLA,^[^ ^139^ ^]^ etc. Piezoelectricity is observed in amorphous or non‐crystalline polymers when its molecular structure contains dipoles. The dipoles are aligned by poling at temperatures higher than the polymer's glass transition temperature (*T*_g_). Such polymers consist of polyimide,^[^ ^61^, ^140^ ^]^ nylons,^[^ ^141^ ^]^ polyurea,^[^ ^142^ ^]^ polyurethanes,^[^ ^139^ ^]^ etc. Table 2 compares the piezoelectric properties of these polymers. Fukuda^[^ ^139^ ^]^ and Ramadan et al.^[^ ^31^ ^]^ wrote detailed reviews on the piezoelectric properties of these polymers.

### 4.1 Polyvinylidene Fluoride Homopolymer

**Figure 12:**
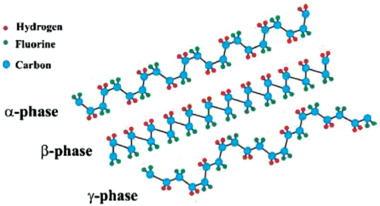
Schematic representation of the chain conformation for the *α*, *β*, and *γ* phases of PVDF. Reproduced with permission.^[^ ^150^ ^]^ Copyright 2014, Elsevier Ltd.

PVDF is a semi‐crystalline, thermoplastic polymer synthesized by the polymerization of vinylidene difluoride (VDF).^[^ ^170^, ^171^, ^172^, ^173^ ^]^ Kawai discovered piezoelectricity in PVDF in 1969.^[^ ^141^ ^]^ PVDF consists of 3 wt% hydrogens, 59.4 wt% fluorine and exhibits higher piezoelectric coefficients compared to other polymers.^[^ ^154^, ^174^ ^]^ It has 50–70% crystallinity and displays five polymorphs: *α*, *β*, *γ*, *δ*, and ɛ, although *α*, *β*, and *γ* are seen more frequently.^[^ ^154^ ^]^ The larger van der Waals radius of fluorine atoms (1.35 Å) compared to hydrogen atoms (1.2 Å) is the reason behind the existence of polymorphism in PVDF.^[^ ^154^ ^]^ The *α* phase (form II) has a trans‐gauche configuration, in which the polymeric chains are in nonpolar conformation (TGTG′), with alternating H_2_ and F_2_ atoms on two sides of the chain (**Figure** **12**).^[^ ^154^ ^]^ The polar *β* phase (form I), has zigzag all‐trans conformation (TTT) of polymeric chains and possesses the maximum dipolar moment per unit cell (8 × 10^–30^ C m) in comparison to other phases.^[^ ^175^ ^]^ The polar *γ* phase (form III) and *δ* phase have TTTGTTTG′ and TGTG′ conformations, respectively, that enable PVDF to exhibit piezoelectric properties along with the *β* phase.^[^ ^134^, ^176^ ^]^ An exhaustive review has been written by Martins et al. on various phases present in PVDF/PVDF copolymers.^[^ ^150^ ^]^

Fourier transform infrared spectroscopy (FTIR) results have been commonly utilized to evaluate the content of electroactive phases in PVDF. In a sample consisting of *α* and *β*‐PVDF, the relative fraction of *β*‐phase, [F(*β*)], is given by Beer–Lambert law (Equation (8)).^[^ ^177^ ^]^

(8) $$F\;\;\left(\beta \right)=\frac{A_{\beta }}{\left(\frac{K_{\beta }}{K_{\alpha }}\right)A_{\alpha }+\;A_{\beta }}\;\times \;100\%\;$$

where *Α_*α*_* and *Α_*β*_* are the absorbances of *α* and *β* phases at 766 and 840 cm^–1^; *K_*α*_* and *K_*β*_* are the absorption coefficients at the respective wavenumbers with values of 6.1 × 10^4^ and 7.7 × 10^4 ^cm^2^ mol^–1^.^[^ ^150^ ^]^

The polar *γ* phase content in a material containing *α* and *γ* phases is given by Equation (9):

(9) $$F\;\;\left(\gamma \right)=\frac{A_{\gamma }}{\left(\frac{K_{\gamma }}{K_{\alpha }}\right)A_{\alpha }+\;A_{\gamma }}\;\times \;100\%\;$$

where *Α_*α*_* and *Α_*γ*_* are the absorbances of *α* and *γ* phases at 762 and 845 cm^–1^; *K_*α*_* and *K_*γ*_* are the absorption coefficients at the respective wavenumbers with values of 0.365 and 0.150 µm^–1^.^[^ ^178^ ^]^

Among all phases, the *β*‐phase particularly exhibits exceptional ferroelectric, piezoelectric and pyroelectric properties.^[^ ^179^, ^180^, ^181^ ^]^ *β*‐PVDF is usually formed by stretching of the *α* phase,^[^ ^180^, ^182^, ^183^ ^]^ melt crystallization under high pressure,^[^ ^184^, ^185^, ^186^ ^]^ external electric field,^[^ ^187^, ^188^, ^189^, ^190^, ^191^, ^192^ ^]^ and fast cooling rates;^[^ ^193^ ^]^ from solvent casting,^[^ ^194^, ^195^ ^]^ electrospinning,^[^ ^196^, ^197^ ^]^ and by the addition of different nucleating agents (described in Section 5).

During stretching, the applied stress aligns the PVDF chains, which induces *β* phase formation. A maximum d_31_ value of 60 pC N^–1^ was reported under a poling field of 0.55 MV cm^–1^, at a temperature of 80 °C and stretching ratio, *R*, of 4.5 by Kaura et al.^[^ ^145^ ^]^ In a similar study done by Salimi and Yousefi, 74% *β*‐phase was achieved by stretching at 90 °C, for *R* between 4.5 and 5.^[^ ^180^ ^]^ The largest amount of *β*‐phase and d_33_ value of 34 pC N^–1^ for *R* = 5 at 80 °C was obtained by Gomes et al.^[^ ^143^ ^]^ Panigrahi et al., showed a higher piezoelectric response with a poled PVDF device compared to an unpoled one. The PVDF film was prepared via solution casting and the d_33_ value was reported as −5 pC N^–1^ after poling at an electric field of 20 kV cm^–1^.^[^ ^198^ ^]^

**Figure 13:**
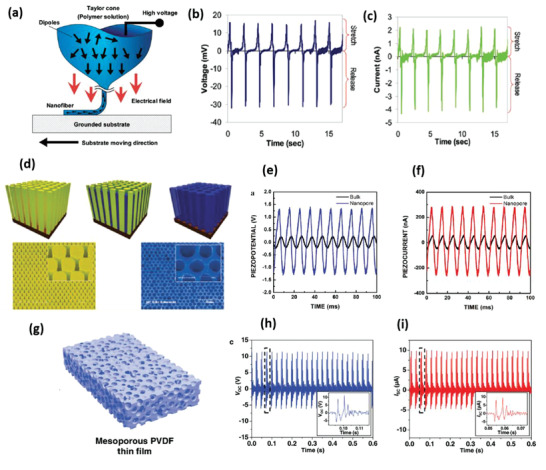
a) Near‐field electrospinning (NFES) to create PVDF nanofibers onto a substrate. b) Output voltage and c) current measured with respect to time under applied strain at 2 Hz. a‐c) Reproduced with permission.^[^ ^201^ ^]^ Copyright 2010, American Chemical Society. d) Schematic depiction of PVDF nano porous arrays grown by the template‐assisted method. e) Piezoelectric potential and f) current obtained from porous PVDF and bulk films under the same force. d‐f) Reproduced with permission.^[^ ^208^ ^]^ Copyright 2011, American Chemical Society. g) Schematic diagram of mesoporous PVDF film. h) The voltage and i) current output of the PENG under perpetual surface oscillation. Insets show the output curve in the course of one oscillation cycle. g‐i) Reproduced with permission.^[^ ^209^ ^]^ Copyright 2014, Wiley‐VCH.

Chang et al. used near field electrospinning (NFES)^[^ ^199^, ^200^ ^]^ in combination with in situ stretching and poling to direct‐write PVDF nanofibers as shown in **Figure** **13**a.^[^ ^201^ ^]^ The stretching forces and high electric fields (>10^7^ V m^–1^) from electrospinning aligned the dipoles in the nanofibers which facilitated *α*‐*β* phase transformation. The reported d_33_ value was −63.25 pC N^–1^ for single fiber^[^ ^202^ ^]^ and −57.6 pC N^–1^ for PVDF fiber mats,^[^ ^203^ ^]^ which is about four times of PVDF films (≈15 pC N^–1^). The outputs were in the range of 5–30 mV and 0.5–3 nA under stretching and releasing of more than 50 NGs (Figure 13b,c). A peak voltage of 0.2 mV and current of 35 nA was obtained from 500 nanofibers connected in parallel, under repeated mechanical straining.^[^ ^204^ ^]^ Liu et al. used a hollow cylindrical NFES process to produce oriented PVDF fibers with a high *β* phase.^[^ ^205^ ^]^ A maximum voltage and current of 76 mV and 39 nA were produced on continuous stretching and releasing the nanofibers at 0.05% strain at 7 Hz frequency. Pan et al. used the same process to fabricate PVDF hollow fibers and obtained a voltage and power of 71.66 mV and 856.07 pW respectively, which was higher than solid PVDF fibers (45.66 mV, 347.61 pW) due to higher elongation and Young's modulus.^[^ ^206^ ^]^ Kanik et al. synthesized kilometer long, micro‐and nanoribbons of PVDF based on thermal fiber drawing without electrical poling.^[^ ^207^ ^]^ The polar *γ* phase was obtained due to the combined effect of high temperature and stress applied during the thermal drawing process. The effective d_33_ value measured from an individual 80 nm thick nanoribbon was −58.5 pC N^–1^. For energy harvesting and as tapping sensor, two devices were built that displayed a peak voltage of 60 V and a current of 10 µA.

Cha et al. fabricated nanoporous arrays of PVDF using a template‐assisted method via strain confinement approach as shown in Figure 13d.^[^ ^208^ ^]^ Sound waves of 100 dB at 100 Hz were used as the input source for energy harvesting. The PENG exhibited an enhanced *V*_oc_/*I*_sc_ of 2.6 V/0.6 µA compared to the film system (0.5 V/0.1 µA) on applying identical force (Figure 13e,f). The porous PVDF nanostructure showed a d_33_ value −32.5 pC N^–1^, which is the same as bulk PVDF. A PENG was designed by Mao et al. using spongy mesoporous PVDF films, with an average pore size of 35–45 nm, as shown in Figure 13g.^[^ ^209^ ^]^ The peak *V*_oc_ increased from 3.5 to 11.0 V with porosity increase from 6.5% to 32.6% as a result of the increase in *β* phase amount from 10% to 50% and sponge‐like properties of the porous films. The PENG generated a peak *V*_oc_ of 11 V and *I*_sc_ of 9.8 µA (Figure 13h,i), which is about two times higher than peak *V*_oc_ of 3.7–5.3 V obtained from solid PVDF film. Chen et al. conducted a similar study on mesoporous PVDF films, showing an improved output performance of over 100% in comparison to solid PVDF films.^[^ ^210^ ^]^

Chen et al. showed direct *β* phase formation in PVDF ultra‐thin films with the alignment of dipoles perpendicular to the substrate using the Langmuir–Blodgett (LB) deposition process.^[^ ^211^ ^]^ The LB film had a d_33_ coefficient of 49.4 pC N^–1^ compared to 20.7 pC N^–1^ for the spin‐coated film of 400 nm thickness. Hydrogen bond formation between PVDF and water molecules via LB deposition was the mechanism of *β* phase formation in PVDF.

Jin et al. developed *β*‐PVDF based PENG as an acceleration sensor with high sensitivity (2.405 nA s^2^ m^–1^) and stability (97% output current after 10 000 cycles at 4 Hz).^[^ ^212^ ^]^ The PENG's outstanding performance (*I*_sc_ up to 145 nA cm^–2^) was due to melt crystallization of PVDF at high temperature and pressure, resulting in high *β*‐phase content of 86.48%. The self‐powered sensor was integrated with three PENGs for acceleration measurement along the three axes and vehicle safety monitoring system. Zhang et al. reported an energy harvester for scavenging wind energy from PVDF beams.^[^ ^213^ ^]^ A maximum voltage of 160.2 V and power of 2566.4 µW was generated at a wind speed of 14 ms^–1^. Bera and Sarkar prepared a PVDF based NG for harvesting energy from renewable sources like ocean waves, rain, and wind.^[^ ^214^ ^]^ The output power was primarily dependent on the crystallinity and content of *β* phase‐PVDF. The highest *β*‐phase amount of 97% and output voltage of 2.3 V has been reported from a textile‐based energy harvester, made from PVDF fibers by melt spinning.^[^ ^215^ ^]^ Similarly, a voltage of 2.2 V was obtained from PVDF fibers tested under the application of 1.02 kgf impact force by Hadimani et al.^[^ ^49^ ^]^ These fibers were produced by melt extrusion and in situ poling at a temperature of 80 °C and an extension ratio of 4:1. In the following section, we will discuss the piezoelectric properties of PVDF‐based copolymers that are extensively used as well.

### 4.2 Polyvinylidene Fluoride‐Trifluoro Ethylene Copolymers

**Figure 14:**
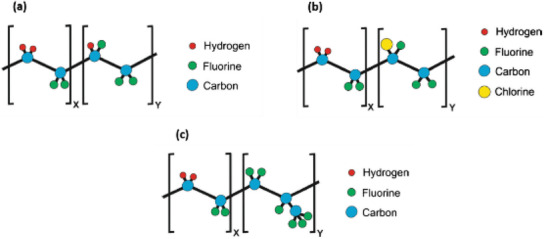
Schematic representation of the a) P(VDF‐TrFE), b) P(VDF‐HFP), and c) P(VDF‐CTFE) repeat units. Reproduced with permission.^[^ ^150^ ^]^ Copyright 2014, Elsevier Ltd.

P(VDF‐TrFE) is a semi‐crystalline, thermoplastic copolymer in which VDF can copolymerize in any amount with trifluoroethylene (TrFE)^[^ ^216^, ^217^, ^218^, ^219^ ^]^ (**Figure** **14**a). The incorporation of TrFE into PVDF in any amount induces *α* to *β* phase transition and change in conformation from trans‐gauche to all‐trans.^[^ ^220^, ^221^ ^]^ The piezoelectric properties in P(VDF‐TrFE) arises below its Curie temperature due to the orientation of the crystalline phase under a strong poling field. It exhibits high piezoelectric coefficients (d_33_ ≈ −30 to −40 pC N^–1^ and d_31_ ≈ 25 pC N^–1^)^[^ ^148^, ^151^ and has been explored in several energy harvesting applications.^[^ ^222^ ^]^

Pi et al. reported a *V*_oc_ of 7 V, *I*_sc_ of 58 nA, and *j* = 0.56 µA cm^–2^ from a PENG using spin‐coated P(VDF‐TrFE) (75/25 mol%) film.^[^ ^223^ ^]^ The impact of strain rate variation on the electrical output of the PENG was studied and an analytical model was established that correlated well to experimental results. Bae and Chang. showed that a consecutive treatment of “annealing and cooling‐pressing‐poling” resulted in a maximum F(*β*) of 66.33% and voltage of 1.10 V for P(VDF‐TrFE) films.^[^ ^48^ ^]^ Similar work was done by Oliveira et al. showing an increase in crystallinity by 19% after annealing at 130 °C for 48 h.^[^ ^224^ ^]^ The transition from *α* to *β* phase happened after annealing, leading to an F(*β*) of 94% and an increase in d_33_ value from 13 to 18 pC N^–1^.

Lee et al. used micropatterning to create pyramid‐shaped and trigonal line‐shaped morphologies on P(VDF‐TrFE).^[^ ^225^ ^]^ The micropatterned P(VDF‐TrFE) based PENG generated higher output (4.4 V and 3.4 µA) than the flat film PENG (1 V and 0.1 µA) under vertical compressive force. The enhanced output was due to higher strain developed in micropatterned films under the same force. Cauda et al. prepared nanowires of PVDF and P(VDF‐TrFE) (70/30) by template‐wetting into 200 nm‐sized channels of anodic porous alumina (APA) membranes.^[^ ^226^ ^]^ It was shown that nanoconfinement led to the crystallization of *β* phase in both polymers; the reference PVDF thin film crystallized in the nonpolar *α* phase. The maximum d_33_ values for P(VDF‐TrFE) and PVDF templated nanowires were −8.2 and −6.5 pC N^–1^. Non‐poled PVDF film possessed a d_33_ value of −15 pC N^–1^, whereas poled and bulk films of P(VDF‐TrFE) and PVDF possessed d_33_ values between −20 and −30 pC N^–1^. Similarly, Lutkenhaus et al. reported a significant change in crystallization behavior of P(VDF‐TrFE) when confined within APA template with pores <40 nm diameter.^[^ ^149^ ^]^ Crystallization of *β*‐phase was enhanced and *α* phase formation was suppressed. This suggests that nano‐confinement increases the piezoelectric response of P(VDF‐TrFE) because of the oriented crystallization of ferroelectric domains. Bhavanasi et al. showed enhanced piezoelectric properties of P(VDF‐TrFE) nanotubes prepared with nanoconfinement effect.^[^ ^227^ ^]^ The poled 200 nm nanotubes at 40% reduced poling field showed a higher d_33_ value of 44 pC N^–1^ compared to 20 pC N^–1^ for fully poled films. The P(VDF‐TrFE) nanotubes displayed superior output (voltage ≈ 4.8 V, power ≈ 2.2 µW cm^−2^) compared to bulk films, under application of 0.075 MPa compressive pressure at 1 Hz frequency. Chen et al. fabricated P(VDF‐TrFE) (75/25) films with tunable micro‐porous structures that displayed a maximum d_33_ value of 25.4 pC N^–1^.^[^ ^228^ ^]^ The porous structures increased power generation by five‐fold as compared to solid films under 0.5 N periodic bending force. Aliane et al. showed that in case of P(VDF‐TrFE) composition of 72.2/27.8 mol%, a higher *β* phase was obtained for a shorter polymer chain length with a larger grain size.^[^ ^229^ ^]^ Moreover, the d_33_ value increased to −58 pC N^–1^ by reducing the molecular weight of P(VDF‐TrFE) to 470 kg mol^–1^. This study proved that TrFE composition and the crystallization of P(VDF‐TrFE) exerts a strong influence on piezoelectric properties.

P(VDF‐TrFE) fiber nanogenerators have also shown promise. A maximum output voltage of ≈1 V and power of 0.6 nW was generated from electrospun P(VDF‐TrFE) (70/30) nanofiber‐based PEH.^[^ ^230^ ^]^ Persano et al. demonstrated ultra‐high sensitivity for measuring pressures as low as 0.1 Pa using P(VDF‐TrFE) (75/25 wt%) nanofibers. The PENG exhibited a voltage of 1.5 V and currents up to 40 nA under bending conditions.^[^ ^231^ ^]^

### 4.3 Polyvinylidene Fluoride‐Chlorotrifluoroethylene Copolymers

The incorporation of chlorotrifluoroethylene (CTFE) into PVDF chains formulates P(VDF‐CTFE) with a chemical structure [(CH_2_CF_2_)*x*(CF_2_CFCl)*y*]*n*) ^[^ ^153^ ^, [^ ^154^ ^]^ (Figure 14b). P(VDF‐CTFE) copolymer with little amounts of VDF is semi‐crystalline, while those containing 25–70 mol% VDF are amorphous.^[^ ^232^ ^]^ At higher VDF amounts of greater than 70% thermoplastic copolymers are obtained, known as flexible PVDF.^[^ ^233^ ^]^ This copolymer exhibits a higher strain response than PVDF but requires a higher electric field. At 12 mol% CTFE content, the copolymer exhibits an electrostrictive strain response of more than 5% and d_33_ value of 140 pC N^–1^ when poled at an electric field of 70 MV m^–1^.^[^ ^152^ ^]^

### 4.4 Polyvinylidene Fluoride‐Hexafluoropropylene Copolymers

The introduction of hexafluoropropylene (HFP) into PVDF leads to the formation of P(VDF‐HFP) copolymer with various properties depending on the content of HFP (Figure 14c). The copolymer exhibits thermoplastic properties at low HFP contents of 15–19 mol% and elastomeric properties at higher HFP content (greater than 19–20 mol%).^[^ ^234^, ^235^, ^236^, ^237^ ^]^ A high d_31_ value of 30 pC N^–1^ has been reported for P(VDF‐HFP) uniaxially drawn films.^[^ ^156^ ^]^ Another study reported a d_31_ value of 43.1 pC N^–1^ and k_31_ of 0.187 for 10 wt% P(VDF‐HFP).^[^ ^157^ ^]^ P(VDF‐HFP) films produced via extrusion, followed by simultaneous stretching and poling showed a maximum d_33_ value of 24 pC N^–1^.^[^ ^155^ ^]^

### 4.5 Other Piezoelectric Polymers

#### 4.5.1 Polylactic Acid

PLA is emerging as a popular option in energy harvesting due to its superior piezoelectric properties. In PLA, the carbonyl groups induce polarity and it exhibits a d_14_ value of 10 pC N^–1^ in the absence of poling.^161^ ^],[^ ^238^ ^]^ For the first time, Zhao et al. used a poly‐*L*‐lactic acid (PLLA) based cantilever device for energy harvesting applications.^[^ ^239^ ^]^ A maximum power of 14.17 µW and *V*_oc_ of 9.05 V was generated by the device which was used to charge a 3.2 V battery. The PLLA film showed a d_14_ value of 9.57 pC N^–1^ after poling at 140 °C for 24 h. Zhu et al. synthesized electrospun PLLA nanofibers for strain sensing and energy harvesting via human joint motion.^[^ ^240^ ^]^ The nanofibers displayed a *V*_oc_ and *I*_sc_ of 0.55 V and 230 pA, respectively, at a strain angle of 28.9° and peak power of 19.5 nW from joint motion. Similarly, Lee et al. built a piezoelectric sensor using electrospun PLLA nanofiber webs that were used to operate LEDs.^[^ ^241^ ^]^

#### 4.5.2 Polyureas

Polyureas is a type of elastomer produced from di‐isocyanates and diamines, that exhibits piezoelectric and pyroelectric properties after poling. The first piezoelectric polyurea, namely, aromatic polyurea exhibited a d_31_ value of 10 pC N^–1^ with stability up to 200 °C.^169^ ^], [^ ^238^ ^]^ A d_3l_ value of 1.7 pC N^–1^ was reported for poled polyurea‐9.^[^ ^142^ ^]^ When an odd number of methyl groups are present, aliphatic polyureas display ferroelectric hysteresis while being piezoelectric. Their piezoelectric coefficients are lower than aromatic polyureas and are heavily dependent on poling temperature (70–150 °C).^[^ ^136^ ^]^ Koyama et al. reported power generation using a polyurea thin film attached to a cantilever.^[^ ^242^ ^]^ At a load resistance of 1 MΩ, optimum output was obtained at an efficiency of 0.233% using a cantilever of length 4 mm. Hattori et al. prepared aliphatic polyurea‐5 films by vapor deposition and reported piezoelectric stress constant (e_31_) of 10 mC m^–2^ and d_31_ = 4 pC N^–1^.^[^ ^243^ ^]^

#### 4.5.3 Polyurethanes

Polyurethanes are a class of alternating copolymers that are usually formed by reacting a di‐ or tri‐isocyanate with a polyol^167^, ^168^. In 1999, Fukada et al. reported d_31_ of 38 pC N^–1^ and k_31_ of 0.14 in isotropic polyurethane films post poling at a DC field of 5 MV m^–1^.^[^ ^244^ ^]^ The d_31_ value was nearly twice and k_31_ was similar to that of poled PVDF film. Later in 2016, Moody et al. showed high d_33_ coefficients up to 244 ± 30 pC N^–1^ on polyurethane foams by adding polar dopant molecule 2‐chloro‐4‐nitroaniline into the polymer matrix.^[^ ^245^ ^]^ Under the application of force, the polymer foams experienced substantial volume changes and the dipole‐doped polymers increased piezo responsiveness multifold in comparison to conventional polymers and ceramics.

#### 4.5.4 Polyamides

Polyamides or nylons are a class of semi‐crystalline polymers that exhibit a low degree of piezoelectricity. A d_31_ value of 0.5 pC N^–1^ was reported for the first time by Kawai for a stretched, poled film of Nylon‐11.^[^ ^141^ ^]^ The odd‐numbered polyamides such as Nylon‐5, Nylon‐11 show piezoelectric and pyroelectric properties as a result of their polar structure. This is because, in odd polyamides, a 3.7D dipole moment exists between the even‐numbered methylene group and amide group resulting in a net dipole moment, whereas in even polyamides the amide dipole counterbalances each other.^[^ ^238^ ^]^ Takase et al. reported d_31_ = 14 pC N^–1^, k_31_ = 0.054 for Nylon‐11, and d_31_ = 17 pC N^–1^, k_31_ = 0.049 for Nylon‐7 in the temperature range of 100–200 °C.^[^ ^160^ ^]^

#### 4.5.5 Polyacrylonitrile, Polyimides, Polypropylene

Apart from the polymers listed in previous sections, polymers such as PAN, polyimides (PI), and polypropylene (PP) also display piezoelectric properties and can be utilized for energy harvesting applications. Recently, Wang et al. reported surprisingly high piezoelectric properties in electrospun PAN nanofibers, which was attributed to the high amount of planar Sawtooth PAN conformation within nanofibers.^[^ ^246^ ^]^ When subjected to compressive stress, a small piece of PAN nanofibers (5 cm^2^) produced up to 2.0 V, which was higher than PVDF nanofibers at the same conditions. Cai et al. showed that voltage output from PAN thin films was improved by controlling the poling temperature.^[^ ^247^ ^]^ The PENG generated the highest output of ≈0.9 V when poling temperature was 80 °C.

Polyimides (PI) are a class of amorphous polymers that are suitable for piezoelectric applications because of their high *T*_g_ value.^[^ ^136^ ^]^ Their properties were studied extensively by Harrison and Ounaies.^[^ ^136^ ^]^ Gonzalo et al. synthesized PI for high‐temperature applications and showed that piezoelectric coefficients are dependent on the quantity of nitrile dipoles present in the PI backbone in addition to the imidization process and poling parameters.^[^ ^248^ ^]^

Polypropylene (PP) is a thermoplastic polymer synthesized via chain‐growth polymerization from the monomer propylene. Wu et al., in 2015, fabricated a cellular PP based flexible PENG with a peak power density of ≈52.8 mW m^–2^ that was used for energy harvesting and self‐powered health monitoring systems.^[^ ^249^ ^]^ The multilayered PP film exhibited a d_33_ value of ≈19 pC N^–1^ which increased to ≈205 pC N^–1^ for the expanded cellular PP.

## 5 Piezoelectric Polymer Nanocomposites

In piezoelectric polymer composites, a filler with high piezoelectricity is impregnated in a polymer matrix. This helps to combine the advantages of both materials, that is, high piezoelectric constant and coupling factor of fillers and flexibility of polymers. The addition of fillers impacts the all‐inclusive properties of the resulting piezoelectric nanocomposites. The effect of polymer/filler interfaces in polymer nanocomposites has been discussed by Prateek et al.^[^ ^250^ ^]^ The type and concentration of fillers used, their shapes and morphology play a considerable part in enhancing the piezoelectric properties of nanocomposites. We have classified the fillers into three categories—conducting, non‐conducting, and hybrid fillers. Non conducting fillers include materials such as, BaTiO_3,_^[^ ^251^, ^252^, ^253^ ^]^ barium dititanate (BaTi_2_O_5_),^[^ ^254^ ^]^ ZnO,^[^ ^43^, ^255^, ^256^, ^257^ ^]^ PZT,^[^ ^258^ ^]^ potassium sodium niobate (KNN),^[^ ^259^ ^]^ etc., and are known to have the highest piezoelectric coefficients. Conducting fillers such as graphene,^[^ ^260^, ^261^ ^]^ MWCNTs,^[^ ^262^, ^263^ ^]^ carbon black,^[^ ^264^, ^265^ ^]^ etc., have been used substantially as well. Hybrid fillers comprise two or more components at the nanometer/micron scale, out of which at least one is conducting and the other is non‐conducting in nature. Examples of hybrid fillers include NaNbO_3_‐reduced graphene oxide (rGO),^[^ ^266^ ^]^ titanium oxide (TiO_2_)‐rGO nanotubes,^[^ ^267^ ^]^ manganese oxide (MnO_2_)/graphene/MWCNT hybrid,^[^ ^268^ ^]^ etc. In this section, we will perform an in‐depth review of piezoelectric properties of nanocomposites synthesized using the fillers mentioned above in PVDF and PDMS‐based polymer matrix.

### 5.1 Polyvinylidene Fluoride Based Nanocomposites

Among all‐polymer composites, PVDF based composites have been researched thoroughly in the past two decades and are considered to be the best option because of the greater piezoelectric coefficients of PVDF when compared to other polymers.^[^ ^269^ ^]^ This section summarizes the synthesis, piezoelectric behavior, and energy harnessing abilities of PVDF and PVDF copolymer‐based nanocomposites. There are three Sub‐sections—Sections 5.1.1. and 5.1.2. focus on nanocomposites prepared using non‐conducting and conducting fillers respectively. Section 5.1.3. focuses on nanocomposites prepared using hybrid fillers, as well as, the mixture of non‐conducting and conducting fillers.

#### 5.1.1 Nanocomposites Based on Non‐Conducting Fillers

This section gives an overview of nanocomposites synthesized using non conducting fillers such as BaTiO_3_,^[^ ^251^, ^252^, ^253^ ^]^ barium dititanate (BaTi_2_O_5_),^[^ ^254^ ^]^ ZnO,^[^ ^43^, ^255^, ^256^, ^257^ ^]^ PZT,^[^ ^258^ ^]^ and potassium sodium niobate (KNN).^[^ ^259^ ^]^ It focuses on the synthesis, piezoelectric properties, PENG fabrication methods, and energy harvesting performance of non‐conducting fillers/PVDF and PVDF copolymers‐based nanocomposites.

**Figure 15:**
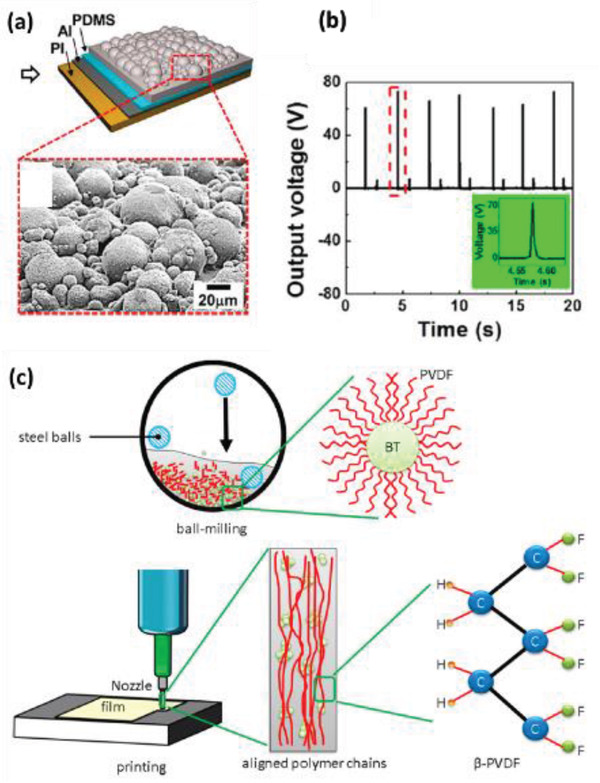
a) Schematic diagram of the PENG, showing the nanocomposite film consisting of BaTiO_3_ clusters. b) Rectified *V*_oc_ of the PENG with a full‐wave rectifier under periodic finger impartation. The inset is an enlarged view of a voltage pulse in the dashed line. a,b) Reproduced with permission.^[^ ^270^ ^]^ Copyright 2014, American Chemical Society. c) Schematic of the proposed process leading to increased *β*‐phase content in PVDF due to BaTiO_3_ filler addition. Reproduced with permission.^[^ ^271^ ^]^ Copyright 2017, American Chemical Society.

Among different non‐conducting fillers, BaTiO_3_ has been commonly used. Shin et al. prepared a nanocomposite film of hemispherically aggregated BaTiO_3_ NPs and P(VDF‐HFP) to construct a PENG.^[^ ^270^ ^]^ A solution was first prepared by infusing BaTiO_3_ NPs, P(VDF‐HFP), dimethylformamide (DMF) and acetone, followed by spin‐coating onto a Si substrate and curing at 80 °C. In the course of the curing process, hemispherical BaTiO_3_ clusters originated as a result of the evaporation of solvents, that is, acetone and DMF (**Figure** **15**a). To fabricate the PENG, the composite films were attached to PDMS‐coated Al electrodes on a polyimide substrate (Figure 15a). The formation of aggregated BaTiO_3_‐P(VDF‐HFP) clusters improved the piezoelectric output of the PENG by amplifying the entire dipole moment within the cluster and fostering an efficient structure for harvesting external stress. When the applied pressure was ≈0.23 MPa, *V*_OC_ of ≈75 V, and *I*_SC_ of ≈15 µA was generated by the PENG (Figure 15b), which provided sufficient power to operate a commercial LED. Later, they adjusted the ratio of acetone to DMF in the nanocomposite solution to obtain optimal BaTiO_3_ cluster formation with increased density.^[^ ^52^ ^]^ These optimizations contributed to substantially higher output of the NG, displaying the highest *V*_OC_ of ≈110 V and *I*_SC_ of ≈22 µA corresponding to a power density of ≈0.48 W cm^–3^ under identical pressure application.

Bodkhe et al. leveraged a unique 3D printing process to prepare PVDF/BaTiO_3_ NPs‐10 wt% nanocomposites in the absence of poling.^[^ ^271^, ^272^ ^]^ The obtained d_31_ value (18 pC N^–1^) was comparable to poled and stretched PVDF films. Figure 15c summarizes the formation process of aligned *β*‐phase chains in the course of 3D printing. Ball‐milling increased the activation sites on BaTiO_3_ NPs, that acted as nucleating sites for *β*‐phase due to strong O—H···F—C hydrogen interactions at BaTiO_3_/PVDF interfaces. The large pressure of ≈1 MPa exerted on the solution during extrusion resulted in crystallization of *β*‐phase in PVDF. The NP agglomerates prevented the reversion to *α*‐phase, leading to enhanced piezoelectric properties. A fully functional sensor was fabricated that produced the highest output voltage of 4 V upon finger tapping.

Siddiqui et al. embedded BaTiO_3_ NPs with high loading up to 40 wt% into P(VDF‐TrFE) using solution processing.^[^ ^273^ ^]^ The PENG produced a voltage of 9.8 V and *j* = 1.4 µA cm^–2^ when bent periodically due to the large effective piezoelectricity of P(VDF‐TrFE)/BaTiO_3_ nanocomposite. It also displayed high stability at different frequencies, without any notable drop in output after multiple bending cycles. The PENG was shown to scavenge energy from body movements and the energy was stored in a rechargeable micro battery. Later, he fabricated a PENG using electrospun P(VDF‐TrFE)/BaTiO_3_ nanofibers and demonstrated energy harvesting while walking when the PENG was situated inside a shoe.^[^ ^274^ ^]^ The PENG with 15 wt% BaTiO_3_ NPs generated 25 V voltage at 0.6 Hz walking frequency and 600 N load. Zhao et al. obtained a *V*_oc_ of 150 V and *I*_sc_ of 1500 nA by applying 10 MPa stress to the PENG.^[^ ^251^ ^]^ The high output was attributed to the well dispersed BaTiO_3_ NPs in the PVDF matrix, which increased piezoelectric potential and reinforced local stresses compared to pure PVDF. Li et al. showed that calcining BaTiO_3_ powders at 950°C increase the d_33_ value of the composites to a maximum of 25 pC N^–1^, compared to 5 pC N^–1^ with un‐calcined BaTiO_3_.^[^ ^253^ ^]^ The calcination process accelerated the transformation of BaTiO_3_ from rhombohedral phase (no piezoelectric properties) to tetragonal (piezoelectric properties) which helped in polarization.

Pereira et al. investigated the energy harnessing efficiency of P(VDF‐TrFE)/BaTiO_3_ nanocomposites synthesized by solution casting^[^ ^275^ ^]^ and electrospinning.^[^ ^276^ ^]^ BaTiO_3_ nanoparticles with a mean size of 10, 100, and 500 nm were used at a loading of 0–20 wt% in the P(VDF‐TrFE) matrix. For the solution cast samples, the highest output of ≈0.28 µW was obtained with 10 nm BaTiO_3_ particles at 20 wt% loading and with 100 and 500 nm particles at 5 wt% loadings.^[^ ^275^ ^]^ The output power decreased for larger BaTiO_3_ particle sizes at contents above 5 wt% as a result of increased mechanical stiffness possessed by the composites. For the nanofibers, interestingly, the inclusion of BaTiO_3_ NPs did not influence their diameter, distribution or power output. Sahu et al. reported an output voltage of 7.2 V, current of 38 nA, and power density of 0.8 µW cm^–2^ from a PENG containing 10 wt% BaTiO_3_/PVDF composite film prepared by solvent casting.^[^ ^277^ ^]^ The PENG was used to harvest energy from activities like finger tapping, dropping of coin and air blowing.

Lee et al. synthesized highly‐aligned PVDF/BaTiO_3_ nanofibers with diameters of ≈200 nm by electrospinning.^[^ ^278^ ^]^ The composite nanofibers with 16 wt% BaTiO_3_ NPs exhibited the highest output of 0.48 V; 1.7 times greater than pristine PVDF fibers. Kakimoto et al. prepared fibrous BaTiO_3_/PVDF (30/70 v%) composites by drawing, annealing and uniaxially stretching, followed by poling to form the *β* phase.^[^ ^279^ ^]^ The induced strain in the 31 direction was enhanced up to 0.35% in the composites having an 83% orientation ratio of BaTiO_3_ fibers, compared to the reference specimens using spherical BaTiO_3_ particles. Due to a higher aspect ratio of fibers than powders, they were more likely to contact one another to form connected passages which resulted in enhanced power output.

**Figure 16:**
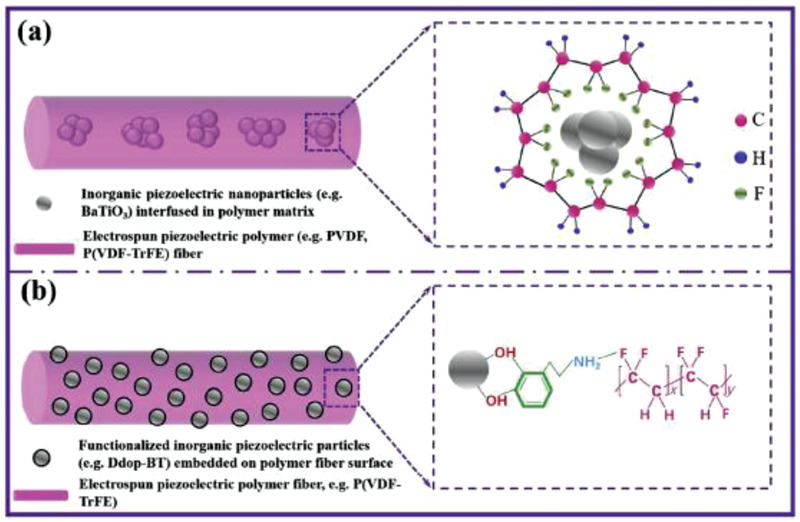
Schematic diagram showing a) BaTiO_3_ NPs showing aggregation in PVDF matrix; inset showing the formation of *β* phase on BaTiO_3_ NPs. b) Pdop‐BaTiO_3_ showing good dispersion on the surface of electrospun P(VDF‐TrFE) fiber; inset displaying the interfacial interactions between BaTiO_3_, Pdop, and P(VDF‐TrFE). Reproduced with permission.^[^ ^280^ ^]^ Copyright 2020, Elsevier Ltd.

Guan et al. developed a high‐performance PENG using an electrospun nanocomposite of polydopamine (Pdop) modified BaTiO_3_ NPs and P(VDF‐TrFE).^[^ ^280^ ^]^ To increase interaction and compatibility between BaTiO_3_ NPs and P(VDF‐TrFE), the BaTiO_3_ NPs were functionalized with Pdop via in situ polymerization.^[^ ^281^ ^]^ In contrast with previous approaches (**Figure** **16**a), the Pdop modified BaTiO_3_ NPs were anchored onto the outer surface of P(VDF‐TrFE) fibers (Figure 16b) to prevent agglomeration and to augment the interfacial density in the nanocomposites. A significantly higher output of 6 V and 1.5 µA was exhibited by the PENG in comparison to the PENG with only P(VDF‐TrFE) membrane (1.25 V and 0.6 µA) under 700 N applied force and 3 Hz impact frequency. This was attributed to increased polarization caused by high density of interfaces in the microstructure.

Chen et al. built a high‐performance PENG from a micropillar array of P(VDF‐TrFE)/BaTiO_3_ nanocomposite, synthesized using BaTiO_3_ NPs of ≈200 nm diameter at an optimized content of 20 wt%.^[^ ^282^ ^]^ The d_33_ values of P(VDF‐TrFE) film was 14.6 pC N^–1^ and P(VDF‐TrFE)/BaTiO_3_ composites was 35.3 pC N^–1^. The PENG exhibited an enhanced voltage of 13.2 V and *j* = 0.33 µA cm^−2^ in comparison to bulk P(VDF‐TrFE) film due to enhanced piezoelectricity and mechanical flexibility of the compressed micropillar arrays.

Yaqoob et al. assembled a PENG using PVDF/BaTiO_3_‐55 wt% nanocomposite and surface functionalized n‐type graphene (n‐Gr).^[^ ^283^ ^]^ Graphene was modified using an amino compound, that oriented the negatively charged carboxylate groups over the graphene surface. The tri‐layered PENG structure was achieved by placing PVDF/BaTiO_3_ nanocomposite films, one on either side of the n‐Gr layer. Under the application of 2 N force, the PENG provided a maximum Vp‐p of 10 V and current of 2.5 µA due to the critical role of n‐Gr in aligning the dipoles unidirectionally.

**Figure 17:**
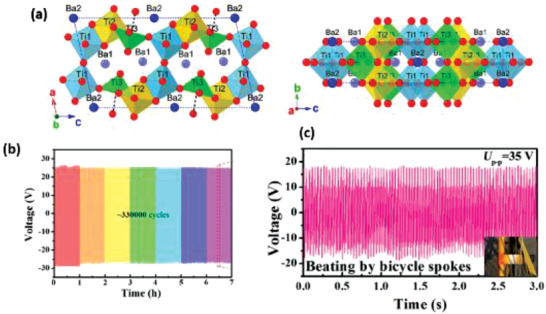
a) Crystalline structure diagram of BaTi_2_O_5_ with the A2/m spacing group. The 5 vol% BT2/PVDF PENG showing b) stability and durability under vibration condition and c) voltage generation by the beating of bicycle spokes. Reproduced with permission.^[^ ^254^ ^]^ Copyright 2018, Elsevier Ltd.

Fu et al., constructed an energy harvester using a nanocomposite of PVDF and oriented BaTi_2_O_5_ (BT2) nanorods.^[^ ^254^ ^]^ Unlike BaTiO_3_ which has a perovskite structure, the three Ti sites in BT2 forms two [TiO_6_] octahedrons: [Ti1O_6_] (blue), [Ti_2_O_6_] (yellow) and one [Ti_3_O_5_] pentahedron (green); these units are attached to form a monoclinic lattice (**Figure** **17**a). Hot pressing aligned the BT2 nanorods horizontally in the PVDF matrix and rendered a textured structure to them that ensured an increase in power generation. The energy harvester with 5 vol% BT2/PVDF generated the highest output corresponding to a power density of 27.4 µW cm^–3^ and high stability even after 330 000 vibration cycles (Figure 17b). To harvest energy from wheel motion, the PENG was affixed to a bicycle (Figure 17c) and could potentially power sensors for detecting travel speed, atmospheric temperature, and humidity.

Indolia and Gaur showed that ZnO NPs of 1–9 wt% loading were uniformly dispersed in the PVDF matrix.^[^ ^284^ ^]^ The highest crystallinity of 54.4% was obtained for ZnO‐9 wt% composites due to rapid crystallization caused by the nucleating effect of ZnO fillers. Jana et al. used ZnO NPs to induce an entirely *γ*‐crystalline phase in PVDF without poling.^[^ ^285^ ^]^ The ZnO/PVDF composite films with 1 wt% ZnO NPs produced the highest amount of *γ*‐phase of ≈92% and d_33_ value of −6.4 pC N^–1^. The PENG produced a *V*_oc_ of 28 V and *I*_sc_ of 450 nA by repeated human finger imparting, which was enough to turn on 48 blue LEDs instantly.

Bhunia et al. made flexible ZnO/PVDF composite films by the sol–gel technique, followed by poling them in a vacuum.^[^ ^286^ ^]^ A ten‐times increase in output voltage was observed upon poling the ZnO/PVDF nanocomposites. The composites with 0.2 mol% ZnO concentration exhibited a voltage of ≈4 V and much higher value d_33_ value of 900 pC N^–1^, compared to 20–28 pC N^–1^ for PVDF^[^ ^31^, ^136^ ^]^ and 12.3 pC N^–1^ for ZnO.^[^ ^287^ ^]^ Bafqi et al. prepared 15 wt% ZnO/PVDF fibers with optimized electrospinning conditions and obtained a higher output of 1.1 V in comparison to 0.351 V for neat PVDF due to higher *β* phase formation.^[^ ^288^ ^]^

Han et al. prepared an extremely sensitive impact sensor using P(VDF‐TrFE)/ZnO nanocomposite films with different ZnO contents (1.5–12.5 wt%).^[^ ^289^ ^]^ When hit by a ball of 65 g, the sensor containing 7.5 wt% ZnO NPs generated the highest voltage, that is, 5.5 times of pristine P(VDF‐TrFE). The d_33_ value was also highest (32.2 pC N^–1^) at 7.5 wt% ZnO loading, beyond which it decreased due to agglomeration of ZnO NPs. Nguyen et al. showed that nanocomposites with 10 wt% ZnO NPs displayed a high d_33_ value of 19–22 pC N^–1^ irrespective of the type of ZnO used, indicating that there was no impact of surface functionalization.^[^ ^290^ ^]^ P(VDF‐TrFE) nanocomposites containing Al_2_O_3_^[^ ^291^ ^]^ and LiNbO_3_^[^ ^292^ ^]^ NPs also showed similar behavior.

**Figure 18:**
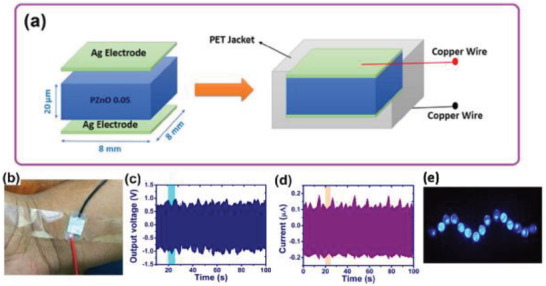
a) Schematic diagram of the fabricated PENG. b) Photograph of attachment of the PENG on the hand. c,d) Output voltage and current under the touching condition of the device on hand. e) Snapshot of the lighting of blue LEDs driven using PENG under periodic finger impartation. Reproduced with permission.^[^ ^293^ ^]^ Copyright 2018, Elsevier Ltd.

Thakur et al. assembled a self‐poled energy harvester (**Figure** **18**a) by incorporating ZnO NPs in the PVDF matrix via the in situ process.^[^ ^293^ ^]^ ZnO NPs had a negative surface charge, therefore it interacted with the positive ‐CH_2_ dipoles in PVDF via ion‐dipole or electrostatic attraction. These interactions and charge carrier aggregation at ZnO/PVDF interfaces led to ≈84% *β* phase amount and ≈50 pC N^–1^ d_33_ value in the nanocomposites. The PENG revealed a high‐power density of ≈32.5 mW cm^–3^ with long durability when touched periodically by a human finger. It also generated uninterrupted power due to vibration from blood circulation in the human body (Figure 18b–d). The PENG was shown to operate 15 blue LEDs connected serially (Figure 18e) and 1 µF charging capacitor within a short period as a result of repeated finger impartation.

Singh et al. showed that the polar *β* phase of PVDF can be improved from 48.2% to 76.1% by embedding ZnO NRs into the PVDF matrix in the absence of any mechanical or electrical treatment.^[^ ^294^ ^]^ The d_33_ value of the 15 wt% ZnO/PVDF film was ≈−1.17 pC N^–1^ and a maximum *V*_oc_ of 1.81 V, *I*_sc_ of 0.57 µA were obtained when hit by a cylinder with a force of ≈15 kPa. The power density was calculated as 0.21 µW cm^–2^ across a load resistance of 7 MΩ, which demonstrates the feasibility of using it for small‐scale electronic devices. The piezo potential was generated due to *β* phase nucleation in PVDF upon application of external force. Choi et al. reported power enhancement of the PENG by ≈17 times using a nanocomposite of ZnO NWs/PVDF compared to pristine PVDF.^[^ ^295^ ^]^ ZnO NWs were synthesized by the HMTA method with a growth density of ≈10 µm^−2^. The maximum voltage and current were 0.4 V and 30 nA, respectively at a strain rate of 3.2%.

**Figure 19:**
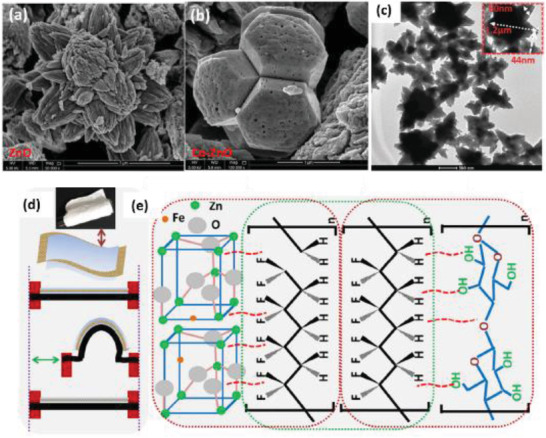
a) SEM images of undoped ZnO and b) Co‐doped ZnO nanorods. a,b) Reproduced with permission.^[^ ^296^ ^]^ Copyright 2018, Springer Nature. c) TEM image of Fe‐doped ZnO particles. Reproduced with permission.^[^ ^298^ ^]^ Copyright 2018, The Royal Society of Chemistry. d) Sample flexibility and schematic of bending experiment. e) Interaction mechanism in the nanocomposites consisting of dual layers of P(VDF‐HFP)/Fe‐ZnO and P(VDF‐HFP)/CNC. Reproduced with permission.^[^ ^299^ ^]^ Copyright 2019, Elsevier Ltd.

Parangusan et al. studied P(VDF‐HFP)/Co doped‐ZnO nanofiber composites, synthesized via electrospinning.^[^ ^296^ ^]^ The Co‐doped ZnO NRs were prepared by hydrothermal method and had hexagonal cross‐sections, as opposed to flower‐like morphology of undoped ZnO NRs (**Figure** **19**a,b). The highest output voltage obtained was 2.8 V for 2 wt% Co‐ZnO/PVDF‐HFP nanofibers. The incorporation of these fillers in P(VDF‐HFP) improved *β*‐phase nucleation and stabilization due to interactions of oppositely charged fillers with —CF_2_—/—CH_2_— dipoles present in P(VDF‐HFP). The same group achieved exceptional piezoelectric performance in 0.5 wt% Ni‐doped ZnO/P(VDF‐HFP) nanocomposites with a d_33_ value of 20 pC N^–1^.^[^ ^297^ ^]^

Parangusan et al. illustrated the usage of *γ*‐irradiation in imparting desired piezoelectric properties to Fe‐doped ZnO/PVDF nanocomposites.^[^ ^298^ ^]^ The *γ*‐irradiated nanocomposites containing 2 wt% Fe‐ZnO NPs exhibited 55% *β*‐phase crystallinity and d_33_ = 9.44 pC N^–1^, which was much higher than neat PVDF (F*β*) = 32%, d_33_ = 6.15 pC N^–1^) and non‐*γ* irradiated composites. This was due to the influence of star‐like morphology (Figure 19c) and dispersion of Fe‐ZnO, improved interactions with PVDF and crosslink creation induced by the *γ*‐irradiation process. The PENG generated a maximum voltage of 2.4 V and power density of 1.17 µW cm^–2^ during stretching, bending and rolling motions of the nanocomposites, indicating their potential use in flexible electronics.

Ponnamma et al. designed a PENG from electrospun nanofibers of P(VDF‐HFP), cellulose nanocrystals (CNC, 2 wt%) and Fe‐doped ZnO (2 wt%).^[^ ^299^ ^]^ The nanocomposite had two layers; the first layer consisted of CNC/P(VDF‐HFP) and the second layer consisted of Fe‐doped ZnO/P(VDF‐HFP) (Figure 19d). The hybrid nanocomposite produced a maximum of 12 V from mechanical vibrations, 5.5 V from the movement of the human elbow, and 1.1 V from cloth folding motions. The numerous —OH groups present on a CNC chain forms H bonds with O_2_ atoms of adjacent chains; this bonding generates spontaneous electrical dipoles in CNC and results in polarization upon stress application (Figure 19e). Similar bonding takes place in Fe‐doped ZnO, creating dipole moment as shown in Figure 19e. Fashandi et al. developed a PENG from electrospun PVDF/CNCs with various CNC loadings (1–5 wt%).^[^ ^300^ ^]^ The 3 and 5 wt% nanocomposites charged a 33‐µF capacitor and lit up a commercial LED for 30 s due to *β* phase nucleation and suppression of the *α* phase.

Si et al. added zinc titanate (ZTO) NPs in PVDF, which acted as an agent for heterogeneous nucleation, provided an enormous surface area for PVDF adsorption and enabled accelerated *γ*‐phase nucleation.^[^ ^301^ ^]^ The PENG containing 2 wt% ZTO produced a stable voltage of ≈25.7 V, current of ≈1.2 µA, corresponding to a power density of ≈8.22 µW cm^–2^, when a pressure of ≈16.5 kPa was applied by finger. It could harvest energy from several energy sources like human body movement and mechanical vibrations.

**Figure 20:**
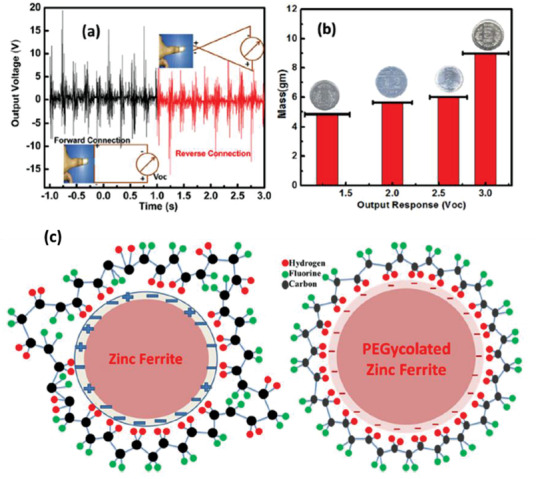
a) Output voltage performance of PVDF/ZF(PEG)‐12 wt% nanocomposites. b) Impact sensing capability of the nanocomposites. c) Proposed interactions of untreated and surface‐treated zinc ferrite with PVDF. Reproduced with permission.^[^ ^303^ ^]^ Copyright 2017, Elsevier B.V.

Chinya and Sen reported enhanced *β* phase content of ≈88% in nanocomposites of PVDF and ascorbic acid assisted zinc ferrite (ZF(ASC)).^[^ ^302^ ^]^ Zinc Ferrite NPs were synthesized by utilizing ascorbic acid as a chelating agent followed by its surface modification with tetraethyl orthosilicate (TEOS). After poling, the PVDF/1.5 wt% ZF(ASC) nanocomposites displayed ≈2.20 V output voltage on single finger touch due to improved interaction between TEOS modified ZF particles and PVDF matrix. In a later work, they modified ZF NPs with polyethylene glycol (PEG) and showed a maximum *β* phase of ≈92% and Voc of 18 V for PVDF/ZF(PEG)‐12 wt% nanocomposites^[^ ^303^ ^]^ (**Figure** **20**a). Coins of different weights were dropped on PVDF/ZF‐PEG film and the sensitivity response of each was plotted (Figure 20b). The PEG layer helped to prevent agglomeration of NPs and entrapped more surface negative charge; higher negative charge helped in the orientation of more —CH_2_ dipoles in PVDF, resulting in a higher *β* phase fraction (Figure 20c). Recently, Chinya et al. studied the influence of different ZF architectures, that is, nearly spherical, nearly cubic and rod‐like, added to PVDF with different contents.^[^ ^304^ ^]^ It was observed that nano‐rods with 3 wt% loadings were more favorable for *β* phase formation and stabilization. Thus, they exhibited a maximum piezoresponse of ≈39.10 V and a power density of 2.96 µW mm^–3^. This was attributed to the desirable ‘in‐plane orientation of the nanorod structure in PVDF that helped to gather the all‐trans (TTTT) conformation and create an extended TTTT conformation corresponding to *β*‐phase. Similar work was done by Mishra et al. using gallium ferrite (GaFeO_3_) NPs of 10–30 wt% loading.^[^ ^305^ ^]^ Characterization of the 30 wt% GaFeO_3_/PVDF nanocomposites showed that *α*, *β*, and *γ* phases were present. They exhibited a maximum voltage and current of 4 V and 4 nA.

Tiwari and Srivastava synthesized highly flexible PZT/PVDF nanocomposite films and showed enhanced piezoelectric properties due to an increase in *β* phase with PZT addition.^[^ ^306^ ^]^ The composite films with 10–30 v% PZT showed an increase in d_33_ from 60 to 84 pC N^–1^, compared to ‐32 pC N^–1^ for neat PVDF films. Similar studies were done by several researchers on 0–3 PZT/PVDF composites, showing an increase in d_33_ compared to neat PVDF films.^[^ ^24^, ^117^, ^258^, ^307^, ^308^, ^309^, ^310^, ^311^ ^]^

Bairagi et al. demonstrated a flexible PENG composed of KNN nanorods (0–6 wt%) in the PVDF matrix followed by corona poling.^[^ ^312^ ^]^ The PENG made with PVDF/KNN NRs‐ 4 wt% exhibited the highest F(*β*) of 26% and generated ≈3.7 V voltage, 0.326 µA current when tapped by finger. The —OH groups present on the surface of KNN NRs helped to nucleate and stabilize the polar *β* phase through interactions between —OH and —CH_2_/CF_2_— dipoles in PVDF. Beyond 4 wt% KNN, nucleation of *β*‐phase was restricted, and more defects were created in the PVDF matrix, which resulted in declined output.

Haddadi et al. added SiO_2_ NPs to PVDF^[^ ^313^ ^]^ for improving specific properties such as piezoelectric, mechanical characteristics, hydrophilicity, etc.^[^ ^314^, ^315^, ^316^ ^]^ The surface charge of SiO_2_ NPs is naturally negative because of the presence of OH— groups. In the electrospun nanocomposites, *β*‐phase nucleation was the result of effective interactions between CH_2_ groups in PVDF and SiO_2_ NPs. Maximum F(*β*) obtained was 84% and the output voltage was ≈25 V at 0.5 wt% SiO_2_ loading under the application of 13.9 N force.

Dutta et al. observed an F(*β*) of 85% at a critical nickel oxide (NiO) loading of 0.75 wt% in PVDF.^[^ ^317^, ^318^ ^]^ The NiO NPs were hexagonal shaped with a mean size of 44 nm and were synthesized via the hydrothermal method. The interactions between negatively charged NiO NPs and positively charged CH_2_ groups in PVDF led to the alignment of TTT conformation in PVDF, resulting in *β* phase nucleation.

**Figure 21:**
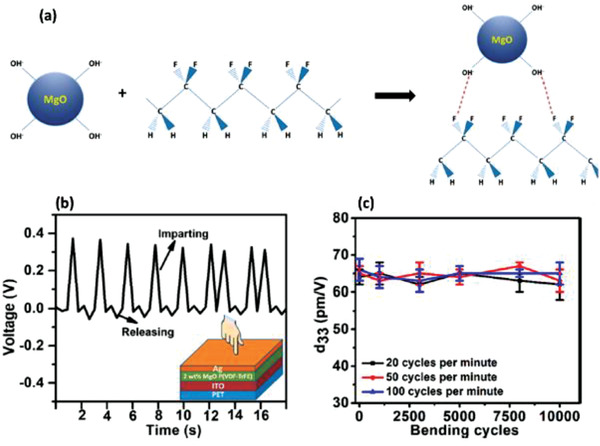
a) Schematic diagram showing hydrogen bond formation in P(VDF‐TrFE)/MgO nanocomposites. b) Voltage generated by the 2 wt% MgO/P(VDF‐TrFE) nanocomposite‐based PENG on continuous finger tapping. c) Variation of d_33_ coefficient as a function of bending cycles at different strain rates. Reproduced with permission.^[^ ^319^ ^]^ Copyright 2017, American Chemical Society.

Singh et al. fabricated a flexible PENG employing a nanocomposite of 50 nm‐sized MgO NPs uniformly distributed in a P(VDF‐TrFE) matrix.^[^ ^319^ ^]^ The d_33_ value for P(VDF‐TrFE)/MgO‐2 wt% nanocomposite was ‐65 pC N^–1^ compared to −40 pC N^–1^ for P(VDF‐TrFE). **Figure** **21** schematically shows the interactions between —OH groups present on the surface of MgO with F atoms of P(VDF‐TrFE) chains, resulting in hydrogen bond formation. This was the primary reason behind enhanced crystallinity and stabilization of the *β* phase in the nanocomposites. The PENG generated an output voltage of about 0.4 V on repeated touching (Figure 21b). It also demonstrated exceptional durability when put through 10 000 bending cycles as the d_33_ values remained unchanged irrespective of the bending speed (Figure 21c).

Martin et al. investigated the influence of the surface charge of cobalt ferrite (CoFe_2_O_4_) NPs on PVDF *β*‐phase crystallization.^[^ ^320^ ^]^ CoFe_2_O_4_ NPs‐5 wt% was coated with sodium dodecyl sulfate (SDS), cetrimonium bromide (CTAB) and triton X‐100 surfactants, which induced negative, positive and nearly zero surface charge respectively. Higher F(*β*) of 90% and d_33_ of 33 pC N^–1^ were obtained in CoFe_2_O_4_/PVDF than CoFe_2_O_4_‐SDS/PVDF nanocomposites (F(*β*) = 30%, d_33_ = 23 pC N^–1^). The reason behind this was that the surface of CoFe_2_O_4_ NPs had a higher negative charge than CoFe_2_O_4_‐SDS, therefore electrostatic interactions were stronger. The CoFe_2_O_4_‐Triton and CoFe_2_O_4_‐CTAB NPs with nearly zero and the positive surface charge did not allow *β*‐phase formation.

Ouyang et al. prepared nanocomposites by embedding 6 nm‐sized magnetites (Fe_3_O_4_) NPs into PVDF and showed via TEM analysis that they were uniformly distributed in PVDF.^[^ ^321^ ^]^ The highest d_33_ value was 37 pC N^–1^ and F(*β*) was 72.5% for the poled PVDF/Fe_3_O_4_‐2 wt% nanocomposites; about five times higher than non‐poled ones. The addition of Fe_3_O_4_ NPs enhanced *β*‐phase content as a result of tiny particle size and negative surface charge. The radius of gyration, *R*_g_, of PVDF was calculated to be 24 nm, which was greater than the average diameter of Fe_3_O_4_ NPs (6 nm). Therefore, when these NPs were added to PVDF, the polymer swelled to increase its *R*_g_;^[^ ^322^ ^]^ this increase in Rg was manifested by PVDF transforming to its more extended *β* form.

Parangusan et al. reported enhanced piezoelectric properties by incorporating cerium (Ce) doped iron oxide (Fe_2_O_3_) and Ce doped cobalt oxide (Co_3_O_4_) NPs into PVDF.^[^ ^323^ ^]^ This was ascribed to the reduced size of crystals and greater electrostatic interactions at the NP/PVDF interface. The flexible PENGs built from PVDF/(Ce‐Fe_2_O_3_)‐ 2 wt% and PVDF/(Ce‐Co_3_O_4_)‐ 2 wt% fibers produced Vp‐p of 20 V and 15 V and current densities of 0.010 and 0.005 µA cm^–2^ when 2.5 N force was applied.

The effect of clay as a filler on the structure, morphology and *β*‐phase formation in PVDF has been studied extensively.^[^ ^179^, ^324^, ^325^, ^326^, ^327^ ^]^ Ma et al. investigated the impact of lithium‐ion (Li^+^) doping and adsorbed water on the piezoelectric performance of Montmorillonite (Mt)/P(VDF‐HFP) nanocomposites.^[^ ^328^ ^]^ Mt is a clay consisting of alumina octahedral sheets inserted between two tetrahedrally silica sheets, containing exchangeable cations such as Na^+^, Ca^2+^, Mg^2+^, and Fe^2+^. Lithium‐ion was used to replace Na^+^ and Ca^2+^ ions in Mt interlayers to improve the polarizability of the nanocomposites. The adsorbed H_2_O in Mt induced strong polarization in P(VDF‐HFP) via hydrogen bond formation between F atoms of P(VDF‐HFP) and H_2_O, which led to the formation of *β*‐phase. Mt‐Li/P(VDF‐HFP) nanocomposites with 15 wt% Mt‐Li and 1.72 wt% H_2_O exhibited the highest d_33_ value of ≈45 pC N^–1^ and the highest voltage and current. Jahan et al. prepared PVDF nanocomposites consisting of micro calcium carbonate (CaCO_3_) particles in varying amounts of 30–40 wt% and Mt via twin‐screw extrusion and uniaxial stretching at *R* = 5.^[^ ^329^ ^]^ Pure PVDF and nanocomposites with just 40 wt% CaCO_3_ did not exhibit any piezoelectricity. Due to enhanced *β* phase content of almost 100% and dipolar orientation induced by stretching, the nanocomposite containing 40 wt% CaCO_3_ and 3 wt% Mt. displayed the maximum d_33_ value of 30.6 pC N^–1^.

Rahman et al. introduced laponite nano‐clay mineral into the PVDF matrix by solvent evaporation and induced up to 98% *γ* phase.^[^ ^330^ ^]^ The chemical formula of laponite is Na_0.7_Si_8_Mg_5.5_Li _0.3_O_20_(OH)_4_; it is composed of layered silicates, stacked to form interlayers as described above for Mt. Laponite enhances toughness as well as electromechanical coupling effect of the nanocomposites, making it effective for building a durable energy harvester. PVDF/laponite‐0.5 wt% nanocomposites generated excellent output with the highest values of 6 V and 70 nA when a compressive force of 300 N was applied by hand punch. Tiwari et al. prepared electrospun nano‐clay/PVDF nanofibers with nano‐clay concentrations of 0, 5,10,15, 20 wt%.^[^ ^331^ ^]^ The nanofibers with 15 wt% nano clay had nearly 90% *β* phase and were determined to be tougher and stiffer, making them relevant for device applications. A maximum Vp‐p of 70 V and power density of 68 mW cm^–2^ was generated by the PENG due to greater charge separation in the nanofibers under an applied load. It was able to harvest energy from several forms of human movements like walking, twisting, bending, foot and finger tapping. Khalifa et al. designed a force sensor using halloysite nanotubes (HNT)/PVDF nanofibers by electrospinning.^[^ ^332^ ^]^ It produced the highest output voltage at 10 wt% HNT loading due to the highest *β* phase amount.

**Figure 22:**
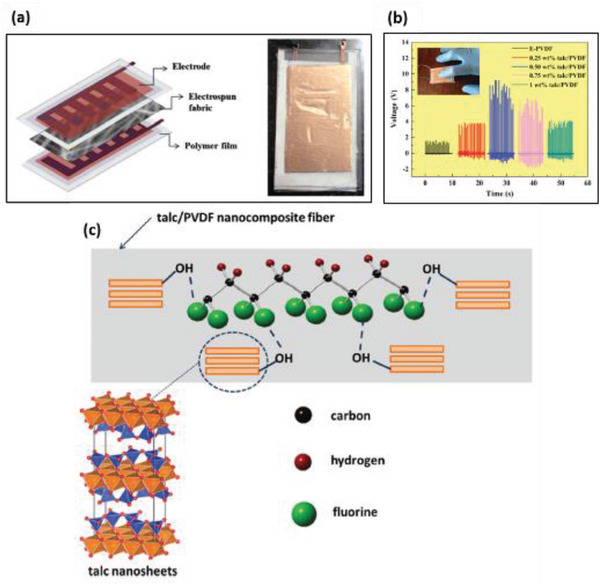
a) SEM micrograph of the electrospun 0.50 wt% talc/PVDF composite nanofibers. b) The output voltage of the PENG obtained by continuous finger tapping using PVDF and talc/PVDF nanocomposite fibers. c) Proposed mechanism of interactions between talc nanosheets and PVDF chains in the electrospun nanofibers. Reproduced with permission.^[^ ^333^ ^]^ Copyright 2020, The Royal Society of Chemistry.

Shetty et al. fabricated a PENG using electrospun talc/PVDF nanofibers (**Figure** **22**a) and showed that 0.5 wt% talcs caused nucleation of 89.6% *β* phase.^[^ ^333^ ^]^ The PENG with PVDF/talc‐0.50 wt% fibers generated a *V*_OC_ of 9.1 V with an adjacent power density of 1.12 µW cm^–2^ when a tapping force of 3.8 N was repeatedly applied by human finger (Figure 22b). Talc has a chemical formula of Mg_3_Si_4_O_10_(OH)_2_ and it is part of the family of clay minerals, that is, the phyllosilicate group. Talc structure consists of an octahedral sheet of MgO_4_(OH)_2_ as the intermediate layer between two layers of tetrahedral silica sheets (SiO_4_). The preferred alignment of ‐CH_2_/‐CF_2_ dipoles in the course of electrospinning and hydrogen bonding between PVDF —CF_2_ groups and —OH groups present in talc resulted in *β* phase nucleation (Figure 22c).

Harstad et al. synthesized gadolinium silicide (Gd_5_Si_4_)/PVDF nanocomposite films with high piezoelectric properties via the phase‐inversion method.^[^ ^334^ ^]^ The PVDF/Gd_5_Si_4_‐ 5 wt% nanocomposites displayed an enhanced F(*β*) of 81% and crystallinity of 58% compared to F(*β*) of 49% and crystallinity of 46% for pristine PVDF. The interfacial interactions of Gd_5_Si_4_ with PVDF interfaces resulted in preferential *β* phase crystallization as evidenced by FTIR. The PENG provided a much higher and stable voltage output when subjected to 2–3 N force at 5–10 Hz frequency.

Maity et al. reported an ultrasensitive PENG using electrospun 2D molybdenum sulphide (MoS_2_)/PVDF nanofibers.^[^ ^335^ ^]^ The PENG showed seventy times improvement in acoustic sensitivity compared to nano sensors built from neat PVDF nanofibers and was capable of charging a 9 V capacitor within 44 s. Due to the integrated semiconducting and piezoelectric properties of 2D‐MoS_2_, enhanced performance was obtained from the PENG. It could be used to power biomedical nano sensors for monitoring heartbeat, mapping pressure of footsteps and detecting speech signal abnormalities.

From the literature discussed in this section, it is evident that the addition of non‐conducting fillers to PVDF matrix enhances piezoelectric output from the PENG compared to PENGs using PVDF only. PVDF based copolymers have higher piezoelectric constants, therefore PVDF copolymer nanocomposites generated higher output voltages and currents compared to PVDF nanocomposites in general. It was found that poling was a mandatory step for nanocomposites where BaTiO_3_ was used as a filler. Whereas nanocomposites with fillers such as ZnO, Co/Ni/Fe doped ZnO, clay, MgO, Fe_3_O_4_, etc. did typically not use poling. The filler content varied between 0 and 55 wt% and nanocomposite synthesis methods included electrospinning, solvent casting, spin casting, drop‐casting, and hot pressing. Furthermore, the output performance was found to depend on the type of filler used, their concentration, the surface modifying agents of the fillers and synthesis conditions. Among the summarized literature, the highest output voltage of 70 V was obtained from electrospun PVDF/nano‐clay composites containing 15 wt% nano‐clay when tapped by a human finger.^[^ ^331^ ^]^

#### 5.1.2 Nanocomposites Based on Conducting Fillers

This section summarizes the literature on PVDF nanocomposites prepared using conducting fillers such as Ag,^[^ ^336^ ^]^ MWCNTs,^[^ ^337^ ^]^ reduced graphene oxide,^[^ ^338^ ^]^ and carbon black.^[^ ^264^ ^]^ A brief overview of their synthesis, piezoelectric properties, PENG fabrication methods and energy harvesting performance of conducting fillers/PVDF and PVDF copolymers‐based nanocomposites is presented in this section.

Paik et al. examined the effect of Ag‐NPs on piezoelectric characteristics of Ag/P(VDF‐TrFE) nanocomposites.^[^ ^336^ ^]^ At an Ag concentration of 0.005 v%, d_33_ had a maximum value of 20.23 pC N^–1^, beyond which it decreased to 13.45 pC N^–1^ due to an increase in Ag% up to 0.01 v%. The presence of Ag NPs up to an optimum amount enabled the dipoles to align well under an external electric field, which led to the increased polarization of the composites and enhanced piezoelectric properties.

**Figure 23:**
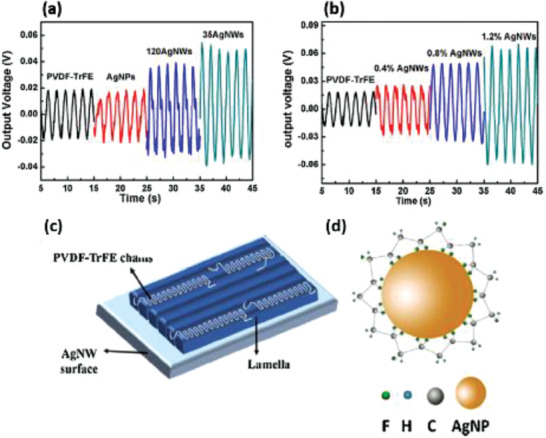
The piezoelectric response of a) P(VDF‐TrFE) nanocomposites with Ag NPs and Ag NWs. b) P(VDF‐TrFE)/Ag NWs nanocomposites with various loadings of Ag NWs (35 nm in diameter). c) Schematic of crystal structure on Ag NW surface. d) Arrangement of P(VDF‐TrFE) chains absorbed on Ag NP surface. Reproduced with permission.^[^ ^339^ ^]^ Copyright 2016, Elsevier B.V.

Chen et al. added Ag NPs with 20 nm diameter and two kinds of Ag NWs with diameters of 120 and 35 nm into P(VDF‐TrFE) at 0.8 wt% loadings.^[^ ^339^ ^]^ Formation of *β*‐phase in P(VDF‐TrFE) was promoted by Ag NWs, but not by Ag‐NPs. Pristine P(VDF‐TrFE) and nanocomposite of P(VDF‐TrFE)/Ag NPs exhibited low output voltage of 17–19 mV (**Figure** **23**a), while nanocomposite of P(VDF‐TrFE)/Ag NWs (120 nm diameter) showed the higher output of 36 mV, which increased further to 48 mV for 35 nm NW diameter (Figure 23b). In the case of Ag NWs, due to their planar surfaces, P(VDF‐TrFE) could be readily packed in a way that promotes *β*‐phase formation (Figure 23c). Whereas for Ag NPs, because of their spherical surface, it is hard for P(VDF‐TrFE) molecular chains to overcome the steric hindrance and therefore, they cannot easily form planar structure to facilitate crystallization (Figure 23d).

Issa et al. showed an increase in *β*‐phase by nearly 8% with the addition of Ag NPs between 0.4 and 0.6 wt% in electrospun PVDF nanofibers.^[^ ^340^ ^]^ A voltage of 2 V was obtained from the nanofibers with 0.4 wt% Ag due to aligned *β*‐phase present in the samples as a result of electrospinning. Li et al. constructed a pressure sensor from electrospun PVDF/Ag NWs‐1.5 wt% with a high sensitivity of 30 pC N^–1^, compared to 18 pC N^–1^ for pure PVDF.^[^ ^341^ ^]^ The good distribution of Ag NWs in PVDF facilitated interactions between them and led to increased *β*‐phase content.

**Figure 24:**
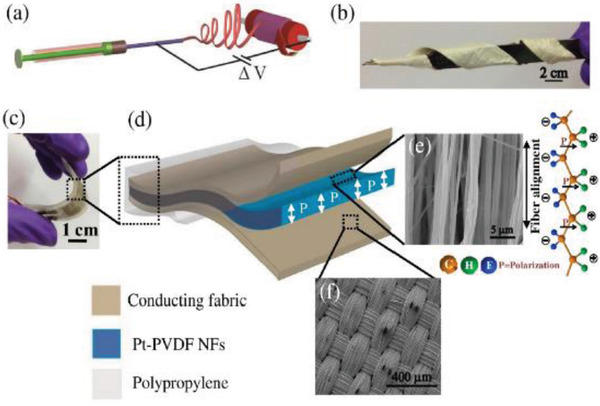
a) Schematic demonstration of the electrospinning setup. b) Outstanding flexibility illustration of Pt/PVDF nanofibers mat by rolling around an irregular object. c) Photograph of the flexible PENG with d) 3D design showing e) SEM image of aligned arrays of Pt/PVDF nanofibers along the surface direction with their polarization direction along the thickness direction. f) Interlocking micro‐fiber arrays of conducting fabric. Reproduced with permission.^[^ ^342^ ^]^ Copyright 2018, Elsevier Ltd.

Ghosh and Mandal fabricated an all‐fibre PENG using highly aligned, electrospun PVDF/Pt nanofiber arrays as shown in **Figure** **24**a.^[^ ^342^ ^]^ The nanofibers were extremely flexible as depicted in Figure 24b,c. The Pt/PVDF nanofibers were bonded in between conducting fabrics containing interlocked microfibers arrays (Figure 24d). Polypropylene was used to laminate the entire structure for environmental protection followed by connecting electrical leads to the top‐bottom electrodes. The composite nanofibers exhibited a 99.9% *β*‐phase and d_33_ value of 44 pC N^–1^. The PENG displayed high performance—*V*_oc_ ≈ 30 V, *I*_sc_ density ≈ 6 mA cm^–2^, and power density ≈ 22 µW cm^–2^ with prolonged durability up to 900 00 cycles. Different conditions like bending, pressure, compression, frequency, etc., could operate the PENG because of the efficient stress confinement effect. The extensional force exerted on the electrospinning jets, mechanical stretching, and interactions of Pt NPs with PVDF chains resulted in enhanced performance.

Kar et al. illustrated exceptional output power from a PENG consisting of a self‐polarized SnO_2_ nanosheet/PVDF nanocomposite.^[^ ^343^ ^]^ At an optimum SnO_2_ loading of 5 wt%, the nanocomposite possessed the highest d_33_ of ≈36.52 pC N^–1^ and F(*β*) of 74%. The PENG produced an outstanding voltage of 42 V and *j* = 6.25 µA cm^–2^ on human finger impartation. The obtained power density was 4900 W m^–3^ at an efficiency (*η*) of 16.3%. The homogeneous distribution of SnO_2_ nanosheets with large surface area enhanced *β* phase amount in PVDF and attributed to improved PENG performance. As a result of the hydrophobic nature of SnO_2_ nanosheet/PVDF composite, the PENG was able to self‐clean itself, making the device water and dirt resistive.

**Figure 25:**
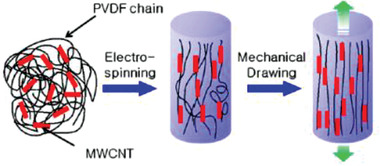
Schematic image showing the proposed mechanism of chain extension in PVDF induced by electrospinning and mechanical drawing. Reproduced with permission.^[^ ^337^ ^]^ Copyright 2013, American Chemical Society.

Ahn et al. researched the effect of varying MWCNT content (0.05–1 wt%) along with further usage of drawing and poling on piezoelectric properties of PVDF/MWCNT nanocomposites.^[^ ^337^ ^]^ The electrospun nanocomposites showed a high *β* phase amount as a result of straining the PVDF jets excessively. The long MWCNTs of 0.2 wt% acted as nuclei during the crystallization process and poling induced aggregation of charges at PVDF/MWCNT interfaces. Both of these processes promoted *α* to *β* phase conversion as shown in **Figure** **25**.^[^ ^344^ ^]^ Application of external stresses via drawing resulted in interactions between the functional groups present in MWCNTs and CF_2_ dipoles of PVDF further increasing *β*‐phase content (Figure 25). A similar study was done by Kim et al.,^[^ ^263^ ^]^ showing enhanced *β* phase content in PVDF/MWCNT composites after drawing and poling when MWCNT concentration was less than 0.2 wt%.

Liu et al. studied PVDF/MWCNT composite nanofibers synthesized by NFES.^[^ ^345^ ^]^ Application of a higher electric field of 1200 V mm^–1^ increased *β* crystallinity of PVDF fibers by roughly 53% in comparison to an electric field of 600 V mm^–1^. The d_33_ value of PVDF/MWCNT‐0.03 wt% nanofibers were −57.6 pC N^–1[^ ^203^ ^]^ compared to −15 pC N^–1^ for PVDF thin films.^[^ ^346^ ^]^ Yu et al. constructed an electrospun PVDF/MWCNTs based PENG without poling.^[^ ^347^ ^]^ The output voltage generated by the PENG was 6 V at an optimum MWCNT concentration of 5 wt%, which is 200% higher than neat PVDF nanofibers. The reason being the enhanced surface conductivity of the PVDF nanofibers caused by the addition of MWCNTs.

Wu et al. explored the influence of CNTs and optimized electrospinning parameters on the piezoelectric performance of aligned PVDF/CNT nanofibers.^[^ ^348^ ^]^ Electrospun PVDF fibers displayed F(*β*) of 88% and d_33_ value of 27.4 pC N^–1^; 18 wt% CNT loading increased F(*β*) to 89% and d_33_ to 31.3 pC N^–1^. In bending experiments, a nonlinear trend was observed for output voltage in response to the bending angle; the highest voltage being 1.89 V at 100° bend. Ning et al. prepared PVDF/MWCNT (0–0.3 wt%) nanocomposites by solution casting, with subsequent drawing to 400–500% elongation and stepwise poling to induce *β*‐phase.^[^ ^262^ ^]^ The nanocomposite film consisting of 0.05 wt% MWCNT generated twice the output voltage of pure PVDF film.

**Figure 26:**
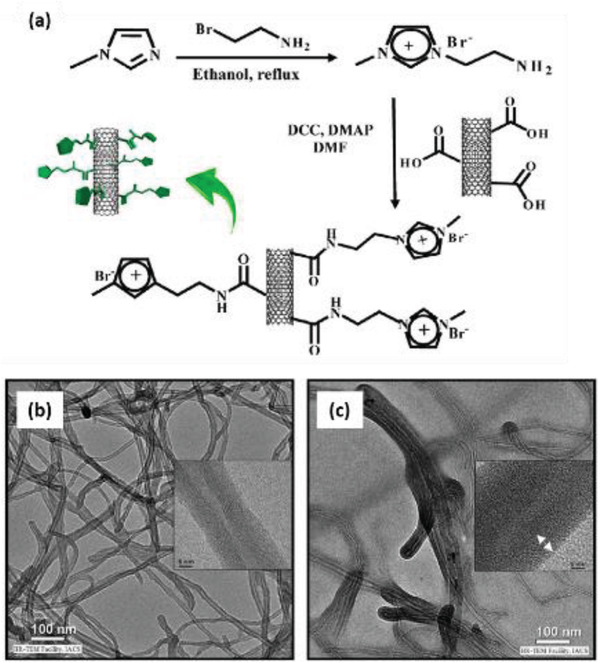
a) Schematic diagram of MWCNTs functionalization by ionic liquid to form MWCNT‐IL. TEM images of b) pristine MWCNTs and c) IL‐functionalized MCWNTs. Reproduced with permission.^[^ ^349^ ^]^ Copyright 2013, American Chemical Society.

Mandal and Nandi functionalized MWCNTs with an ionic liquid (IL, 3‐aminoethyl imidazolium bromide) to form IL functionalized MWCNTs (MWCNT‐IL) (**Figure** **26**a) and added them into PVDF.^[^ ^349^ ^]^ The average coating thickness of IL on MWCNTs was about 10 nm. The pristine MWCNTs were entangled with each other, whereas MWCNT‐IL were well dispersed, as shown by TEM images in Figure 26b,c. Nanocomposites with 100% *β*‐phase were obtained using 1 wt% MWCNT‐IL via both solvent casting and melt blending. The study showed MWCNT‐IL as an effective agent for nucleating *β*‐phase in PVDF because of dipolar interactions of PVDF with MWCNT‐IL. The covalently bonded IL helped to distribute the MWCNTs homogeneously in the PVDF matrix as well as enabled PVDF chains to embrace the TTTT conformations, leading to crystallization of *β*‐PVDF.

**Figure 27:**
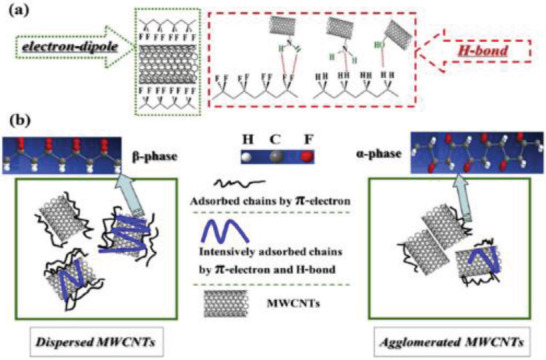
Schematic reflecting the role of CNTs on *β* phase formation in PVDF. a) The chemical bonding between functionalized CNTs and PVDF chains. b) The adsorbed PVDF chains on the CNT surface influenced by the dispersion of CNTs. Reproduced with permission.^[^ ^350^ ^]^ Copyright 2013, Elsevier Ltd.

Ke et al. prepared PVDF nanocomposites with different surface‐functionalized MWCNTs by melt mixing.^[^ ^350^ ^]^ The length of MWCNTs was ≈1 µm with an approximate outer diameter of 9.5 nm. The nonfunctionalized CNTs and those functionalized with amino, carboxyl, and hydroxyl groups were named u‐CNT, a‐CNT, c‐CNT, and h‐CNT, respectively. These four types of CNTs with 1.5, 3, and 5 wt% loadings of each were dispersed in PVDF. The maximum *β*‐phase of 17.4% was observed in nanocomposites containing a‐CNTs, followed by h‐CNTs (11.6%), u‐CNTs (9.4%), and lastly c‐CNTs (4.7%). The combined effects of MWCNT distribution in PVDF and their interactions resulted in *β*‐phase formation. These interactions were caused by excessive melt shearing along with chemical bonding between moieties present on the surface of CNTs and PVDF as shown in **Figure** **27**a. As a result of stronger electronegativity in F_2_ atoms in comparison to C and H_2_ atoms, the —CF_2_ dipoles interacted with the CNT interface having an abundance of *π*‐electrons and formed PVDF chains easily with TT conformation. The u‐CNTs, a‐CNTs, and h‐CNTs exhibited good dispersion in PVDF, resulting in PVDF adsorption along the CNT surface (Figure 27b).  

Sharma et al. showed that both fillers—carboxyl functionalized MWCNTs and Ag decorated CNTs facilitated *α* to *β* phase transition in PVDF.^[^ ^351^ ^]^ *β* phase formation was heavily dependent on the synthesis technique, that is, the electrospun fibers had a higher *β* phase than the melt mixed samples. This was ascribed to the large electrostatic field in electrospinning that aligned the PVDF dipoles in one direction. The Ag‐CNTs/PVDF fibers displayed the largest d_33_ value of 54 pC N^–1^ compared to 35 pC N^–1^ for PVDF/CNT fibers and 30 pC N^–1^ for neat PVDF.

He et al. pretreated MWCNTs using an unzipping process before adding them into the PVDF matrix, to solve the agglomeration problem of MWCNTs.^[^ ^352^ ^]^ The amounts of potassium manganate (KMnO_4_) oxidant^[^ ^353^ ^]^ were regulated to achieve MWCNTs with various unzipping degrees and the unzipped MWCNTs were named as *μ*CNTs. *β* phase was highly enhanced in PVDF nanocomposites with 0.3 wt% *μ*CNTs having a higher unzipping degree, implied by the increased d_33_ constant of 38.4 pC N^–1^. The increased interface area facilitated interactions between the oxygen‐containing groups (>C = O) of *μ*CNTs and —CF_2_ groups of PVDF, resulting in improved distribution of *μ*CNTs in PVDF matrix.

**Figure 28:**
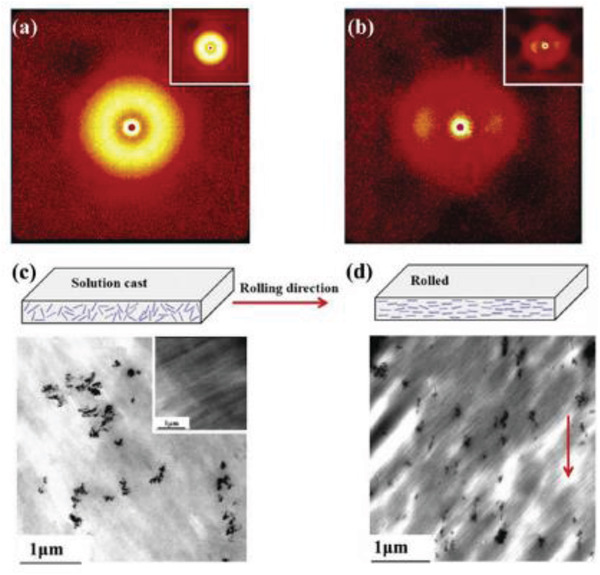
SAXS patterns of a) solution cast, b) rolled pure PVDF (inset) and composites with 0.7 wt% TiO_2_@MWCNTs loading. TEM images of c) solution cast and d) rolled composites with 0.7 wt% loading, wherein the arrow points to the rolling direction. The inset in (c) represents the TEM image of the solution cast pure PVDF. Reproduced with permission.^[^ ^354^ ^]^ Copyright 2016, Elsevier Ltd.

Yang et al. prepared nanocomposites consisting of MWCNTs coated with TiO_2_ NPs (TiO_2_@MWCNTs) and PVDF by solution casting followed by mechanical rolling.^[^ ^354^ ^]^ **Figure** **28** shows 2D small‐angle X‐ray scattering patterns for solution cast and rolled composites with 0.7 wt% loadings. A distinct isotropic ring was noticed for the solution cast samples, indicating the presence of randomly distributed lamellae (Figure 28a). In the rolled samples, two blob‐like reflections were noticed, indicating the presence of an aligned lamellar structure orthogonal to the direction of rolling (Figure 28b). The TiO_2_@MWCNTs NPs were irregularly distributed in the solution cast composites (Figure 28c). In the case of rolled composites, it can be seen clearly that rolling resulted in the orientation of the TiO_2_@ MWCNTs and increased distance between the NPs (Figure 28d). The 0.3 wt% TiO_2_@MWCNTs rolled composites displayed maximum F(*β*) ≈ 99% and d_33_ ≈ 41 pC N^–1^ after poling, which is almost twice of pure PVDF. Rolling reduced crystallite size increased phase crystallinity and formed a highly oriented structure that improved the piezoelectric response of the nanocomposites.

Baur et al. made PVDF composites with Buckminster fullerenes (C_60_) and SWCNTs over a range of compositions.^[^ ^355^ ^]^ C_60_ fullerenes consist of an sp^2^ hybridized structure with a resemblance to CNTs but are spherical in contrary to the tubular structure of CNTs.^[^ ^356^ ^]^ The SWCNT and C_60_ composites showed a maximum d_31_ of 65 and 63 pC N^–1^ at a concentration of 0.05 and 0.2 wt%, respectively. Pristine PVDF films showed a d_31_ value of 32 pC N^–1^ indicating that composites possessed a d_31_ value twice that of pristine PVDF. This was achieved through a combination of multiple factors involving nucleation of crystalline sites, internal charge creation, increased polarization, and optimization of Young's modulus and dielectric constant.

Wu et al. prepared P(VDF‐TrFE)/graphene composites and showed that 0.15 wt% graphene yielded a maximum *V*_OC_ of 12.43 V; nearly two times of pure P(VDF‐TrFE) films (6.10 V).^[^ ^357^ ^]^ Similarly, there was an increase in power density by 302% for AC circuit and 359% for DC circuit. The addition of a very low graphene amount had a negligible impact on phase formation during initial crystallization. Also, the primary factor in increasing the degree of crystallinity of the composite films was stretching; crystallinity was 40% for untreated films versus 60% for the stretched films. Abolhasani et al. prepared graphene/PVDF composite nanofibers via electrospinning and demonstrated that adding a small quantity of graphene significantly increased F(*β*).^[^ ^358^ ^]^ The *V*_oc_ and *I*_sc_ with pristine PVDF nanofibers were 3.8 V and 2 µA, which increased to 7.9 V and 4.5 µA for 0.1 wt% graphene/PVDF nanofibers. The addition of graphene beyond 0.1 wt% decreased both F(*β*) and voltage output of the randomly oriented nanofibers. It was concluded that output voltage had a complicated relationship with several factors such as F(*β*), crystallinity, diameter, and conductivity of nanofibers. Bidsorkhi et al. showed that the addition of 2 wt% graphene nanoplatelets ensured good adhesion and dispersion in the PVDF matrix and had the highest sensitivity to induced strain.^[^ ^359^ ^]^

Zhang et al. prepared graphite nanosheets (GNS)/PVDF nanocomposites consisting of different GNS volumes (1–7 mL) via solution casting.^[^ ^360^ ^]^ GNS acted as an efficient nucleating agent and the content of the *β* phase in PVDF increased by the addition of a higher amount of GNS. The d_33_ value reached a maximum of 6.7 pC N^–1^ using 6 ml GNS. The TTTT conformation chains in PVDF were tied to the GNS surface, therefore, under application of electric field to the nanocomposites, the H_2_ and F_2_ atoms easily rotated inducing electrical dipoles.

**Figure 29:**
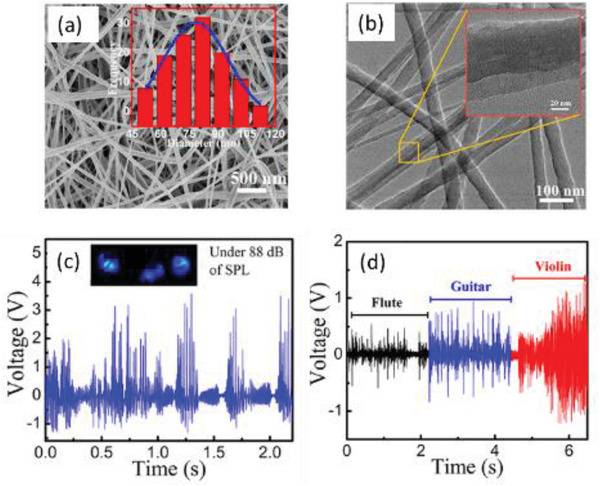
a) FE‐SEM image of PVDF/Ce‐graphene nanofibers. The inset shows the statistical size distribution of the NFs. b) TEM image and higher‐resolution image (inset) of PVDF/Ce‐graphene NFs. c) The output voltage from the PENG driven by the music of 88 dB intensity. Instantaneous lighting of 3 blue LEDs shown in the inset. d) Voltages from various instruments‐flute, guitar and violin of the National anthem of India. Reproduced with permission.^[^ ^361^ ^]^ Copyright 2016, American Chemical Society.

Garain et al. reported a flexible pressure sensor with in situ poling capability by using electrospun Ce^3+^ doped graphene/PVDF composite nanofibers.^[^ ^361^ ^]^ PVDF, Ce^3+^ and graphene were used at a concentration of 12, 0.2, and 1 wt% to obtain randomly oriented nanofibers of ≈80 nm diameter (**Figure** **29**a,b). The interactions between Ce^3+^ and —CH_2_—/—CF_2_— dipoles of PVDF along with the interactions between —CH_2_— dipoles and delocalized *π*‐electrons in graphene resulted in F(*β*) of ≈99%. The sensor could successfully detect low imparting pressures with a very high degree of sensitivity. The PENG yielded an output voltage of 11 V, *j* = 6 nA cm^–2^ and a maximum power of 6.8 µW under the application of 6.6 kPa pressure. It successfully harvested sound energy of ≈88 dB intensity, generating an AC output of ≈3 V (Figure 29c) and from music played on various instruments like flute, guitar and violin (Figure 29d), making it suitable to perform as an acoustic NG.

Sinha et al. constructed a PENG with graphene‐silver (GAg) doped PVDF nanocomposites, prepared using a facile, one‐pot method.^[^ ^362^ ^]^ GAg was made by incorporating Ag‐NPs in graphene; this resulted in n‐type doping of graphene due to electrostatic interaction. These interactions led to polar GAg formation, inducing PVDF self‐polarization and favoring *β*‐phase nucleation. The PENG was able to detect visible light because of the existence of photo‐sensitive plasmonic GAg, resulting in an enhanced *η* value of up to 46.6%. These results showed that the PENG could be used in self‐powered visible light sensors in flexible optoelectronics.

Layek et al. formulated PMMA‐functionalized graphene (MG) using atom transfer radical polymerization and incorporated it into PVDF.^[^ ^261^ ^]^ PMMA increased the height of the graphene sheet from 1.2 to 2 and 4.5 nm due to MMA polymerization at basal and side planes. MG sheets at 5 wt% loading nucleated *β*‐PVDF crystals, hence a subsequent decline in the *α* phase was observed in the nanocomposites with a resultant increase of *β* phase. Barstugan et al. electrospun nanocomposite fibers from a solution containing polybenzoxazole (PBO), PVDF, and graphene.^[^ ^363^ ^]^ The addition of 1 wt% PBO and 0.3 wt% graphenes increased the piezoelectric effect and thermal strength of PVDF. The voltage generated by the PBO/graphene/PVDF fibers increased when they were thinner, were subjected to higher pressure and were electrospun on the copper plate instead of aluminum foil. The fibers with 0.02 and 0.06 mm diameter generated maximum voltage of 60 and 9.68 V.

Alamusi et al. prepared reduced graphene oxide (rGO)/PVDF nanocomposites with rGO content in the range of 0–0.2 wt% via solution casting.^[^ ^338^ ^]^ The nanocomposite films were drawn up to 400–500% at a rate of 10 mm min^–1^ and polarized by stepwise poling (60 MV m^–1^ at 8 min intervals) to induce *β* phase. The rGO/PVDF films containing 0.05 wt% rGO displayed a peak output voltage of 3.28V; around 293% of pure PVDF film. The main reason for the enhanced output was the formation of a higher *β* phase amount at 0.05 wt% rGO concentration, as affirmed by XRD and FTIR analyses. Rahman et al. synthesized rGO/PVDF nanocomposites via thermal reduction of graphene oxide/PVDF films at 0.1 wt% GO loading.^[^ ^364^ ^]^ The PENG scavenged a maximum power of 36 nW across a resistance of 704 kΩ, which was better than pure PVDF. The conductive rGO nanofillers induced heterogeneous polarization in the PVDF matrix which led to improved properties. Later, he reported 100% *β* phase formation in PVDF at 0.3 wt% rGO loading.^[^ ^365^ ^]^ Kumar et al. dispersed rGO in PVDF with varying amounts (0.1–1.0 wt%) and showed that both *β* and *γ* phases were induced in the nanocomposites.^[^ ^366^ ^]^ The maximum output voltage and current obtained from the PENG was 0.45 V and 150 nA at 1 wt% rGO under a load of 500 gm; five times greater than pure PVDF. The long‐term stability of the device was demonstrated by testing over 2000 cycles at high stress. In 2018, Habibur et al. investigated the impact of varying rGO contents on *β* phase formation in P(VDF‐TrFE).^[^ ^367^ ^]^ At 0.1 wt% rGO loading, a maximum *β* phase amount of ≈90% and d_33_ value of −23 pC N^–1^ was achieved from the nanocomposites. The PENG, without poling, produced a maximum *V*_OC_ of 2.4 V and *I*_SC_ ≈ 0.8 µA when 2 N force was applied. The maximum output power was 3.2 µW across 1.8 MΩ load resistance.

**Figure 30:**
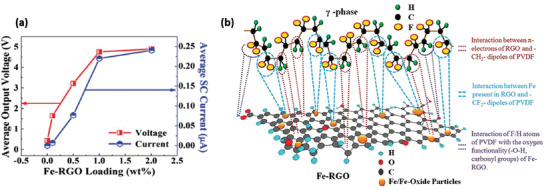
a) Output voltage and current are shown as a function of Fe‐rGO filler loading in PVDF. b) Proposed schematic showing the interactions between *γ*‐phase in PVDF and a Fe‐rGO nanosheet (by assuming a single *γ*‐phase and Fe‐RGO sheet). Reproduced with permission.^[^ ^368^ ^]^ Copyright 2015, The Royal Society of Chemistry.

Karan et al. reported a PEH using non‐poled Fe‐doped rGO/PVDF nanocomposites.^[^ ^373^ ^]^ The nanocomposites were prepared by solution casting which enabled nucleation of ≈ 99% polar *γ* phase. The PEH with 2 wt% Fe‐rGO loading generated a maximum *V*_OC_ of 5.1 V and *I*_SC_ of 0.254 µA as a result of continuous imparting by fingers (**Figure** **30**a). The improved output was because of electrostatic interactions between —CH_2_—/—CF_2_— dipoles in PVDF, delocalized *π*‐electrons, and oxygen functional groups in Fe‐doped rGO through ion‐dipole and hydrogen bonding interactions as shown in Figure 30b. Similarly, Pusty et al. showed that the addition of Ag‐doped rGO enhances both *β* and *γ* phases in PVDF without external poling.^[^ ^374^ ^]^ The PENG with 1 wt% Ag‐rGO showed the highest *V*_OC_ of 18 V and *I*_SC_ of 1.05 µA, corresponding to a peak power density of 28 Wm^–3^ across a 1 MΩ resistor. It was able to successfully harvest energy from fingers on human palm and foot‐tapping when attached to flip flops.

**Figure 31:**
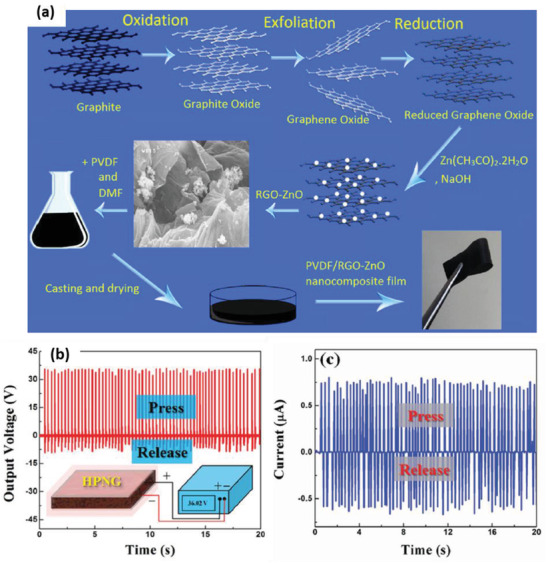
a) The mechanism of PVDF/rGO‐ZnO nanocomposite films production. Reproduced with permission.^[^ ^370^ ^]^ Copyright 2014, Elsevier B.V. b) Output voltage and c) current generated from 1 wt% AlO‐rGO/PVDF PENG. b,c) Reproduced with permission.^[^ ^371^ ^]^ Copyright 2016, Wiley‐VCH.

Jaleh and Jabbari prepared PVDF/rGO‐ZnO nanocomposites using various weight ratios of rGO‐ZnO (1:1, 2:1, 4:1) by solution casting and added to PVDF at 1 wt% concentration as shown in **Figure** **31**a.^[^ ^370^ ^]^ For the PVDF/rGO‐ZnO (4:1) nanocomposite film, the maximum *β*‐phase obtained was 83%. Reduced size of spherulites was observed in the crystal structure of PVDF/rGO‐ZnO nanocomposites in comparison to pristine PVDF, hence it had an explicit effect on *β* phase increment. Karan et al. reported a PENG using a nanocomposite of aluminum oxide (AlO) doped rGO/PVDF.^[^ ^371^ ^]^ Incorporation of 1 wt% AlO‐rGO increased *β*‐phase proportion up to ≈90% in addition to a small proportion of *γ*‐phase (≈3%) and the measured d_33_ value was ≈45 pC N^–1^. The PENG exhibited excellent performance without poling (*V*_OC_ ≈ 36 V, *I*_SC_ ≈ 0.8 µA) (Figure 31b,c) due to the formation of polar *β*‐phase through polarization induced by surface charges and external stress. Roy and Mandal showed that 0.25 wt% of cadmium sulfide (CdS) doped rGO induced more than 90% *β* phase in electrospun PVDF nanofibers.^[^ ^372^ ^]^ The PENG produced a voltage of about 4 V on continuous human finger impartation.

Pusty et al. presented a comparative study of energy harvesting performance between CNT/PVDF and Fe‐rGO/PVDF nanocomposites.^[^ ^373^ ^]^ The PEH had a unique structure, in which the bottom electrode was not in contact with the composite films until the external force was applied. It generated a *V*_OC_ of 2.5 V and *I*_SC_ of 700 nA for CNT/PVDF and 1.2 V, 300 nA for Fe‐rGO/PVDF nanocomposites, respectively. The reason behind higher electrical response in CNT/PVDF nanocomposite was that it stabilized the *β* and *γ* phases to a greater extent than Fe‐rGO/PVDF and pure PVDF.

Achaby et al. made graphene oxide nanosheets (GOn)/PVDF nanocomposites exhibiting *β*‐phase at a low GOn amount of 0.1 wt%, below which a combination of *α* and *β*‐phases were seen.^[^ ^374^ ^]^ Graphite oxide was synthesized from natural graphite via Hummer's method.^[^ ^375^, ^376^ ^]^ It which was then dispersed in DMF and ultrasonicated to synthesize GOn. The functional groups in GO interacted with —CF_2_/—CH_2_— groups in PVDF and promoted *β* phase crystallization in the nanocomposites. Jiang et al. showed pure *β* phase formation in GO/PVDF nanocomposites after uniaxial stretching, with much lower plastic strain than that of PVDF at the same temperature.^[^ ^377^ ^]^

Gebrekrstos et al. functionalized GO with carboxylated and fluorinated derivatives (GOCOOH and GOF) and then prepared PVDF/GO, PVDF/GOCOOH, and PVDF/GOF fibers via electrospinning.^[^ ^378^ ^]^ The *β*‐phase quantities for PVDF/GO, PVDF/GOCOOH, and PVDF/GOF were 69%, 79%, and 89% and d_33_ values were 40, 46, and 63 pC N^–1^, respectively. These were much higher than pure PVDF that exhibited 38% *β* phase and d_33_ of 30 pC N^–1^.^[^ ^356^ ^]^ These enhancements were due to the uniaxial straining of the nanocomposites by electrospinning that promoted *α* to *β*‐phase transformation via the assistance of graphene derivatives. Among the nanocomposites, the highest *β*‐phase/d_33_ was obtained in the presence of GOF because of the existence of highly electronegative fluorine.

Wu et al. used amorphous carbon black (CB) as a nanofiller because of its substantially high surface area compared to CNTs and easier dispersion in comparison to graphene.^[^ ^264^ ^]^ CB content of 0.5 wt% reduced crystal size and augmented the amount of crystals in P(VDF‐HFP)/CB nanocomposite. This favored the formation of elongated and aligned crystal morphology in the course of stretching and resulted in very efficient poling. The calibrated Voc of the nanocomposite films at an optimum CB concentration of 0.5 wt% was 104% of pristine P(VDF‐HFP). The power density of pure P(VDF‐HFP) films was 6.1 W m^–3^, while for the P(VDF‐HFP)/CB system it increased to 28.3 Wm^–3^ which was ≈464% of pure P(VDF‐HFP).

**Figure 32:**
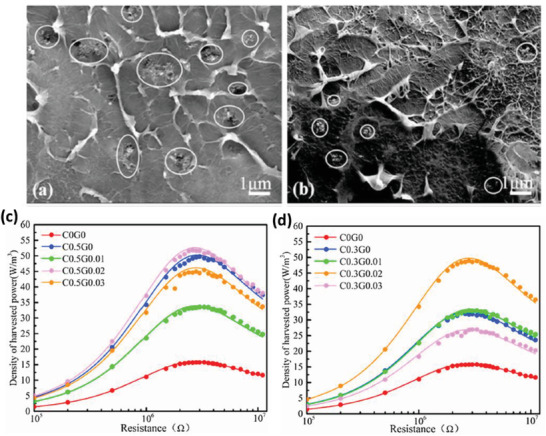
Cross‐sectional SEM image of a) CB‐0.5/P(VDF‐HFP) composite film b) CB‐0.5FLG‐0.03 composite film. The particles circled in (a) and (b) shows the nanofillers. Harvested power density of c) 0.3 wt% CB and different FLG loadings. d) 0.5 wt% CB and different FLG loadings. Reproduced with permission.^[^ ^265^ ^]^ Copyright 2019, Elsevier Ltd.

Cai et al. prepared P(VDF‐HFP) nanocomposites with CB and few‐layer graphene (FLG), showing that *β*‐phase fraction increased significantly by modifying the quantities of CB and FLG.^[^ ^265^ ^]^ The *β*‐phase fraction improved from 27% for pure P(VDF‐HFP) to 98% for CB‐0.3/FLG‐0.02 wt% and 99% for CB‐0.5/FLG‐0.02 wt% films after poling and stretching. The fracture surface of CB/FLG/P(VDF‐HFP) composite films is shown by SEM images in **Figure** **32**a,b. In the films which contained CB only, agglomeration of CB was seen (Figure 32a). The addition of FLG to the nanocomposites reduced CB agglomeration and resulted in uniform distribution of the hybrid fillers (Figure 32b). The maximum harvested power density of 51.9 W m^–3^ was obtained from CB‐0.5/FLG‐0.02 wt% films which was much higher compared to 15.8 W m^–3^ for P(VDF‐HFP) films (Figure 32c). The second‐maximum power density was obtained from CB‐0.3/FLG‐0.02 wt% films (Figure 32d) which is consistent with open‐circuit voltage and *β* phase fraction. Enhanced piezoelectricity of the nanocomposites was ascribed to two factors‐ improved nucleating effect of CB/FLG nanofillers due to their uniform dispersion in P(VDF‐HFP) matrix and increased conductivity of the nanofiller network due to addition of FLG.

Based on summarized literature in this section, it can be observed that the addition of conducting fillers to PVDF results in lower output performance compared to the addition of non‐conducting fillers. This was primarily due to lower piezoelectric coefficients of conducting fillers than non‐conducting fillers. Generally speaking, poling was not done in nanocomposites where Ag NPs, Pt NPs, metal‐doped rGO, and graphene oxide were used as fillers. Whereas nanocomposites with fillers such as MWCNTs, CB NPs, and rGO underwent poling treatment. The filler content in these nanocomposites varied between 0 and 18 wt% which was much lower than that of non‐conducting fillers. Nanocomposite synthesis methods included electrospinning (regular and NFES), solution casting, drop‐casting, and ultrasonication with hot pressing/centrifugation. Moreover, the output performance was highly dependent on the type of filler used, their concentration, the surface modifying agents of the fillers and synthesis conditions. The maximum output voltage of 60 V was obtained from electrospun PVDF and 0.3 wt% graphene treated PBO nanocomposites in the absence of poling.^[^ ^363^ ^]^

#### 5.1.3 Nanocomposites Based on Conducting and Non‐Conducting Filler Combination

This section focuses on PVDF nanocomposites synthesized using a combination of conducting and non‐conducting fillers and hybrid fillers such as, NaNbO_3_‐reduced graphene oxide (rGO),^[^ ^266^ ^]^ titanium oxide (TiO_2_)‐rGO nanotubes,^[^ ^267^ ^]^ and manganese oxide (MnO_2_)/graphene/MWCNT hybrids.^[^ ^268^ ^]^ We review their fabrication techniques, piezoelectric properties, and energy harvesting performance of PENGs.

Kim et al. prepared nanocomposites with various amounts of MWCNTs and BaTiO_3_ NPs by fused deposition modeling (FDM) 3D printing process for flexible sensor applications.^[^ ^379^ ^]^ MWCNTs were added in the PVDF matrix to give rise to a stress reinforcing network and electron conduction path as well as to act as a dispersant for BaTiO_3_ NPs. The highest d_31_ was 0.13 pC N^–1^ at 0.4 wt% MWCNTs/18 wt% BaTiO_3_. The measured output voltage was ±120 mV upon bending and ±435 mV upon pressing the nanocomposite sensor by human fingers. Increasing MWCNT content also resulted in higher output due to the stress reinforcing effect between BaTiO_3_ and PVDF.

Ponnamma and Maadeed prepared a nanocomposite by dispersing BaTiO_3_ NPs and hexagonal boron nitride (h‐BN) nanolayers in the P(VDF‐HFP) matrix.^[^ ^380^ ^]^ In comparison to the individual P(VDF‐HFP)/BaTiO_3_ and P(VDF‐HFP)/h‐BN nanocomposites, the hybrid composite containing 3 wt% BaTiO_3_ and 1 wt% h‐BN displayed exceptional performance with an output voltage of 2.4 V. The synergistic effect of fillers achieved through interactions between BaTiO_3_ NPs and h‐BN nanolayers led to improved performance in the nanocomposites.

**Figure 33:**
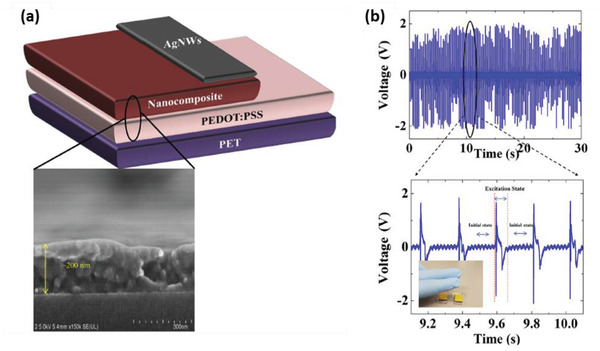
a) Schematic diagram of the fabricated PENG device. The inset is a cross‐sectional SEM image of the PENG. b) *V*_oc_ measurements of the PENG in transverse (d_33_) mode with a magnified view of the selected region. The inset in the magnified view illustrates the d_33_ mode generator. Reproduced with permission.^[^ ^381^ ^]^ Copyright 2018, Elsevier B.V.

Bakar et al. fabricated an ITO‐free PENG consisting of a nanocomposite of BaTiO_3_, graphene quantum dots (GQDs) and PVDF.^[^ ^381^ ^]^ GQDs were introduced as nanofillers because of their distinctive optical, electronic, spintronic and photoelectric properties induced by quantum confinement effect, and edge effect. The commonly used ITO bottom electrode was replaced by PEDOT: PSS conductive layer because it was thought to be durable enough to operate at higher frequencies of 1 MHz (**Figure** **33**a). The GQDs enhanced Vp‐p of the PENG to 4.6 V in d_33_ mode (Figure 33b) due to the “charges trapped effect” at GQD/PVDF interface. The PENG was able to operate a LED by connecting to an external circuit and exhibited stability up to 60 h without significant degradation.

A pressure sensor was assembled by Saygh et al. using a PVDF nanocomposite of TiO_2_ nanolayers (TNL) and rGO, containing 2.5 wt% each.^[^ ^382^ ^]^ The TNL/rGO fillers were well scattered in the PVDF matrix and interacted effectively with it. The sensitivity of the pressure sensor increased by 333.46% at 5 kPa, 200.7% at 10.7 kPa, and 246.7% at 17.6 kPa in comparison to the nanocomposites of PVDF and TNL only. The increased amount of *β* crystallinity and *α* phase removal was observed in the TNL/rGO/PVDF nanocomposite which was linked to advancement in mechanical properties of the sensor. Ponnamma et al. measured the gas sensing properties and energy harvesting capabilities of TiO_2_ nanotubes/CNT/PVDF nanocomposites with varying filler concentrations.^[^ ^383^ ^]^ The gas sensor with 2.5 wt% PVDF/TiO_2_‐CNT showed a sensing response of 0.45 s upon exposure to 400 ppm LPG, which was about nine times higher than the composite containing 2.5 wt% of TiO_2_ or 2.5 wt% CNT. The output voltage was ≈0.3–0.8 V for the same filler concentration due to the uniform distribution of TiO_2_/CNT nanotubes within PVDF, resulting in high *β* phase content.

Ponnamma et al. introduced a combination of 1D TiO_2_ nanotubes, 2D rGO and 3D strontium titanate (SrTiO_3_) into the P(VDF‐HFP) matrix followed by poling.^[^ ^267^ ^]^ The TiO_2_ nanotubes were hydrothermally grown in the presence of rGO to obtain a nanosheet‐nanotube hybrid structure.^[^ ^384^ ^]^ The d_33_ coefficient attained a maximum value of 7.52 pC N^–1^ and a peak output voltage of 1 V was obtained when rGO‐TiO_2_/SrTiO_3_ was 1:2 by wt% at 45 Hz vibration frequency. The output voltage was about 10.5 times greater than the voltage produced by neat P(VDF‐HFP). An increase in filler concentration resulted in non‐uniform dispersion and reduced performance output.

Singh et al. fabricated NaNbO_3_/rGO/PVDF, rGO/PVDF, and NaNbO_3_/PVDF films to understand the impact of adding NaNbO_3_ and rGO fillers in PVDF.^[^ ^266^ ^]^ Although *β* phase content was similar in the three nanocomposites, the output generated from NaNbO_3_/rGO/PVDF based PENG was the highest (*V*_oc_ ≈ 2.16 V, *I*_SC_ ≈ 0.383 µA). The NaNbO_3_ nanorods with their inherent piezoelectric property helped to align the dipoles in PVDF. The presence of rGO helped to provide a conductive path to the charges produced within the film which further allowed the PVDF dipoles to align and contributed to the enhanced performance.

Samadi et al. synthesized PVDF nanocomposites with iron oxide‐graphene oxide (Fe_3_O_4_‐GO) nanoparticle‐nanosheets.^[^ ^385^ ^]^ Fe_3_O_4_‐GO/PVDF nanocomposite fibers with various ratios of Fe_3_O_4_‐GO (0, 1, 1.5, and 2 wt%) were produced by solution mixing and electrospinning. The addition of GOn to PVDF increased the conductivity of the polymer solution due to their high surface charge density. The magnetic Fe_3_O_4_ NPs increased the crystallinity of PVDF and *β*‐phase content. Fe_3_O_4_‐GO/PVDF nanofibers exhibited good piezoelectric properties owing to the combination of electrospinning and the presence of magnetic Fe_3_O_4_ NPs and conductive GOn. In 2019, the same group synthesized TiO_2_‐Fe_3_O_4_‐MWCNT/PVDF nanocomposite for achieving the synchronistic effect of piezoelectric, magnetic and conductive properties of TiO_2_, Fe_3_O_4_ and MWCNTs respectively.^[^ ^386^ ^]^ Composite nanofibers with different ratios of TiO_2_‐Fe_3_O_4_‐MWCNT nanotubes (0–2 wt%) were prepared via electrospinning to obtain a higher *α* to *β* phase transformation. Piezoelectric sensitivity, defined as output voltage/applied force was highest for 2 wt% nanocomposites (51.42 mV N^–1^) and lowest for neat PVDF sample (26.95 mV N^–1^). The incorporation of hybrid nanotubes into PVDF promoted crystallinity and *α* to *β* phase transformation which resulted in improved piezoelectric properties.

**Figure 34:**
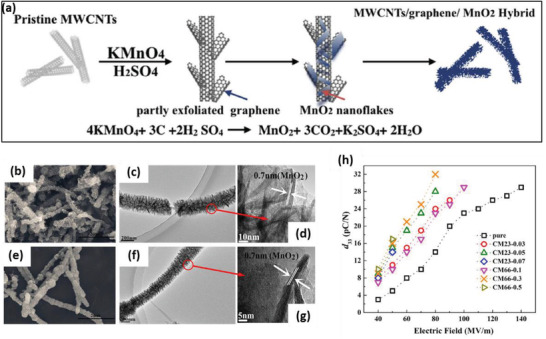
a) The synthesis route of the MWCNTs/graphene/MnO_2_ hybrid. SEM, TEM, and high‐resolution TEM images of b–d) CM23 and e–g) CM66 hybrids. h) Variation of piezoelectric coefficients d_33_ with poling electric fields in CM23 and CM66 nanocomposites. Reproduced with permission.^[^ ^268^ ^]^ Copyright 2018, Elsevier Ltd.

Yang et al. built a 3D nanostructure consisting of manganese dioxide (MnO_2_)/graphene/MWCNTs with varied MnO_2_ loadings to enhance the piezoelectricity of PVDF.^[^ ^268^ ^]^ **Figure** **34** shows the synthesis method of MnO_2_/graphene/MWCNTs hybrids. Two hybrids consisting of 23 wt% and 66 wt% MnO_2_ were fabricated and were named as CM23 and CM66, respectively. Compared to pristine MWCNTs, the CM23 and CM66 hybrids displayed improved surface area and roughness with a large number of attached —OH groups (Figure 34b–g), resulting in good compatibility with PVDF. The nanocomposites with 0.03–0.07 wt% CM23 and 0.1–0.5 wt% CM66 loading showed a d_33_ value of 17–33 pC N^–1^ after poling under an electric field of 50–80 MV m^–1^, whereas a field above 100 MV m^–1^ was required to generate similar piezoelectric performance in pure PVDF (Figure 34h). The nanocomposites containing CM23 showed higher conductivity compared to CM66, therefore it was more effective in enhancing the local electric field, which allowed for a more uniform polarization and higher piezoelectric performance.

Abbasipour et al. investigated the effect of three nanofillers, namely, GO, graphene and halloysite (Hal) nanotubes with varying concentrations (0.05–3.2 wt%) on piezoelectric properties of PVDF.^[^ ^387^ ^]^ A *β*‐phase increase of up to 49% was observed in the electrospun PVDF nanofibers compared to PVDF powders and the addition of nanofillers increased it further by 10%. Due to the weak interactions of graphene with PVDF, it had the lowest impact on the piezoelectric properties of the nanocomposites. PVDF/Hal nanocomposites with <0.1 wt% loading and PVDF/GO with >0.4 wt% loading originated higher *β* phase amount. Highly oriented and finer nanofibers were obtained from Hal nanotubes because of their rod‐like morphology when compared to PVDF/GO and PVDF/graphene nanofibers. PVDF/0.8Hal nanocomposites revealed a higher voltage output of 0.1 V, regardless of lower *β*‐phase amount than PVDF/0.8GO nanocomposites, thus proving that nanofiber orientation and fineness are important factors in addition to *β* phase content.

PMN‐PT exhibits a very high d_33_ value of ≈2500 pC N^–1^.^[^ ^388^ ^]^ Das et al. synthesized PMN‐PT/CNT/PVDF based flexible nanocomposites with 30 vol% PMN‐PT and 1 vol% CNT.^[^ ^389^ ^]^ This nanocomposite generated the largest output voltage and current of ≈4 V and 30 nA, which were higher than PVDF/CNT composites because of the high piezoelectric properties of PMN‐PT. The interactions at the interfaces of PMN‐PT and PVDF got strengthened due to the conductive network formed by the CNT particles.

Bodkhe et al. examined the effect of varying concentrations of microcrystalline cellulose, CNTs, and kaolinite clay on PVDF *β* phase formation.^[^ ^390^ ^]^ PVDF/cellulose‐1 wt% nanocomposites exhibited 82.04% *β* phase, higher than PVDF/CNT and PVDF/clay composites. This was ascribed to the unique balance between surface area and size of cellulose fibers and the presence of a large number of —OH groups on cellulose which facilitated *β*‐phase formation. At filler loading >1 wt%, *β*‐phase content in both nanocomposites decreased due to agglomeration. Detailed explanation on *β*‐phase enhancement in PVDF/clay and PVDF/MWCNT composites were reported by Yu et al. based on density functional theory^[^ ^344^ ^]^ and Flory mixing theory.^[^ ^391^ ^]^ A similar study was done by Ramasundaram et al. showing enhanced *β* phase in PVDF/modified clay nanocomposites.^[^ ^326^ ^]^

Based on the current literature, we summarize that the addition of a hybrid or mixture of conducting and non‐conducting fillers to PVDF results in lower output performance compared to the addition of these fillers separately. The filler content in these nanocomposites was in the range of 0.1–18 wt%. The non‐conducting fillers (BaTiO_3_, PMN‐PT particles, etc.) acted as the source of piezoelectric potential and the conducting fillers (MWCNTs, rGO, etc.) functioned as a dispersant to distribute the nanoparticles uniformly throughout the polymer matrix. Nanocomposite synthesis methods include electrospinning, FDM 3D printing, solution casting, and magnetic stirring with heat treatment. Again, the output performance was highly dependent on the type and concentration of filler used, the surface modifying agents of the fillers and synthesis conditions. The maximum output voltage of 4.6 V was obtained from a PENG consisting of a nanocomposite of 2 wt% BatiO_3_, 1.5 wt% GQDs and PVDF, prepared by spin casting in the absence of poling.^[^ ^381^ ^]^

### 5.2 Polydimethylsiloxane Based Nanocomposites

PDMS (CH_3_[Si(CH_3_)_2_O]*_n_* Si(CH_3_)_3_) is a polymer comprising of lengthy chains of repeating monomers [SiO(CH_3_)_2_]. It has excellent flexibility and the ability to withstand high strains, making it crucial for applications involving bending, stretching, twisting, and rolling. In this section, PDMS nanocomposites based on non‐conducting, hybrid and mixture of non‐conducting, and conducting fillers will be reviewed. There was no work reported in the literature on PDMS nanocomposites using conducting fillers, hence it has not been explained in this section.

#### 5.2.1 Nanocomposites Based on Non‐Conducting Fillers

This section focuses on piezoelectric PDMS nanocomposites in which non‐conducting fillers such as, BaTiO_3,_ ZnSO_3_, NaNBO_3_ are embedded in the polymer matrix. We discuss the concentration of fillers used, synthesis methods, piezoelectric properties, and outputs generated from energy harvesting.

**Figure 35:**
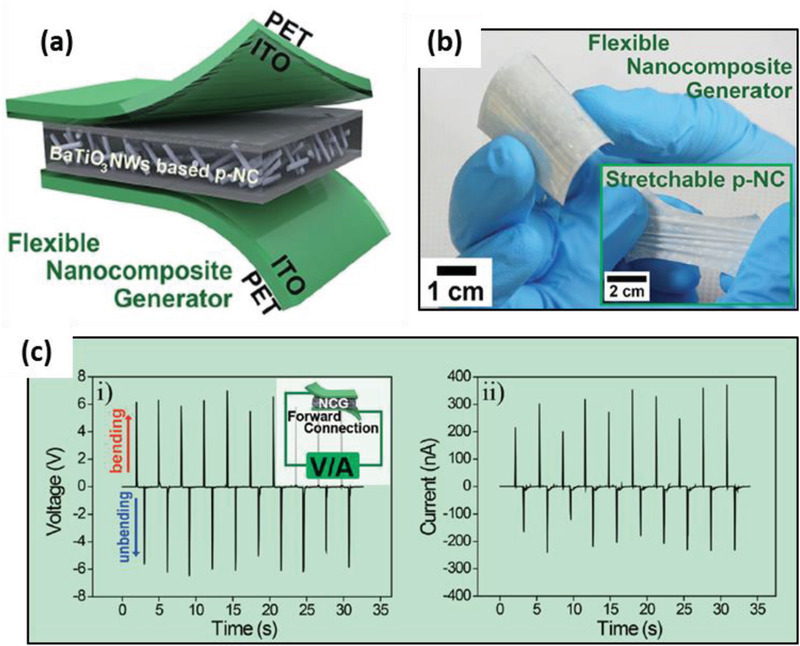
a) Schematic illustration of overall fabrication for BaTiO3 NWs‐based PENG. b) Photograph of the PENG (3 cm × 4 cm) completely bent by human fingers. The inset shows the nanocomposite layer stretched by fingers without any damage. c) The electrical signals measured from the PENG. Reproduced with permission.^[^ ^392^ ^]^ Copyright 2014, The Royal Society of Chemistry.

Park et al. prepared a nanocomposite by impregnating BaTiO_3_ NWs into the PDMS matrix without using any dispersion enhancers.^[^ ^392^ ^]^ BaTiO_3_ NWs with high aspect ratio and crystallinity were synthesized via the hydrothermal method (described in section 3). The PENG consisted of PDMS/nanocomposite/PDMS layers, sandwiched between two ITO‐coated PET substrates as shown in **Figure** **35**a. The spin‐coated, thin PDMS layers functioned as a dielectric to sustain electrical stability during poling as well as mechanical robustness. Figure 35b displays the PENG completely bent by human fingers without any damage, proving its flexible nature. During cyclic bending and unbending motions, the PENG exhibited a high output voltage and current up to 7 V and 360 nA due to the well‐distributed BaTiO_3_ NWs in the elastomeric matrix (Figure 35c). In another study, Baek et al. obtained excellent performance from a PENG by using a nanocomposite of BaTiO_3_ NPs and BaTiO_3_ NWs in PDMS without using any dispersion agents.^[^ ^393^ ^]^ The weight ratio between NPs and NWs was varied between 20:1 and 2:1 in the nanocomposites. At an optimum ratio of 4:1, the PENG produced the highest *V*_OC_ of ≈60 V and *I*_SC_ of ≈1.1 µA with an instantaneous power of 40 µW. Also, the output performance was stable up to 3000 bending and unbending cycles, which illustrates the reliability of the PENG.

Lin et al. developed a flexible PENG by using a nanocomposite of 3 wt% BaTiO_3_ nanotubes/PDMS.^[^ ^394^ ^]^ The hydrothermal process was used to synthesize BaTiO_3_ nanotubes. Under applied stress of 1 MPa, the PENG generated a voltage and current of 5.5 V and 350 nA, that drove a commercial LCD. The PENG displayed superior flexibility and prolonged stability owing to the presence of PDMS. The BaTiO_3_ nanotubes/PDMS nanocomposite possessed high transparency and were effective for the fabrication of large‐scale (11 × 11 cm) nanogenerators.

Yan and Jeong studied the piezoelectric performance of 31 wt% BaTiO_3_/PDMS nanofibers with multiple alignment modes.^[^ ^395^ ^]^ The nanofibers were synthesized by electrospinning and were aligned vertically, horizontally, or randomly in the PDMS matrix. The output voltage of the PENG reached about 0.56, 1.48, and 2.67 V for the random, horizontal, and vertical nanofibers under low compressive stress of 0.002 MPa. The PENG with vertical nanofibers achieved maximum output due to two reasons—first, efficient poling (without poling, the voltage was only 0.08 V) and second, accumulation of higher electric charges on the electrodes because of the higher density of BaTiO_3_ and compliance to mechanical stress. The output current followed the same trend as output voltage, that is, 57.78, 103.33, and 261.40 nA for the random, horizontal, and vertical nanofibers.

**Figure 36:**
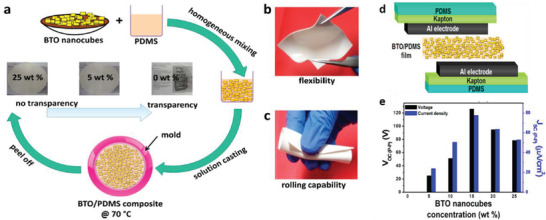
a) Fabrication of piezoelectric BaTiO_3_ nanocube/PDMS composite films using solution casting technique. The inset photographs show the transparency of the composites with various weight ratios of BaTiO_3_ NCs in the PDMS matrix. b,c) Photographs demonstrating the flexibility and rolling capabilities of the nanocomposite films. d) Schematic diagram of the PENG for harnessing mechanical energy. e) Comparison of *J*–*V* responses of the PENG as a function of BaTiO_3_ nanocube weight ratio under low mechanical pressure of 988.14 Pa. Reproduced with permission.^[^ ^396^ ^]^ Copyright 2017, American Chemical Society.

Alluri et al. prepared BaTiO_3_ nanocube/PDMS nanocomposite films via solution casting with high flexibility and rolling capabilities^[^ ^396^ ^]^ (**Figure** **36**a–c). The PENG was made by sandwiching BaTiO_3_/PDMS nanocomposite between flexible Al/Kapton films by hot pressing (Figure 36d). The PENG with 15 wt% BaTiO_3_/PDMS nanocomposite yielded a maximum *V*_oc_ of 126.3 V and *j* = 77.6 µA cm^–2^ under mechanical stress of 988.2 Pa (Figure 36e). The composites containing 20 and 25 wt% BaTiO_3_ showed lower outputs due to particle agglomeration in the PDMS matrix as shown in Figure 36e.

A flexible PENG was also reported using BaTiO_3_ nanostructure, prepared from a genetically modified virus template.^[^ ^397^ ^]^ Barium glycolate and titanium glycolate were added back‐to‐back to the virus solution and heated to 80°C to form a virus‐metal ionic complex that was calcined at 1000 °C to form a crystalline BaTiO_3_. The BaTiO_3_ nanostructure was embedded in PDMS to form a piezoelectric layer of ≈200 µm thick. The layer was sandwiched between a flexible substrate and an ITO‐coated PET electrode to fabricate the PENG. Outstanding output performance (current ≈ 300 nA and voltage ≈ 6 V) was achieved from the PENG by periodic mechanical motions, although no distinct advantage of using the virus‐templated structure was explained.

Lee et al. reported high power generation from a PENG using a nanocomposite of zinc stannate (ZnSO_3_) nanocubes and PDMS in the absence of poling.^[^ ^398^ ^]^ The PENG with 40 wt% ZnSnO_3_ generated a maximum output voltage of 12 V and *j* = 0.89 µA cm^–2^ when a compressive strain of 0.91% was vertically applied. The high output was a result of the high dielectric constant of the PENG arising from interfacial Maxwell‐Wagner‐Sillars polarization between the ZnSnO_3_ nanocubes and PDMS. The PENG operated successfully under vertical compression but generated insignificant power when the strain was applied via other configurations. This unidirectional behavior is desirable for massive‐area power generation contingent on vertical compression‐like human walking, moving vehicles and railway transport.

Shin et al. used a nanocomposite of Li‐doped ZnO NWs/PDMS for constructing a large‐sized PENG of 10 cm x 10 cm.^[^ ^399^ ^]^ Ferroelectric phase transition takes place in Li‐doped ZnO by the replacement of Zn atoms with Li atoms due to substantial differences in radii of Zn (0.74 Å) and Li (0.60 Å) atoms.^[^ ^400^ ^]^ The PENG yielded an output voltage and current of up to 180 V and 50 µA under periodic bending and releasing.

Jung et al. constructed a flexible PENG using NaNbO_3_ NWs/PDMS composite in a volume ratio of 1:100 between Au/Cr‐coated Kapton films.^[^ ^401^ ^]^ The NaNbO_3_ NWs were randomly oriented in PDMS without aggregations and the nanocomposites were poled at RT. The PENG exhibited an output voltage of 3.2 V and a current of 72 nA under 0.23% compressive strain due to the large d_33_ coefficient of perovskite NaNbO_3_ NWs_._

Yun et al. built a flexible PENG using a nanocomposite of LiNbO_3_ NWs and PDMS in a ratio of 1:100 by volume.^[^ ^41^ ^]^ The synthesized LiNbO_3_ NWs were single‐crystalline with approximate lengths of 50 µm and had a high d_33_ value of ≈25 pC N^–1^. The PENG generated stable electric power for up to 10^5^ cycles of excessive strain conditions. This work emphasized the significance of strain geometry and filler blending ratio for obtaining higher output in nanocomposite PENGs.

Jung et al. fabricated PZT nanotubes by infiltrating PZT solution into the pores of an AAO template and annealing at 650°C to form the perovskite phase.^[^ ^44^ ^]^ The nanotubes obtained were dense and uniform with a length, diameter, and wall thickness of around 59 mm, 210 nm, and 37 nm, respectively. They were blended with PDMS in the ratio of 1:100 by volume and attached to Pt/Ti electrodes to construct a PEH. When the PEH was bent and released, it generated an output current of 54.5 nA and voltage of 1.52 V with a power density of 37 nW cm^–2^. The change in polarization in the nanocomposite under the application of external stress and poling led to a change in charge density and power generation.

**Figure 37:**
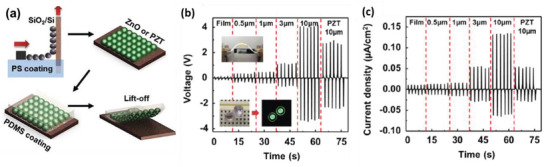
a) Schematic diagram of the fabrication process for stretchable composites embedded with hemispheres. b) Output voltage and c) current density generated by the composites embedded with ZnO hemispheres as a function of the diameter of the hemispheres from 0.5 to10 µm and PZT hemispheres of 10 µm under convex bending strain. Reproduced with permission.^[^ ^402^ ^]^ Copyright 2014, Elsevier Ltd.

Chun et al. assembled a PENG based on highly‐ordered piezoelectric hemispheres embedded in PDMS.^[^ ^402^ ^]^ The hemispheres were made via LB deposition of polystyrene spheres on planar substrates, followed by depositing ZnO or PZT film by magnetron sputtering and post‐annealing to remove the spheres (**Figure** **37**a). The composite with ZnO hemispheres of 10 µm diameter yielded an output voltage of 6 V and *j* = 0.2 µA cm^–2^ (Figure 37b,c). The composite containing PZT hemispheres of 10 µm generated a voltage of 3 V and *j* = 0.05 µA cm^–2^ which was lower than the ZnO composites (Figure 37b,c). The high strain sensitivity of the nanocomposite films was achieved by convex bending because of strong dipole alignment.

Xu et al. synthesized PMN‐PT NWs hydrothermally with lengths up to 10 µm and added them to PDMS in the ratio of 1:10 by wt%.^[^ ^403^ ^]^ To construct the PENG, the polymer nanocomposite was bonded to two Ti/Au coated polyimide electrodes, Pt leads were attached to the electrodes and finally poled. A maximum output voltage of 7.8 V and current of 2.29 µA (*j* = 4.58 µA cm^–2^) was generated from the PMN‐PT/PDMS based PENG due to the high d_33_ value of PMN‐PT NWs (371 pC N^–1[^ ^123^ ^]^) and their distinctive hierarchic structure.

Ren et al. demonstrated a flexible PENG based on bismuth ferrite (BiFeO_3_) NPs/PDMS nanocomposite using various concentrations of BiFeO_3_ (10–40 wt%).^[^ ^64^ ^]^ BiFeO_3_ with large remnant polarization (*P*_r_) is suitable for amplifying the PENG's performance as d_33_ is closely related to *P*_r_ (d_33_ ∝ *ɛP*_r_). The PENG with PDMS/BiFeO_3_ −40 wt% produced a *V*_oc_ of ≈3 V and *I*_sc_ of ≈0.25 µA under repeated hand pressing. Abinnas et al. harvested energy from finger tapping using a PENG of Bismuth titanate (BiT)/PDMS nanocomposite.^[^ ^404^ ^]^ The device produced an output voltage of 8 V after poling at 5 kV for 3 h.

**Figure 38:**
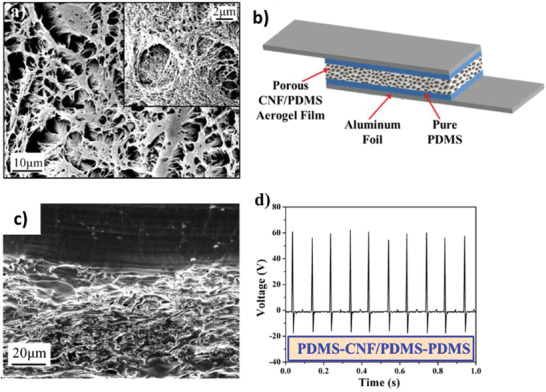
a) SEM image of the as‐fabricated CNF aerogel. b) Schematic diagram of the flexible porous CNF/PDMS aerogel film‐based PENG. c) Cross‐sectional SEM image of the PENG at high magnification. d) Output voltage generated from the PENG. Reproduced with permission.^[^ ^405^ ^]^ Copyright 2016, Elsevier Ltd.

Zheng et al. first fabricated a porous cellulose nanofibril (CNF) aerogel film (**Figure** **38**a) and then coated PDMS onto CNF via the vacuum‐assisted filling method to prepare CNF/PDMS aerogel film (Figure 38b).^[^ ^405^, ^406^ ^]^ The non‐poled PENG (Figure 38b,c) generated a reliable output with a *V*_OC_ of 60.2 V (Figure 38d), *I*_SC_ of 10.1 µA, and power density of 6.3 mW cm^–3^. It exhibited high flexibility because of the flexible nature of CNFs and PDMS. The PDMS coating on CNF generated electric dipoles under oscillating mechanical stress because of free radical formation.

Ding et al. reported a PENG based on a nanocomposite of formamidinium lead halide perovskite (FAPbBr_3_) NPs and PDMS.^[^ ^407^, ^408^ ^]^ The effective d_33_ coefficient of FAPbBr_3_ NPs is ≈25 pC N^–1^, which is comparable to BaTiO_3_ (28 pC N^–1^)^[^ ^409^ ^]^ and around six times of NaNbO_3_ NWs (4 pC N^–1^).^[^ ^410^ ^]^ The FAPbBr_3_ NPs were homogeneously mixed with PDMS at an optimized loading of 35 wt% and then spin‐coated onto an ITO‐coated PET substrate. A high output voltage of 8.5 V and *j* = 3.8 µA cm^–2^ was obtained from the PENG under 0.5 MPa vertical compression. The high performance was ascribed to the high d_33_ value of the randomly distributed FAPbBr_3_ NPs that acted as stress concentration points and generated an electric potential gradient upon mechanical compression.

Very recently, Hajra et al. developed a flexible PENG from a composite of Aurivillius‐based oxide, CaBi_4_Ti_4_O_15_ (CBTO) and PDMS.^[^ ^411^ ^]^ The PENG containing PDMS/CBTO‐8% generated a maximum output of 23 V and 85 nA with an instantaneous power density of 1.09 mW m^–2^ at 300 MΩ. It was used as a self‐powered exercise counter to track exercise repetitions of different parts of the body such as the wrist and neck.

From the literature discussed, it can be summarized that PDMS based nanocomposites have higher flexibility than PVDF based nanocomposites, making them suitable for energy harvesting from bending, rolling, twisting, etc. The fillers included materials with high piezoelectric constants such as BaTiO_3_ NPs, NWs and nanocubes, ZnSO_3_ nanocubes, NaNbO_3_ NWs, and PZT nanotubes. The content of fillers in these PDMS nanocomposites was in the range of 1–60 wt% and the synthesis techniques included solution casting, spin casting, LB deposition, and magnetron sputtering. In general, poling was done on these nanocomposites, using both conventional and corona poling methods. The highest output voltage of 126.3 V was generated from a PENG consisting of a nanocomposite of PDMS/BaTiO_3_ nanocube‐15 wt% on applying a pressure of 988.2 Pa.^[^ ^396^ ^]^

#### 5.2.2 Nanocomposites Based on Conducting and Non‐Conducting Filler Combination

This section focuses on PDMS based nanocomposites synthesized using a combination of conducting and non‐conducting fillers such as BaTiO_3_ NPs with SW/MWCNTs, BaTiO_3_ NPs with rGO, BaTiO_3_ NPs with carbon black, etc. The fabrication techniques, piezoelectric properties, and energy harvesting performance of PENGs built using these nanocomposites has been discussed thoroughly.

A PENG was fabricated by Park et al. using a nanocomposite of BaTiO_3_ NPs, SW/MWCNTs, or rGO dispersed into PDMS.^[^ ^125^ ^]^ The BaTiO_3_ NPs were spherical with a size of ≈100 nm and was the source of piezoelectric potential. The CNTs acted as a dispersant to distribute the BaTiO_3_ NPs throughout the matrix by forming conducting networks with them. The PENG containing a nanocomposite of 1 wt% MWCNTs and 12 wt% BaTiO_3_ NPs generated an output voltage of ≈3.2 V. The rGO based PENG produced a lower output voltage of ≈2.0 V when compared to the CNT based device due to differences in the degree of mixing caused by morphological differences between CNT networks and rGO structures.

**Figure 39:**
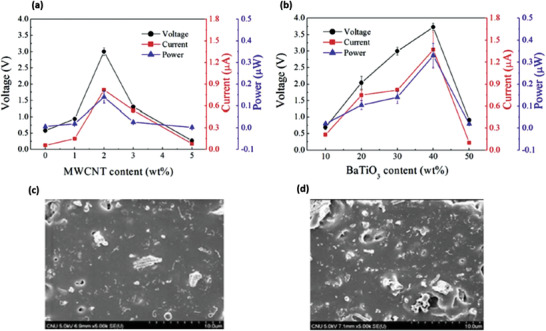
The output voltage, current, and power of PENGs generated under periodic mechanical compression of 2 kPa with a) 30 wt% BaTiO_3_ nanofibers and different MWCNT contents (0–5 wt%), b) 2.0 wt% MWCNT, and different BaTiO_3_ nanofiber contents (10–50 wt%). Cross‐sectional SEM images of PENGs with c) 2.0 wt% MWCNTs and 30 wt% BaTiO_3_ nanofibers, d) 2.0 wt% MWCNTs, and 40 wt% BaTiO_3_ nanofibers. Reproduced with permission.^[^ ^412^ ^]^ Copyright 2017, Elsevier Ltd.

Yan and Jeong made flexible nanocomposites with different contents of MWCNTs (0–5 wt%) and BaTiO_3_ nanofibers (10–50 wt%).^[^ ^412^ ^]^ Keeping the BaTiO_3_ amount constant at 30 wt% and varying MWCNTs between 0–5 wt%, the PENG with 2 wt% MWCNT produced the highest output voltage of ≈3 V, current of ≈0.82 µA, and power of ≈0.14 µW under 2 kPa periodic compression (**Figure** **39**a). This was attributed to two main reasons. First, MWCNTs with high modulus enhanced stress transfer from PDMS to BaTiO_3_ nanofibers and second, charge transfer between BaTiO_3_ NFs was improved by the presence of MWCNTs as electric bridges. The piezoelectric properties deteriorated with MWCNTs loading >2 wt% due to current leakage caused by the high conductivity of MWCNTs. When the content of BaTiO_3_ NFs was varied between 10 and 50 wt%, keeping MWCNT constant at 2 wt%, the maximum output obtained from the PENG was at 40 wt% BaTiO_3_ (3.73 V, 1.37 µA, power ≈0.33 µW) as shown in Figure 39b. This was ascribed to the symbiotic effect of BaTiO_3_ NFs and MWCNTs dispersed uniformly in the PDMS matrix. Again, at BaTiO_3_ loadings >40 wt%, the outputs dropped due to current leakage and non‐uniform distribution of BaTiO_3_ in PDMS. The cross‐sectional SEM images of PENGs showing the best performance in both cases are shown in Figure 39c,d.

Luo et al. developed a flexible PENG using nanocomposites of 30 wt% BaTiO_3_ and 0–4.8 wt% CB added to PDMS.^[^ ^413^ ^]^ In the nanocomposites, CB NPs of diameter ≈30 nm was used as the conductive phase and the d_33_ value was 15.3 pC N^–1^. The maximum output generated from the PENG was 7.43 V when CB loading was 3.2 wt% in the nanocomposite. As the amount of CB increased in PDMS matrix up to a certain threshold, the piezoelectricity of the nanocomposites increased due to the synergistic effect of PDMS, BaTiO_3_, and CB.

Sun et al. developed a flexible PENG consisting of ZnO NPs/MWCNTs/PDMS nanocomposites.^[^ ^414^ ^]^ ZnO NPs and CNTs were added to PDMS in a mass ratio of ZnO/CNT/PDMS = 12/1/87. The diameter of ZnO NPs was 30 nm, diameter, and length of CNTs were 8–15 nm and 50 µm, respectively. A high output voltage of 7.5 V and a power density of 18.75 µW per cycle was displayed by the PENG during the hammer knocking process. The addition of MWCNTs significantly enhanced the PENG's voltage output from 0.8 to 7.5 V due to increased conductivity of the nanocomposites and prevention of internal energy dissipation.

Park et al. added 12 wt% PZT particles and 1 wt% MWCNTs into PDMS and showed that PZT particles of ≈1 µm diameter were well entangled with MWCNTs having a length of ≈10 µm and diameter of ≈15 nm.^[^ ^415^ ^]^ The PZT particles showed excellent crystallinity and a tetragonal phase exhibiting outstanding piezoelectric properties. MWCNTs were added to provide mechanical reinforcement to the PDMS matrix and a conduction path to enhance the output performance of the PENG. A large‐area PENG of 30 cm x 30 cm was fabricated that harnessed human movement to produce an electric output of ≈100 V and ≈10 µA after poling.

**Figure 40:**
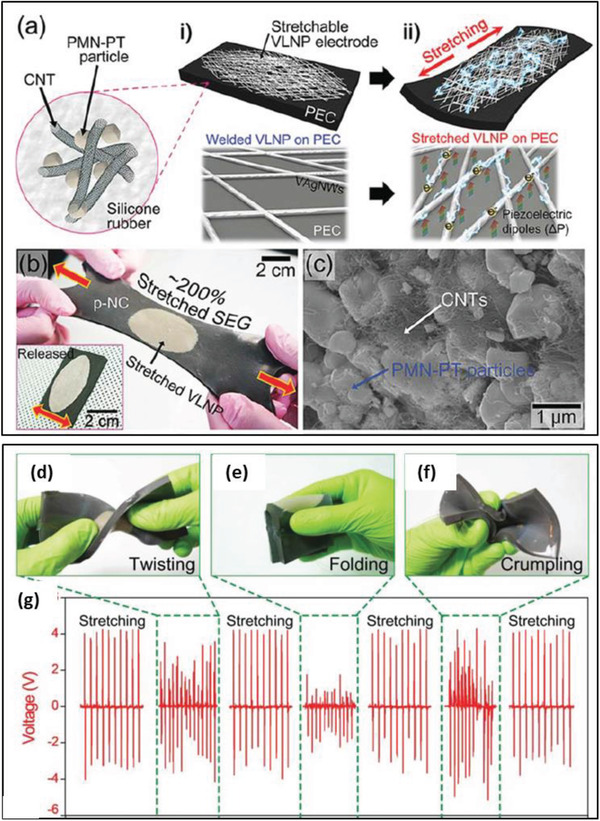
a) Schematic diagram of the hyper‐stretchable PENG based on PMN‐PT/MWCNT composite and Ag NWs stretchable electrodes. b) The PENG device stretched by human hands. Inset showing PENG is released again without damage. c) SEM image showing well‐distributed PMN‐PT particles and MWCNTs in the silicone rubber matrix. d–f) Photographs of the PENG when subjected to various deformations, such as twisting, folding, and crumpling. g) These motions are converted to a corresponding voltage. Reproduced with permission.^[^ ^416^ ^]^ Copyright 2015, Wiley‐VCH.

Jeong et al. reported a hyper‐stretchable elastic PENG (**Figure** **40**) comprising of well‐distributed PMN‐PT and MWCNTs in silicone rubber matrix (Figure 40c).^[^ ^416^ ^]^ Eco flex silicone rubber was selected as the matrix because it is a hyper stretchable elastomer with elongations up to ≈900%, which is exceedingly superior to PDMS. Long Ag NWs percolation electrodes were used with an average length of ≈150 µm, synthesized by an innovative multistep growth process (Figure 40a).^[^ ^417^ ^]^ After curing the elastomeric polymer nanocomposite, the Ag‐NWs were transferred onto it by the solution filtration method. The PENG exhibited excellent electrical output of ≈4 V and ≈500 nA with a stretchability of 200% (Figure 40b). An outstanding performance was obtained because the piezo potential was induced throughout the composite due to the high elongation ability of the elastomeric matrix and the presence of Ag‐NWs stretchable electrodes. The PENG also generated electricity when subjected to various deformations like twisting, folding, and crumpling (Figure 40d–g), illustrating its extreme flexibility.

Jeong et al. dispersed (K, Na) NbO_3_‐LiNbO_3_ (KNLN) particles, and Cu nanorods in PDMS to prepare a hybrid nanocomposite.^[^ ^418^ ^]^ The optimum ratio of KNLN to Cu NRs was 2:1 in 10 g of PDMS. The flexible PENG produced high output of ≈12 V and ≈1.2 µA from bending/hand tapping due to the high intrinsic piezoelectric potential of KNLN particles.

Alam and Mandal fabricated a flexible PENG based on cellulose microfibers and PDMS, with MWCNTs as conducting fillers.^[^ ^419^ ^]^ Superior piezoelectric performance was achieved without the chemical treatment of cellulose and poling. The PENG, on repeated human hand punching, displayed a *V*_OC_ of ≈30 V and an *I*_SC_ of ≈500 nA with a power density ≈of 9.0 µW cm^–3^. The breaking of chains (Si—O—Si groups) in PDMS upon applying mechanical pressure caused the opening of more OH groups that formed hydrogen bonding with oxygen atoms of cellulose resulting in increased piezoelectric dipole density.

Thus, it can be concluded that PDMS nanocomposites based on a combination of conducting and non‐conducting fillers have higher piezoelectric output compared to PVDF based nanocomposites synthesized using the same filler combination. Materials such as BaTiO_3_ NPs and carbon black, PZT, and MWCNTs, cellulose microfiber and MWCNTs, ZnO NPs, and MWCNTs, etc., were used as fillers in the range of 0.5–50 wt%. The methods for synthesizing these nanocomposites consisted of ultrasonic mixing with spin casting, ball milling, blending, and curing, followed by poling in general. The highest output of 100 V was obtained from a large area PENG of 30 cm x 30 cm containing a nanocomposite of 12 wt% PZT, 1 wt% MWCNTs, and PDMS when periodic stress was applied by a linear motor.^[^ ^415^ ^]^ Table 3 summarizes the recent literature on piezoelectric polymer nanocomposites for energy harvesting applications.

## 6 Piezoelectric Polyvinylidene Fluoride Films Doped with Inorganic Salts

In recent years, researchers have been investigating the effect of inorganic salts such as iron nitrate [Fe(NO_3_)_3_],^[^ ^420^ ^]^ zinc nitrate [Zn(NO_3_)_2_],^[^ ^421^ ^]^ magnesium chloride (MgCl_2_),^[^ ^422^ ^]^ nickel chloride (NiCl_2_),^[^ ^423^ ^]^ lithium chloride (LiCl),^[^ ^424^ ^]^ iron chloride (FeCl_2_)^[^ ^425^ ^]^ and bismuth chloride (BiCl_3_),^[^ ^426^ ^]^ on piezoelectric properties of PVDF. The addition of salts promotes *β*‐phase formation in PVDF and increases its conductivity which enhances its piezoelectric/energy harvesting performance. This section summarizes the recent work conducted in this field.

**Figure 41:**
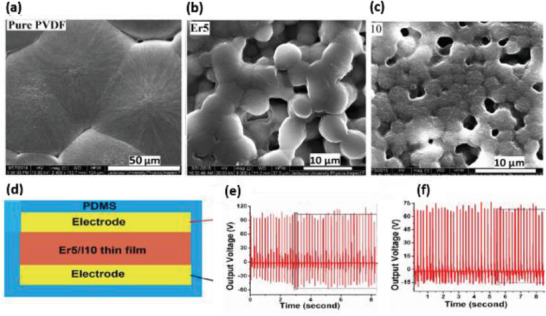
FESEM images of a) pure PVDF, b) ErCl_3_.6H_2_O/PVDF films with 5 wt% Er, c) Fe(NO_3_)_3_·9H_2_O/PVDF films with 10 wt% Fe. d) Schematic of the PENG device. Open circuit voltage (*V*_oc_) generated by the PENGs containing e) 5 wt% Er^3+^/PVDF and f) 10 wt% Fe^3+^/PVDF composites. Reproduced with permission.^[^ ^420^ ^]^ Copyright 2017, American Chemical Society.

Hoque et al. examined the effect of erbium chloride hexahydrate (ErCl_3_.6H_2_O) and iron nitrate nonahydrate [Fe(NO_3_)_3_.9H_2_O] salt molecules on PVDF *β*‐phase nucleation.^[^ ^420^ ^]^ The maximum *β*‐phase obtained was ≈83.65% and ≈82.15% in PVDF/Er‐5 wt% and PVDF/Fe‐10 wt% nanocomposites. Spherulites of ≈5 µm diameters are present on the surfaces of these composite films, validating *β*‐crystal nucleation (**Figure** **41**b,c). Whereas, pure PVDF films show spherulites with ≈40 µm diameters, specifying the existence of nonpolar *α*‐crystals (Figure 41a). This was ascribed to the efficient electrostatic interactions between H_2_O molecules present in salts and the —CF_2_ dipoles of PVDF via hydrogen bonding.^[^ ^428^ ^]^ The d_33_ values were −124 and −72.4 pC N^–1^ for the same compositions, showing that Er^3+^ was more effective in nucleating *β* phase than Fe^3+^ due to its bigger size and higher ability of covalent bond formation. A schematic of the PENG device is shown in Figure 41d. Under periodic finger impartation, the Er^3+^ and Fe^3+^ based PENGs displayed a large *V*_oc_ of ≈115 V (Figure 41e), *I*_sc_ of ≈32 µA, the power density of ≈160 mW cm^–3^ and *V*_oc_ of ≈75 V (Figure 41f), *I*_sc_ of ≈17 µA, the power density of ≈55.34 mW cm^–3^, respectively. In another work, Fortunato et al. showed *β* phase increase by incorporating 0.2 wt% zinc nitrate hexahydrate [Zn(NO_3_)_2_.6H_2_O], magnesium nitrate hexahydrate [Mg(NO_3_)_2_.6H_2_O], magnesium chloride hexahydrate [MgCl_2_.6H_2_O], and aluminum chloride hexahydrate [AlCl_3_.6H_2_O] into PVDF.^[^ ^421^ ^]^ The best output performance (F(*β*) = 82%, d_33_ ≈ 13.49 pC N^–1^) was attained in Mg(NO_3_)_2_.6H_2_O/PVDF nanocomposites and the worst performance was obtained in iron chloride hexahydrate [FeCl_3_.6H_2_O]/PVDF nanocomposites. Similar studies were done by Thakur et al. using 1–30 wt% of cerium/yttrium nitrate hexahydrate salts [Ce(NO_3_)_3_.6H_2_O and Y(NO_3_)_3_.6H_2_O] and F(*β*) value of 82% was reported.^[^ ^427^ ^]^

Jana et al. fabricated a high‐performance PENG by using PVDF films filled with various wt% of MgCl_2_.6H_2_O.^[^ ^422^ ^]^ The PENG delivered a *V*_oc_ of up to 4 V under a pressure of ≈4.45 kPa imparted by a human finger. Dhakras et al. showed a *β*‐phase increase by about 30% by adding NiCl_2_.6H_2_O to electrospun PVDF nanofibers.^[^ ^423^ ^]^ The Vp‐p produced from PVDF/NiCl_2_ film was nearly 0.762 V, about three times higher than that of PVDF. In another study, Yu et al. doped twenty‐six salts such as AlCl_3_, FeCl_2_⋅4H_2_O, SnCl_4_⋅5H_2_O, etc., into PVDF nanofibers and studied their piezoelectric properties.^[^ ^425^ ^]^ The results indicated that different salts had differing piezoelectric enhancement effects and different optimum doping ratios. The PENG fabricated with optimized PVDF nanofibers displayed a piezo voltage seven times greater than that of a device made from undoped nanofibers. Recently, Mokhtari et al. developed a flexible PENG based on electrospun PVDF/LiCl nanofibers.^[^ ^424^ ^]^ The authors showed an increase in output voltage from 1.3 to 5 V due to the addition of LiCl into the spinning solution in comparison to pure PVDF. The PENG generated an output of 3 V and 0.5 µA with a power density of 0.3 µW cm^–2^ and was utilized as a sensor to measure temperature changes between 30 and 90 °C. Likewise, Chen et al. used polar additive BiCl_3_ to increase *β*‐phase concentration in PVDF nanofibers.^[^ ^426^ ^]^ The PENG based on an optimized ratio of 2 wt% BiCl_3_ delivered an output voltage of 1.1 V, which was 4.76 times higher than that of pure PVDF based PENG. The peak current and power densities were 2 µA and 0.2 µW cm^–2^, respectively. The mechanism for improved piezo response in these PVDF doped chlorides is the same as explained above for Fe(NO_3_)_3_.9H_2_O and ErCl_3_.6H_2_O.

## 7 Piezoelectric Films of Other Materials

In this section, we will briefly discuss piezoelectric films fabricated by various fabrication techniques for scalable and flexible energy harvesting applications. These fabrication techniques enable the deposition of advanced piezoelectric films with high crystallinity and controlled morphology for integration into energy harvesters to obtain enhanced output performance.

**Figure 42:**
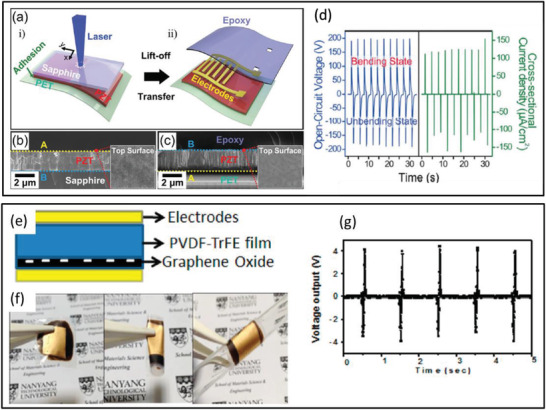
a) Schematic diagram of the fabrication process for PZT thin film‐based PENG via LLO method. Cross‐sectional SEM images of PZT thin films on b) Al_2_O_3_ and c) PET substrate. d) *V*_oc_ and cross‐sectional current density measured from the PENG. a‐d) Reproduced with permission.^[^ ^428^ ^]^ Copyright 2014, Wiley‐VCH. e) Schematic of the bilayer films PEH. f) Photograph showing that the PEH is freestanding, flexible, bendable, and rollable. g) The voltage output from the PEH. e‐g) Reproduced with permission.^[^ ^429^ ^]^ Copyright 2015, American Chemical Society.

Park et al. fabricated a large‐sized PENG using PZT film on flexible substrates via a laser lift‐off (LLO) process.^[^ ^428^ ^]^ Deposition of PZT film was initially done on bulk Al_2_O_3_ substrate by conventional sol–gel method. It was then transferred onto the PET substrate using laser irradiation without any mechanical damage (**Figure** **42**a). Subsequently, interdigitated gold electrodes were deposited onto PZT films. Figure 42b,c are cross‐sectional SEM images of PZT thin films deposited on the original Al_2_O_3_ substrate and receiver PET substrate before and after the LLO process. The PENG showed remarkable output performance of 200 V and *j* = 150 µAcm^–2^ from slight mechanical deformations (Figure 42d). It operated 105 LED arrays without any external electric source.

Bhavanasi et al. fabricated a PEH using poled P(VDF‐TrFE) and charged GO films (Figure 42e), exhibiting high Young's modulus and dielectric constant.^[^ ^429^ ^]^ The bilayer PEH device was freestanding, flexible, bendable, and rollable as illustrated in Figure 42f. Superior energy harvesting performance was exhibited by the PEH (voltage ≈ 4 V and power density ≈ 4.41 µW cm^–2^), compared to P(VDF‐TrFE) films alone (voltage ≈ 1.9 V and power density ≈ 1.77 µW cm^−2^) (Figure 42g). The excellent performance was attributed to the electrostatic contribution from GO, residual stress, and the presence of space charges at the P(VDF‐TrFE)/GO interface originating from positively charged P(VDF‐TrFE) and negatively charged functional groups in GO.

Park et al. deposited BaTiO_3_ thin films on a Pt/Ti/SiO_2_/Si bulk substrate by magnetron sputtering and then transferred them to a plastic substrate using conventional microfabrication and lithographic techniques.^[^ ^430^ ^]^ Without poling, the d_33_ value of the BaTiO_3_ thin film was 40 pC N^–1^, which increased to 105 pC N^–1^ after poling under an electric field of 100 kV cm^–1^ at 140 °C. The PENG transformed energy from bending into electricity, generating an output voltage of up to ≈1.0 V and *j* = 0.19 µA cm^–2^. Later, Lim et al. constructed a PEH based on a BaTiO_3_‐resin film via an inkjet‐printing process without high‐temperature annealing and complex transfer processes.^[^ ^431^ ^]^ The PENG successfully harvested an output voltage of ≈7 V and a current of 2.5 µA from cyclic mechanical deformations.

Lee et al. enhanced the power output of the PENG by introducing a conductive p‐type polymer, poly(3‐hexylthiophene) (P3HT) onto ZnO thin film.^[^ ^432^ ^]^ Also, phenyl‐C_61_‐butyric acid methyl ester (PCBM) was added to P3HT to improve carrier transport. At 0.068% strain, the ZnO/P3HT: PCBM‐assembled PENG exhibited an 18‐fold increase in output voltage and three times increase in current when compared to a PENG with ZnO only.

The literature discussed in this section highlights that the fabrication methods for depositing piezoelectric films play a crucial role in the piezoelectric performance of these materials. The fabrication methods range from LLO and sol–gel processing to magnetron sputtering, microfabrication, and lithographic techniques. The PZT film‐based PENG assembled on flexible substrates showed the most remarkable output performance of 200 V and *j* = 150 µAcm^–2^ from slight mechanical deformations.^[^ ^428^ ^]^

## 8 Applications

The power generated by an energy harvester has random variation and dual‐polarity voltage peaks due to the random nature of the input source. To make this power usable, it is converted to DC power with single polarity and controlled voltage. In the case of PENGs, this is commonly done by using a bridge rectifier that is connected to a capacitor, which gets charged by utilizing the rectified output.^[^ ^23^ ^]^ The capacitor is connected to an external storage device to store the power for extended periods. Such circuits have been commonly used to power LEDs and LCDs after enough charge has been stored.^[^ ^120^, ^293^, ^433^ ^]^ PENGs have also been used in self‐powered systems for water velocity and pH detection sensors,^[^ ^434^, ^435^ ^]^ deformation detection,^[^ ^436^, ^437^ ^]^ and human motion monitoring.^[^ ^438^ ^]^ This section reviews these applications in detail.

**Figure 43:**
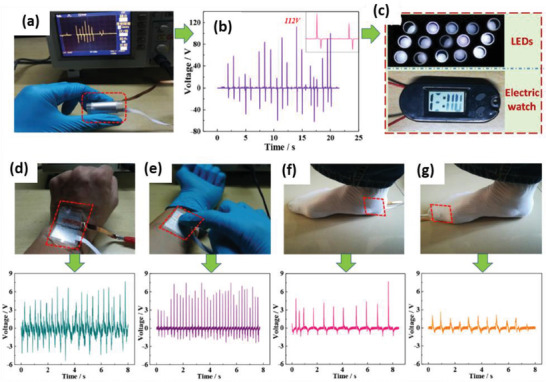
a) Optical image of the PENG under finger pressing. b) Output voltage generated from the PENG by finger pressing‐releasing process. c) A commercial electric watch and 15 LEDs driven by the PENG. The optical images and output voltages generated by human motions of d) wrist flexing, e) finger taping, and foot stepping by f) heel and g) toe. Reproduced with permission.^[^ ^433^ ^]^ Copyright 2018, Elsevier Ltd.

Shi et al. used electrospun fiber mats comprising of 15 wt% BaTiO_3_ NPs, 0.15 wt% graphene nanosheets, and PVDF to fabricate a flexible PENG.^[^ ^433^ ^]^ A peak voltage of 112 V was generated by the PENG in the course of the finger pressing‐releasing process at fast strain rates, which drove an electric watch and lit up 15 LEDs (**Figure** **43**a–c). It also harvested energy from human movements such as finger tapping, wrist flexing, and foot stepping. Under wrist flexing and finger tapping conditions, the output voltage reached 7.7 and 7.5 V, respectively (Figure 43d,e). When the PENG was located under the foot heel and toe, it produced a maximum voltage of 7.8 and 2.8 V, respectively (Figure 43f,g). The heel exerted greater pressure when compared to the toe, hence it resulted in higher output voltage.

**Figure 44:**
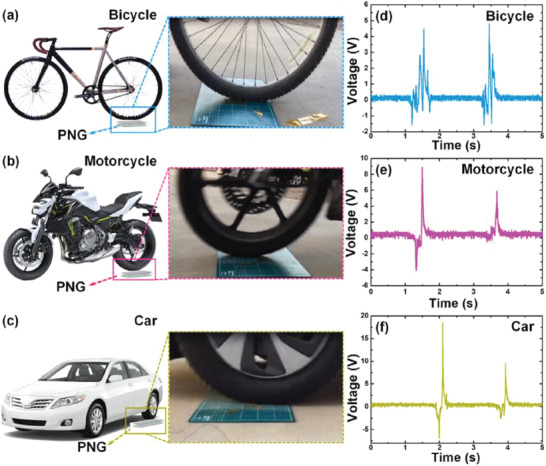
a–c) Schematic and photographic images of different vehicles such as bicycle, motorcycle, and car passing on the Ag/BaTiO_3_‐PENG device. d–f) The corresponding output voltage from the PENG. Reproduced with permission.^[^ ^439^ ^]^ Copyright 2018, Elsevier Ltd.

Dudem et al. configured a PENG using a composite of BaTiO_3_ micro stones (MSs) and Ag NWs in the PVDF matrix.^[^ ^439^ ^]^ The composite containing 7.5 wt% BaTiO_3_ MSs and 7.5 vol% Ag NWs in 20 mL of PVDF resulted in high *V*_OC_ of ≈52 V and *I*_SC_ of ≈3.2 µA from the PENG under a pushing force of 15 N. The PENG effectively harvested energy from automotive vehicle motion such as bicycle, motorcycle, and cars as shown in **Figure** **44**a–f. The impact force applied on the Ag/BaTiO_3_‐PENG by the motion of vehicles was efficiently converted into electricity.

**Figure 45:**
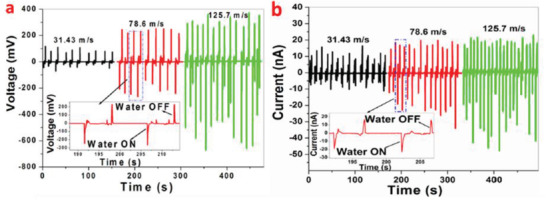
a,b) *V*_oc_ and *I*_sc_ generated by PENG at water velocities of 31.43, 78.6, and 125.7 ms^–1^. The insets of (a,b) show output generated under water ON and OFF conditions. Reproduced with permission.^[^ ^434^ ^]^ Copyright 2015, American Chemical Society.

Alluri et al. successfully operated a PENG as a self‐powered sensor to determine water velocities in a pipe at high temperatures and pressures.^[^ ^434^ ^]^ The PENG was based on a composite of BaTi_(1−_ *_x_*_)_Zr*_x_*O_3_ (BTZO) nanocubes and PVDF in a weight ratio of 1:0.25. BTZO formation took place by substitution of Ti^4+^ atom (0.605 Å) in BaTiO_3_ with Zr^4+^ atom (0.72 Å). BTZO has a higher d_33_ of 174–236 pC N^–1^, compared to 100 pC N^–1^ for BaTiO_3_. The PENG was driven by a faucet with water ON and OFF conditions over periodic time intervals; **Figure** **45**a,b shows the electrical output at different water velocities. The output voltage and current were 26 mV and 8 nA at a water velocity of 31.43 ms^–1^ during the ON condition and were 80 mV and 10 nA during the OFF condition. When water speed increased from 31.43 to 78.6 to 125.7 ms^–1^, the outputs pertaining to them increased as well when water was ON. When water was OFF, the induced charges moved back to their initial locations. The mechanism involved is similar to the pushing and releasing force exerted on the PENG.

**Figure 46:**
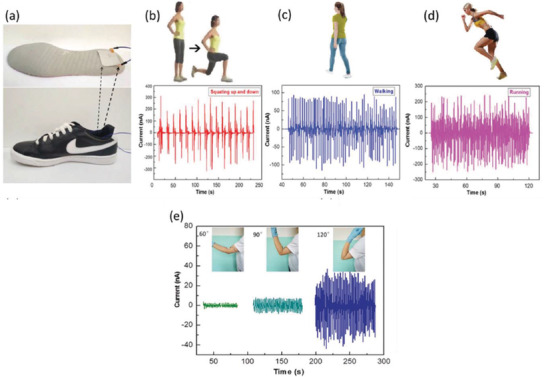
a) Photographs of the pressure sensor attached to a shoe insole. b–d) Schematic diagrams and the output currents produced by squatting up and down, walking, and running. e) Output currents with elbow flexion and extension for 60°, 90°, and 120°. Reproduced with permission.^[^ ^438^ ^]^ Copyright 2018, The Royal Society of Chemistry.

Guo et al. developed a wireless device comprising of a pressure sensor built from electrospun fibers of PVDF/BaTiO_3_ NWs‐3 wt%.^[^ ^438^ ^]^ When the sensor was attached to the shoe insole, the current was generated upon squatting up and down, walking, and running motions (**Figure** **46**a–d). The output current generated from walking was about 97 nA, which was less than 241 nA from running and 331 nA from squatting up and down based on impact intensity (Figure 46b–d). An increased output current was observed with an increase in the angle of elbow flexion, producing a maximum current of 36 nA at 120° (Figure 46e).

**Figure 47:**
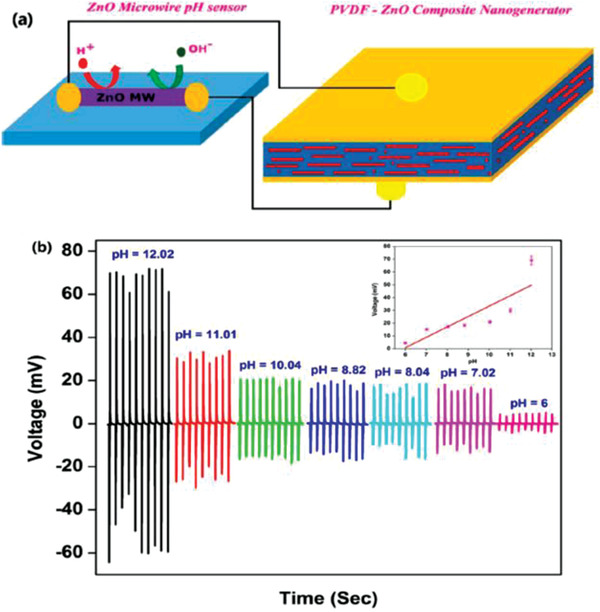
a) Demonstration of the self‐powered device consisting of a ZnO microwire pH sensor and a composite nanogenerator. b) Electrical output as a function of pH across the pH sensor. The inset shows the voltage as a function of different pH values. Reproduced with permission.^[^ ^435^ ^]^ Copyright 2014, American Chemical Society.

Saravanakumar et al. developed a PENG using a nanocomposite of ZnO NWs/PVDF and it was used to drive a pH sensor without external power^[^ ^435^ ^]^ (**Figure** **47**a). Figure 47b shows a plot of the output voltage with respect to time for different pH levels. The presence of negatively charged species (OH—) raised the resistance of the microwire at higher pH (base) values. As a result, a drop in potential was observed across the microwire for a 12.02 pH value. At lower pH values (acidic), H+ ions were abundant; hence it lowered the resistance across the microwire. The pH changed from basic to acidic (12.02 to 6) with a corresponding reduction in voltage output.

**Figure 48:**
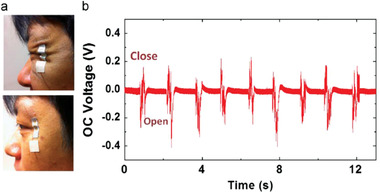
a) PENG affixed to the skin near a human eye for detecting wrinkling of the face. b) Output signal recorded while blinking of an eye. Reproduced with permission.^[^ ^23^ ^]^ Copyright 2014, Elsevier Ltd.

A highly flexible PENG was built on an ultra‐thin aluminum foil to function as a deformation sensor for detecting wrinkling of the human face and tracking the motion of eyeballs.^[^ ^23^, ^436^, ^437^ ^]^ The PENG comprising of ZnO NWs (size 5 mm x 13 mm) was affixed to the human skin close to an eye (**Figure** **48**a). When the eye blinked, the dynamic wrinkles caused the PENG to deform due to its super flexibility and conformability. The output voltage and current were approximately 0.2 V and 2 nA during eye blinking motion (Figure 48b).

Lee et al. demonstrated another application of the PENG as an autonomous sensor to detect mercury (Hg^2+^) concentration in water via the emitting intensity of a LED.^[^ ^440^ ^]^ The LED‐lit up based on the concentration of Hg^2+^ in water droplets and therefore was used for environmental monitoring. In another study, Hu et al. integrated a PENG in a vehicle tire for pressure and speed monitoring.^[^ ^441^ ^]^ An electric signal was generated when the tire touched or de‐touched the road surface while moving. A lower pressure resulted in a flatter tire that exerted higher deformation on the PENG and resulted in higher voltage. Similarly, Lin et al. harvested energy from the motion of a vehicle using a ZnO NW based PENG and used it as an autonomous sensor for detecting the speed of automobiles and their weight.^[^ ^442^ ^]^ Due to the flexibility of PVDF/ZnO NW based PENG, it has also been used to measure ambient wind velocity and detect gas/liquid flow.^[^ ^443^ ^]^ These applications demonstrate that polymer nanocomposites are the most commonly used as they offer versatility compared to piezoelectric polymers or nanostructured materials.

## 9 Summary and Future Perspective

**Figure 49:**
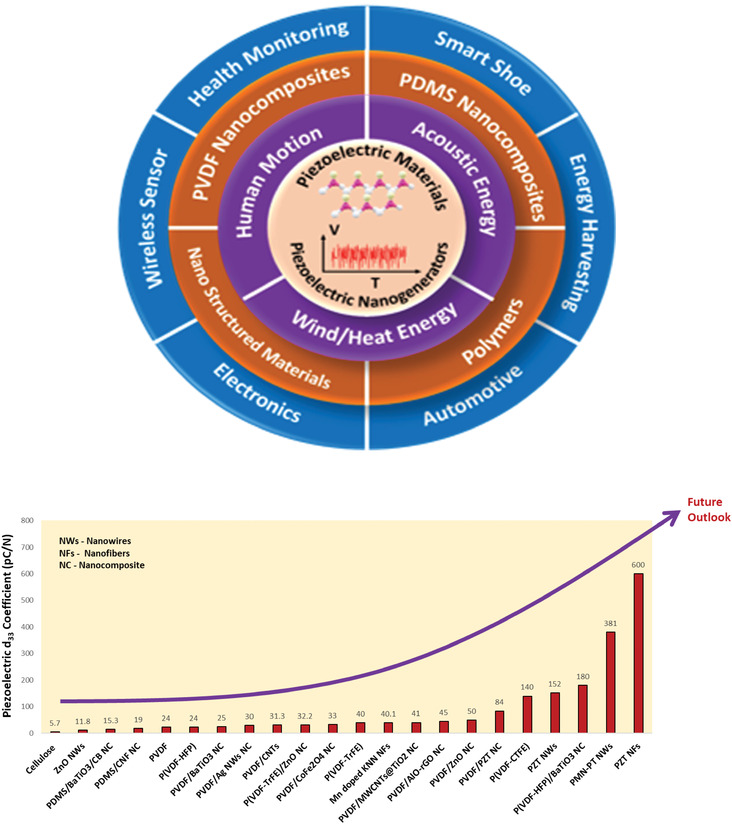
a) Schematic showing the various energy sources from which piezoelectric power is generated, the different piezoelectric materials, PENGs and their fabrication techniques and the various applications in the center. b) The plot of piezoelectric coefficients (d_33_) of various materials. The arrow points toward future outlook suggesting that a material with higher d_33_ than 600 pC N^–1^ needs to be developed to meet the piezoelectric requirements of the future.

A detailed review of the recent progress in piezoelectric materials for various applications in energy harvesting and self‐powered sensors has been presented in this article. Piezoelectric properties of various types of materials, ranging from nanostructured materials to polymers, polymer nanocomposites, and piezoelectric films have been discussed, in close connection to progress in fabrication techniques, morphology, energy harvesting performance, and underpinning fundamental mechanisms. The schematic in **Figure** **49**a briefly summarizes the various energy sources from which piezoelectric power is generated, the different piezoelectric materials, PENGs and their fabrication techniques. The center of the image shows the different applications such as wireless sensor, electronics, health monitoring, smart shoes, automotive, and touch sensors. The majority of the PENG devices reported in the literature are focused on scavenging energy from vibration and strain because of their abundance in the ambient environment around us. Technological advances are being made to improve the efficiency of light and temperature‐based piezoelectric energy harvesting in future.

The power output of a PENG is dependent on multiple internal and external factors that cause high variation in power output. Even today, low, unstable output performance, and narrow bandwidth of the PENGs are the most grueling issues, which need to be resolved before they can be commercialized. Effective power management circuits should be designed to work at a wide frequency range with the capability to process input signals of various amplitudes. A key area of advancement would be developing piezoelectric materials that have very high piezoelectric d_33_ coefficients, that is, greater than 600 pC N^–1^ to meet the piezoelectric requirements of the future. (Figure 49b). Also, the fabrication process of nanogenerators should be well suited to the high‐volume manufacturing processes used in industries for their integration into self‐powered devices. Other factors that should be considered include effective system design, packaging techniques, and testing methods, which highlights the required interdisciplinary across traditional disciplines.

Energy harvesting offers remarkable benefits to the development of the IoTs and for enabling new applications in almost every field such as smart homes, smart cities, smart factories, health and environmental monitoring, and intelligent transportation. It is a crucial component for building autonomous and mobile technologies that can operate for long periods without needing battery charges. Hence, it drives cost savings either by postponing battery replacement or by not using batteries at all. The demand for energy harvesting is driven by the increase in consumer electronics products such as computer peripherals, electronic bracelets, watches, and surveillance cameras. The emergence of wearable sensors is also creating significant growth opportunities for energy harvesting. It is expected that technological advances in materials, device integration, and manufacturing processes in the piezoelectric energy sector will play an important role in resolving the global energy crisis by developing sensors and self‐sufficient batteries. Energy harvesting in the automotive sector is continuously increasing, especially in the electric vehicle segment. This shows that energy harvesting offers endless future possibilities to satisfy growing energy demands.

## Conflict of Interest

The authors declare no conflict of interest.

## References

[1] T. W. Brown, T. Bischof‐Niemz, K. Blok, C. Breyer, H. Lund, B. V. Mathiesen, Renewable Sustainable Energy Rev. 2018, 92, 834.

[2] J. A. Paradiso, T. Starner, IEEE Pervasive Comput. 2005, 4, 18.

[3] C. Wei, X. Jing, Renewable Sustainable Energy Rev. 2017, 74, 1.

[4] A. Dewan, S. U. Ay, M. N. Karim, H. Beyenal, J. Power Sources 2014, 245, 129.

[5] K. V. Selvan, M. S. M. Ali, Renewable Sustainable Energy Rev. 2016, 54, 1035.

[6] P. K. D. Pramanik, N. Sinhababu, B. Mukherjee, S. Padmanaban, A. Maity, B. K. Upadhyaya, J. B. Holm‐Nielsen, P. Choudhury, IEEE Access 2019, 7, 182113.

[7] M. Tawalbeh, A. Eardley, L. Tawalbeh, Procedia Comput. Sci. 2016, 94, 183.

[8] C. Covaci, A. Gontean, Sensors 2020, 20, 3512. 10.3390/s20123512 PMC7349337 32575888

[9] C. R. Bowen, H. A. Kim, P. M. Weaver, S. Dunn, Energy Environ. Sci. 2014, 7, 25.

[10] H. Li, C. Tian, Z. D. Deng, Appl. Phys. Rev. 2014, 1, 041301.

[11] J. F. Tressler, S. Alkoy, R. E. Newnham, J. Electroceram. 1998, 2, 257.

[12] S. B. Lang, S. Muensit, Appl. Phys. A 2006, 85, 125.

[13] M. D. Maeder, D. Damjanovic, N. Setter, J. Electroceram. 2004, 13, 385.

[14] Y.‐Y. Chiu, W.‐Y. Lin, H.‐Y. Wang, S.‐B. Huang, M.‐H. Wu, Sens. Actuators, A 2013, 189, 328.

[15] L. T. Beringer, X. Xu, W. Shih, W.‐H. Shih, R. Habas, C. L. Schauer, Sens. Actuators, A 2015, 222, 293.

[16] S. Khan, W. Dang, L. Lorenzelli, R. Dahiya, IEEE Trans. Semicond. Manuf. 2015, 28, 486.

[17] K. Song, N. Ma, Y. K. Mishra, R. Adelung, Y. Yang, Adv. Electron. Mater. 2019, 5, 1800413.

[18] R. Zhao, N. Ma, J. Qi, Y. K. Mishra, R. Adelung, Y. Yang, Adv. Electron. Mater. 2019, 5, 1800791.

[19] Z. L. Wang, R. Yang, J. Zhou, Y. Qin, C. Xu, Y. Hu, S. Xu, Mater. Sci. Eng., R 2010, 70, 320.

[20] D. L. Polla, L. F. Francis, Annu. Rev. Mater. Sci. 1998, 28, 563.

[21] H. J. M. T. S. Adriaens, W. L. De Koning, R. Banning, IEEE/ASME Trans. Mechatronics 2000, 5, 331.

[22] D. Hu, M. Yao, Y. Fan, C. Ma, M. Fan, M. Liu, Nano Energy 2019, 55, 288.

[23] Y. Hu, Z. L. Wang, Nano Energy 2015, 14, 3.

[24] Z. Ahmad, A. Prasad, K. Prasad, Phys. B 2009, 404, 3637.

[25] A. Tamang, S. K. Ghosh, S. Garain, M. Alam, J. Haeberle, K. Henkel, D. Schmeisser, D. Mandal, ACS Appl. Mater. Interfaces 2015, 7, 16143. 26189605 10.1021/acsami.5b04161

[26] D. Vatansever, R. L. Hadimani, T. Shah, E. Siores, Smart Mater. Struct. 2011, 20, 055019.

[27] C. Liu, B. Hua, S. You, C. Bu, X. Yu, Z. Yu, N. Cheng, B. Cai, H. Liu, S. Li, L. Zhang, S. Wang, K. Liu, N. Zhang, W. Liu, S. Guo, X.‐Z. Zhao, Appl. Phys. Lett. 2015, 106, 163901.

[28] J. X. Lei, Y. Qiu, D. C. Yang, H. Q. Zhang, B. Yin, J. Y. Ji, Y. Zhao, L. Z. Hu, J. Renewable Sustainable Energy 2015, 7, 033115.

[29] M. Mintken, M. Schweichel, S. Schröder, S. Kaps, J. Carstensen, Y. K. Mishra, T. Strunskus, F. Faupel, R. Adelung, Nano Energy 2019, 56, 420.

[30] S. K. Karan, S. Maiti, Ju H. Lee, Y. K. Mishra, B. B. Khatua, J. K. Kim, Advanced Functional Materials 2020, 30, 2004446.

[31] K. S. Ramadan, D. Sameoto, S. Evoy, Smart Mater. Struct. 2014, 23, 033001.

[32] S. Priya, H.‐C. Song, Y. Zhou, R. Varghese, A. Chopra, S.‐G. Kim, I. Kanno, L. Wu, D. S. Ha, J. Ryu, R. G. Polcawich, Energy Harvesting Syst. 2019, 4, 3.

[33] K. A. Cook‐Chennault, N. Thambi, A. M. Sastry, Smart Mater. Struct. 2008, 17, 043001.

[34] P. D. Mitcheson, E. M. Yeatman, G. K. Rao, A. S. Holmes, T. C. Green, Proc. IEEE 2008, 96, 1457.

[35] Z. L. Wang, G. Zhu, Y. Yang, S. Wang, C. Pan, Mater. Today 2012, 15, 532.

[36] R. A. Surmenev, T. Orlova, R. V. Chernozem, A. A. Ivanova, A. Bartasyte, S. Mathur, M. A. Surmeneva, Nano Energy 2019, 62, 475.

[37] I. I. Slowing, J. L. Vivero‐Escoto, C.‐W. Wu, V. S.‐Y. Lin, Adv. Drug Delivery Rev. 2008, 60, 1278. 10.1016/j.addr.2008.03.012 18514969

[38] A. S. Motamedi, H. Mirzadeh, F. Hajiesmaeilbaigi, S. Bagheri‐Khoulenjani, M. A. Shokrgozar, J. Biomed. Mater. Res., Part A 2017, 105, 1984. 10.1002/jbm.a.36050 28256789

[39] G.‐T. Hwang, Y. Kim, J.‐H. Lee, S. Oh, C. K. Jeong, D. Y. Park, J. Ryu, H. Kwon, S.‐G. Lee, B. Joung, D. Kim, K. J. Lee, Energy Environ. Sci. 2015, 8, 2677.

[40] K. K. Shung, J. M. Cannata, Q. F. Zhou, J. Electroceram. 2007, 19, 141.

[41] B. Yun, Y. Park, M. Lee, N. Lee, W. Jo, S. Lee, J. Jung, Nanoscale Res. Lett. 2014, 9, 4. 24386884 10.1186/1556-276X-9-4 PMC3895797

[42] G.‐T. Hwang, H. Park, J.‐H. Lee, S. Oh, K.‐I. Park, M. Byun, H. Park, G. Ahn, C. K. Jeong, K. No, H. Kwon, S.‐G. Lee, B. Joung, K. J. Lee, Adv. Mater. 2014, 26, 4880. 24740465 10.1002/adma.201400562

[43] D. Ponnamma, J.‐J. Cabibihan, M. Rajan, S. S. Pethaiah, K. Deshmukh, J. P. Gogoi, S. K. K. Pasha, M. B. Ahamed, J. Krishnegowda, B. N. Chandrashekar, A. R. Polu, C. Cheng, Mater. Sci. Eng., C 2019, 98, 1210. 10.1016/j.msec.2019.01.081 30813004

[44] W.‐S. Jung, Y.‐H. Do, M.‐G. Kang, C.‐Y. Kang, Curr. Appl. Phys. 2013, 13, S131.

[45] A. Jain, K. J. Prashanth, A. K. Sharma, A. Jain, P. N. Rashmi, Polym. Eng. Sci. 2015, 55, 1589.

[46] Y. Qi, J. Kim, T. D. Nguyen, B. Lisko, P. K. Purohit, M. C. McAlpine, Nano Lett. 2011, 11, 1331. 21322604 10.1021/nl104412b

[47] Y. Chang, B. Yin, Y. Qiu, H. Zhang, J. Lei, Y. Zhao, Y. Luo, L. Hu, J. Mater. Sci.: Mater. Electron. 2016, 27, 3773.

[48] J.‐H. Bae, S.‐H. Chang, Compos. Struct. 2015, 131, 1090.

[49] R. L. Hadimani, D. V. Bayramol, N. Sion, T. Shah, L. Qian, S. Shi, E. Siores, Smart Mater. Struct. 2013, 22, 075017.

[50] G. Zhu, R. Yang, S. Wang, Z. L. Wang, Nano Lett. 2010, 10, 3151. 20698630 10.1021/nl101973h

[51] S. Xu, B. J. Hansen, Z. L. Wang, Nat. Commun. 2010, 1, 93. 20981021 10.1038/ncomms1098

[52] S.‐H. Shin, Y.‐H. Kim, J.‐Y. Jung, M. H. Lee, J. Nah, Nanotechnology 2014, 25, 485401. 25392282 10.1088/0957-4484/25/48/485401

[53] P. Curie, J. Curie, Bulletin of the Mineralogical Society of France 1880, 3, 89

[54] C. Jean‐Mistral, S. Basrour, J.‐J. Chaillout, Smart Mater. Struct. 2010, 19, 085012.

[55] V. V. Kochervinskii, Crystallogr. Rep. 2003, 48, 649.

[56] T. Furukawa, IEEE Trans. Electr. Insul. 1989, 24, 375.

[57] “Piezoelectric Crystal Classes,” can be found under https://www.staff.ncl.ac.uk/j.p.goss/symmetry/PP_Piezo.html.

[58] N. A. Shepelin, A. M. Glushenkov, V. C. Lussini, P. J. Fox, G. W. Dicinoski, J. G. Shapter, A. V. Ellis, Energy Environ. Sci. 2019, 12, 1143.

[59] G. D. Jones, R. A. Assink, T. R. Dargaville, P. M. Chaplya, R. L. Clough, J. M. Elliott, J. W. Martin, D. M. Mowery, M. C. Celina, Sandia National Laboratories, Albuquerque, NM 2005.

[60] J. Y.‐H. Kim, A. Cheng, Y.‐C. Tai, in IEEE 24th Int. Conf. Micro Electro Mechanical Systems, IEEE, Piscataway, NJ 2011, pp. 473–476.

[61] C. Park, Z. Ounaies, K. E. Wise, J. S. Harrison, Polymer 2004, 45, 5417.

[62] C. Li, P.‐M. Wu, S. Lee, A. Gorton, M. J. Schulz, C. H. Ahn, J. Microelectromech. Syst. 2008, 17, 334.

[63] H. Kim, F. Torres, Y. Wu, D. Villagran, Y. Lin, T.‐L. Tseng, Smart Mater. Struct. 2017, 26, 085027.

[64] X. Ren, H. Fan, Y. Zhao, Z. Liu, ACS Appl. Mater. Interfaces 2016, 8, 26190. 27611593 10.1021/acsami.6b04497

[65] C. Wan, C. R. Bowen, J. Mater. Chem. A 2017, 5, 3091.

[66] S. K. Ghosh, A. Biswas, S. Sen, C. Das, K. Henkel, D. Schmeisser, D. Mandal, Nano Energy 2016, 30, 621.

[67] S. K. Ghosh, D. Mandal, Nano Energy 2016, 28, 356.

[68] S. K. Ghosh, D. Mandal, Appl. Phys. Lett. 2016, 109, 103701.

[69] T. Ikeda, Fundamentals of Piezoelectricity, Oxford University Press, Oxford 1990.

[70] S. M. Hatch, J. Briscoe, S. Dunn, Thin Solid Films 2013, 531, 404.

[71] S. Dunn, J. Appl. Phys. 2003, 94, 5964.

[72] S. Kaps, S. Bhowmick, J. Gröttrup, V. Hrkac, D. Stauffer, H. Guo, O. L. Warren, J. Adam, L. Kienle, A. M. Minor, R. Adelung, Y. K. Mishra, ACS Omega 2017, 2, 2985. 31457633 10.1021/acsomega.7b00041 PMC6640942

[73] Z. L. Wang, Science 2006, 312, 242. 16614215 10.1126/science.1124005

[74] Z. L. Wang, MRS Bull. 2007, 32, 109.

[75] Z. L. Wang, J. Phys.: Condens. Matter 2004, 16, R829.

[76] Z. L. Wang, Mater. Sci. Eng., R 2009, 64, 33.

[77] Prof. Dr. Hadis Morkoç, Ümit Özgür https://www.wiley.com/en-us/Zinc%2BOxide%3A%2BFundamentals%2C%2BMaterials%2Band%2BDevice%2BTechnology-p-9783527408139, 2009, Wiley‐VCH.

[78] “eFunda: Properties of Piezo Material Zinc Oxide,” can be found under https://www.efunda.com/materials/piezo/material_data/matdata_output.cfm?Material_ID=ZnO.

[79] D.‐F. Zhang, L.‐D. Sun, C.‐J. Jia, Z.‐G. Yan, L.‐P. You, C.‐H. Yan, J. Am. Chem. Soc. 2005, 127, 13492. 16190701 10.1021/ja054771k

[80] M.‐P. Lu, J. Song, M.‐Y. Lu, M.‐T. Chen, Y. Gao, L.‐J. Chen, Z. L. Wang, Nano Lett. 2009, 9, 1223. 19209870 10.1021/nl900115y

[81] R. Yang, Y. Qin, C. Li, G. Zhu, Z. L. Wang, Nano Lett. 2009, 9, 1201. 19203203 10.1021/nl803904b

[82] R. Yang, Y. Qin, L. Dai, Z. L. Wang, Nat. Nanotechnol. 2009, 4, 34. 19119280 10.1038/nnano.2008.314

[83] R. Yang, Y. Qin, C. Li, L. Dai, Z. L. Wang, Appl. Phys. Lett. 2009, 94, 022905.

[84] M.‐Y. Choi, D. Choi, M.‐J. Jin, I. Kim, S.‐H. Kim, J.‐Y. Choi, S. Y. Lee, J. M. Kim, S.‐W. Kim, Adv. Mater. 2009, 21, 2185.

[85] D. Choi, M.‐Y. Choi, H.‐J. Shin, S.‐M. Yoon, J.‐S. Seo, J.‐Y. Choi, S. Y. Lee, J. M. Kim, S.‐W. Kim, J. Phys. Chem. C 2010, 114, 1379.

[86] D. Choi, M.‐Y. Choi, W. M. Choi, H.‐J. Shin, H.‐K. Park, J.‐S. Seo, J. Park, S.‐M. Yoon, S. J. Chae, Y. H. Lee, S.‐W. Kim, J.‐Y. Choi, S. Y. Lee, J. M. Kim, Adv. Mater. 2010, 22, 2187. 20376853 10.1002/adma.200903815

[87] Y. Hu, Y. Zhang, C. Xu, L. Lin, R. L. Snyder, Z. L. Wang, Nano Lett. 2011, 11, 2572. 21604749 10.1021/nl201505c

[88] Y. Hu, L. Lin, Y. Zhang, Z. L. Wang, Adv. Mater. 2012, 24, 110. 22057731 10.1002/adma.201103727

[89] G. Zhu, A. C. Wang, Y. Liu, Y. Zhou, Z. L. Wang, Nano Lett. 2012, 12, 3086. 22594588 10.1021/nl300972f

[90] Y. Hu, Y. Zhang, C. Xu, G. Zhu, Z. L. Wang, Nano Lett. 2010, 10, 5025. 21047124 10.1021/nl103203u

[91] J. Briscoe, E. Bilotti, S. Dunn, Appl. Phys. Lett. 2012, 101, 093902.

[92] J. Briscoe, S. Dunn, Nano Energy 2015, 14, 15.

[93] N. Jalali, P. Woolliams, M. Stewart, P. M. Weaver, M. G. Cain, S. Dunn, J. Briscoe, J. Mater. Chem. A 2014, 2, 10945.

[94] S. Xu, Y. Qin, C. Xu, Y. Wei, R. Yang, Z. L. Wang, Nat. Nanotechnol. 2010, 5, 366. 20348913 10.1038/nnano.2010.46

[95] Y. Qin, X. Wang, Z. L. Wang, Nature 2008, 451, 809. 18273015 10.1038/nature06601

[96] S. Bai, L. Zhang, Q. Xu, Y. Zheng, Y. Qin, Z. L. Wang, Nano Energy 2013, 2, 749.

[97] Y. Qiu, H. Zhang, L. Hu, D. Yang, L. Wang, B. Wang, J. Ji, G. Liu, X. Liu, J. Lin, F. Li, S. Han, Nanoscale 2012, 4, 6568. 22971814 10.1039/c2nr31031g

[98] H. Kim, S. M. Kim, H. Son, H. Kim, B. Park, J. Ku, J. I. Sohn, Y. J. Park, Energy Environ. Sci 2012, 5, 8932.

[99] D. Tamvakos, S. Lepadatu, V.‐A. Antohe, A. Tamvakos, P. M. Weaver, L. Piraux, M. G. Cain, D. Pullini, Appl. Surf. Sci. 2015, 356, 1214.

[100] H. Jaffe, D. A. Berlincourt, Proc. IEEE 1965, 53, 1372.

[101] L. P. Schuler, M. M. Alkaisi, P. Miller, R. J. Reeves, A. Markwitz, Jpn. J. Appl. Phys. 2005, 44, 7555.

[102] M.‐H. Zhao, Z.‐L. Wang, S. X. Mao, Nano Lett. 2004, 4, 587.

[103] E. Broitman, M. Y. Soomro, J. Lu, M. Willander, L. Hultman, Phys. Chem. Chem. Phys. 2013, 15, 11113. 23722480 10.1039/c3cp50915j

[104] D. A. Scrymgeour, T. L. Sounart, N. C. Simmons, J. W. P. Hsu, J. Appl. Phys. 2007, 101, 014316.

[105] H. J. Fan, W. Lee, R. Hauschild, M. Alexe, G. Le Rhun, R. Scholz, A. Dadgar, K. Nielsch, H. Kalt, A. Krost, M. Zacharias, U. Gösele, Small 2006, 2, 561. 17193086 10.1002/smll.200500331

[106] K.‐H. Kim, B. Kumar, K. Y. Lee, H.‐K. Park, J.‐H. Lee, H. H. Lee, H. Jun, D. Lee, S.‐W. Kim, Sci. Rep. 2013, 3, 2017. 23774788 10.1038/srep02017 PMC3684811

[107] M. K. Gupta, J.‐H. Lee, K. Y. Lee, S.‐W. Kim, ACS Nano 2013, 7, 8932. 24004103 10.1021/nn403428m

[108] B. Kumar, K. Y. Lee, H.‐K. Park, S. J. Chae, Y. H. Lee, S.‐W. Kim, ACS Nano 2011, 5, 4197. 21495657 10.1021/nn200942s

[109] B. Saravanakumar, S.‐J. Kim, J. Phys. Chem. C 2014, 118, 8831.

[110] M. Fortunato, C. R. Chandraiahgari, G. De Bellis, P. Ballirano, P. Soltani, S. Kaciulis, L. Caneve, F. Sarto, M. S. Sarto, IEEE Trans. Nanotechnol. 2018, 17, 311.

[111] Y. Saito, H. Takao, T. Tani, T. Nonoyama, K. Takatori, T. Homma, T. Nagaya, M. Nakamura, Nature 2004, 432, 84. 15516921 10.1038/nature03028

[112] T. R. Shrout, S. J. Zhang, J. Electroceram. 2007, 19, 113.

[113] T. Takenaka, H. Nagata, J. Eur. Ceram. Soc. 2005, 25, 2693.

[114] L. X. Zhang, W. Chen, X. Ren, Appl. Phys. Lett. 2004, 85, 5658.

[115] N. Cui, W. Wu, Y. Zhao, S. Bai, L. Meng, Y. Qin, Z. L. Wang, Nano Lett. 2012, 12, 3701. 22681509 10.1021/nl301490q

[116] X. Chen, S. Xu, N. Yao, W. Xu, Y. Shi, Appl. Phys. Lett. 2009, 94, 253113.

[117] S. Xu, Y. Shi, S.‐G. Kim, Nanotechnology 2006, 17, 4497.

[118] X. Chen, S. Xu, N. Yao, Y. Shi, Nano Lett. 2010, 10, 2133. 20499906 10.1021/nl100812k

[119] W. Wu, S. Bai, M. Yuan, Y. Qin, Z. L. Wang, T. Jing, ACS Nano 2012, 6, 6231. 22713250 10.1021/nn3016585

[120] L. Gu, N. Cui, L. Cheng, Q. Xu, S. Bai, M. Yuan, W. Wu, J. Liu, Y. Zhao, F. Ma, Y. Qin, Z. L. Wang, Nano Lett. 2013, 13, 91. 23205727 10.1021/nl303539c

[121] Y. Lin, Y. Liu, H. A. Sodano, Appl. Phys. Lett. 2009, 95, 122901.

[122] I.‐J. No, D.‐Y. Jeong, S. Lee, S.‐H. Kim, J.‐W. Cho, P.‐K. Shin, Microelectron. Eng. 2013, 110, 282.

[123] S. Xu, G. Poirier, N. Yao, Nano Lett. 2012, 12, 2238. 22494473 10.1021/nl204334x

[124] H. B. Kang, J. Chang, K. Koh, L. Lin, Y. S. Cho, ACS Appl. Mater. Interfaces 2014, 6, 10576. 24919853 10.1021/am502234q

[125] K.‐I. Park, M. Lee, Y. Liu, S. Moon, G.‐T. Hwang, G. Zhu, J. E. Kim, S. O. Kim, D. K. Kim, Z. L. Wang, K. J. Lee, Adv. Mater. 2012, 24, 2999. 22549998 10.1002/adma.201200105

[126] A. Koka, Z. Zhou, H. A. Sodano, Energy Environ. Sci. 2014, 7, 288.

[127] A. Koka, Z. Zhou, H. Tang, H. A. Sodano, Nanotechnology 2014, 25, 375603. 25148612 10.1088/0957-4484/25/37/375603

[128] B. Liu, B. Lu, X. Chen, X. Wu, S. Shi, L. Xu, Y. Liu, F. Wang, X. Zhao, W. Shi, J. Mater. Chem. A 2017, 5, 23634.

[129] W. S. Su, Y. F. Chen, C. L. Hsiao, L. W. Tu, Appl. Phys. Lett. 2007, 90, 063110.

[130] N. Gogneau, P. Chrétien, E. Galopin, S. Guilet, L. Travers, J.‐C. Harmand, F. Houzé, Appl. Phys. Lett. 2014, 104, 213105.

[131] N. C. Jamond, P. Chrétien, F. Houzé, L. Lu, L. Largeau, O. Maugain, L. Travers, J.‐C. Harmand, F. Glas, E. Lefeuvre, M. Tchernycheva, N. C. Gogneau, Nanotechnology 2016, 27, 325403. 27363777 10.1088/0957-4484/27/32/325403

[132] J. Song, J. Zhou, Z. L. Wang, Nano Lett. 2006, 6, 1656. 16895352 10.1021/nl060820v

[133] X. Wang, J. Zhou, J. Song, J. Liu, N. Xu, Z. L. Wang, Nano Lett. 2006, 6, 2768. 17163703 10.1021/nl061802g

[134] X. Cai, T. Lei, D. Sun, L. Lin, RSC Adv. 2017, 7, 15382.

[135] Q. M. Zhang, V. Bharti, G. Kavarnos, in Encycl. Smart Mater., , American Cancer Society, 2002.

[136] J. S. Harrison, Z. Ounaies, in Encycl. Polym. Sci. Technol., American Cancer Society, 2002.

[137] E. Fukada, IEEE Trans. Ultrason., Ferroelectr., Freq. Control 2000, 47, 1277. 18238673 10.1109/58.883516

[138] J. Kim, S. Yun, Z. Ounaies, Macromolecules 2006, 39, 4202.

[139] Fukada, IEEE Trans. Dielectr. Electr. Insul. 2006, 13, 1110.

[140] G. M. Atkinson, R. E. Pearson, Z. Ounaies, C. Park, J. S. Harrison, S. Dogan, J. A. Midkiff, in TRANSDUCERS 2003–12th Int. Conf. Solid‐State Sens. Actuators Microsyst. Dig. Tech. Pap., 1, Institute Of Electrical And Electronics Engineers Inc., Boston, USA 2003, pp. 782–785.

[141] H. Kawai, Jpn. J. Appl. Phys. 1969, 8, 975.

[142] E. Fukada, Jpn. J. Appl. Phys. 1998, 37, 2775.

[143] J. Gomes, J. S. Nunes, V. Sencadas, S. Lanceros‐Mendez, Smart Mater. Struct. 2010, 19, 065010.

[144] H. S. Nalwa, Ferroelectric Polymers : Chemistry: Physics, and Applications, CRC Press, Boca Raton, FL 1995.

[145] T. Kaura, R. Nath, M. M. Perlman, J. Phys. D: Appl. Phys. 1991, 24, 1848.

[146] K. Koga, H. Ohigashi, J. Appl. Phys. 1986, 59, 2142.

[147] P. Martins, J. S. Nunes, G. Hungerford, D. Miranda, A. Ferreira, V. Sencadas, S. Lanceros‐Méndez, Phys. Lett. A 2009, 373, 177.

[148] A. J. Lovinger, Science 1983, 220, 1115. 17818472 10.1126/science.220.4602.1115

[149] J. L. Lutkenhaus, K. McEnnis, A. Serghei, T. P. Russell, Macromolecules 2010, 43, 3844.

[150] P. Martins, A. C. Lopes, S. Lanceros‐Mendez, Prog. Polym. Sci. 2014, 39, 683.

[151] H. Xu, J. Appl. Polym. Sci. 2001, 80, 2259.

[152] Z. Li, Y. Wang, Z.‐Y. Cheng, Appl. Phys. Lett. 2006, 88, 062904.

[153] Piezoelectric and Acoustic Materials for Transducer Applications, (Eds: A. Safari, E. K. Akdoğan), Springer US, Boston, MA 2008.

[154] B. Ameduri, Chem. Rev. 2009, 109, 6632. 19731907 10.1021/cr800187m

[155] Y. Huan, Y. Liu, Y. Yang, Y. Wu, J. Appl. Polym. Sci. 2007, 104, 858.

[156] W. Künstler, M. Wegener, M. Seiß, R. Gerhard‐Multhaupt, Appl. Phys. A 2001, 73, 641.

[157] B. Neese, Y. Wang, B. Chu, K. Ren, S. Liu, Q. M. Zhang, C. Huang, J. West, Appl. Phys. Lett. 2007, 90, 242917.

[158] M. Aldas, G. Boiteux, G. Seytre, Z. Ghallabi, in 2010 10th IEEE Int. Conf. Solid Dielectr., IEEE, Potsdam, Germany 2010, pp. 1–4.

[159] P. Frübing, A. Kremmer, R. Gerhard‐Multhaupt, A. Spanoudaki, P. Pissis, J. Chem. Phys. 2006, 125, 214701. 17166034 10.1063/1.2360266

[160] Y. Takase, J. W. Lee, J. I. Scheinbeim, B. A. Newman, Macromolecules 1991, 24, 6644.

[161] F. Bernard, L. Gimeno, B. Viala, B. Gusarov, O. Cugat, Proceedings 2017, 1, 335.

[162] S. Hikosaka, H. Ishikawa, Y. Ohki, Electron. Commun. Jpn. 2011, 94, 1.

[163] S. Rajala, T. Siponkoski, E. Sarlin, M. Mettänen, M. Vuoriluoto, A. Pammo, J. Juuti, O. J. Rojas, S. Franssila, S. Tuukkanen, ACS Appl. Mater. Interfaces 2016, 8, 15607. 27232271 10.1021/acsami.6b03597

[164] J. Kim, S. Yun, S. K. Mahadeva, K. Yun, S. Y. Yang, M. Maniruzzaman, Sensors 2010, 10, 1473. 22294882 10.3390/s100301473 PMC3264434

[165] L. Csoka, I. C. Hoeger, O. J. Rojas, I. Peszlen, J. J. Pawlak, P. N. Peralta, ACS Macro Lett. 2012, 1, 867. 10.1021/mz300234a 35607134

[166] S. H. Hassan, L. H. Voon, T. S. Velayutham, L. Zhai, H. C. Kim, J. Kim, J. Renewable Mater. 2018, 6, 1.

[167] L. Lebrun, D. Guyomar, B. Guiffard, P.‐J. Cottinet, C. Putson, Sens. Actuators, A 2009, 153, 251.

[168] K. Wongtimnoi, B. Guiffard, A. B.‐V. de Moortèle, L. Seveyrat, C. Gauthier, J.‐Y. Cavaillé, Compos. Sci. Technol. 2011, 71, 885.

[169] M. Morimoto, Y. Koshiba, M. Misaki, K. Ishida, Appl. Phys. Express 2015, 8, 101501.

[170] V. K. Thakur, M.‐F. Lin, E. J. Tan, P. S. Lee, J. Mater. Chem. 2012, 22, 5951.

[171] V. K. Thakur, E. J. Tan, M.‐F. Lin, P. S. Lee, J. Mater. Chem. 2011, 21, 3751.

[172] V. K. Thakur, E. J. Tan, M.‐F. Lin, P. S. Lee, Polym. Chem. 2011, 2, 2000.

[173] S. Chen, A. Skordos, V. K. Thakur, Mater. Today Chem. 2020, 17, 100304. 10.1016/j.mtchem.2020.100300 PMC7254035 32835154

[174] I. Katsouras, K. Asadi, M. Li, T. B. van Driel, K. S. Kjær, D. Zhao, T. Lenz, Y. Gu, P. W. M. Blom, D. Damjanovic, M. M. Nielsen, D. M. de Leeuw, Nat. Mater. 2016, 15, 78. 26436342 10.1038/nmat4423

[175] H. M. G. Correia, M. M. D. Ramos, Comput. Mater. Sci. 2005, 33, 224.

[176] A. J. Lovinger, Macromolecules 1982, 15, 40.

[177] R. Gregorio Jr., M. Cestari, J. Polym. Sci., Part B: Polym. Phys. 1994, 32, 859.

[178] M. Benz, W. B. Euler, J. Appl. Polym. Sci. 2003, 89, 1093.

[179] D. R. Dillon, K. K. Tenneti, C. Y. Li, F. K. Ko, I. Sics, B. S. Hsiao, Polymer 2006, 47, 1678.

[180] A. Salimi, A. A. Yousefi, Polym. Test. 2003, 22, 699.

[181] R. G. Kepler, R. A. Anderson, J. Appl. Phys. 1978, 49, 4490.

[182] V. Sencadas, R. Gregorio Jr., S. Lanceros‐Méndez, J. Macromol. Sci., Part B: Phys. 2009, 48, 514.

[183] V. Sencadas, M. V. Moreira, S. Lanceros‐Méndez, A. S. Pouzada, R. Gregório Filho, Materials Science Forum 2006, 514‐516, 872.

[184] H. Pan, B. Na, R. Lv, C. Li, J. Zhu, Z. Yu, J. Polym. Sci., Part B: Polym. Phys. 2012, 50, 1433.

[185] T. Hattori, M. Kanaoka, H. Ohigashi, J. Appl. Phys. 1996, 79, 2016.

[186] W. W. Doll, J. B. Lando, J. Macromol. Sci., Part B: Phys. 1970, 4, 889.

[187] C. Ribeiro, V. Sencadas, J. L. G. Ribelles, S. Lanceros‐Méndez, Soft Mater. 2010, 8, 274.

[188] J. Zheng, A. He, J. Li, C. C. Han, Macromol. Rapid Commun. 2007, 28, 2159.

[189] A. Lund, B. Hagström, J. Appl. Polym. Sci. 2011, 120, 1080.

[190] A. Baji, Y.‐W. Mai, X. Du, S.‐C. Wong, Macromol. Mater. Eng. 2012, 297, 209.

[191] G. Zhong, L. Zhang, R. Su, K. Wang, H. Fong, L. Zhu, Polymer 2011, 52, 2228.

[192] Y. Jiang, Y. Ye, J. Yu, Z. Wu, W. Li, J. Xu, G. Xie, Polym. Eng. Sci. 2007, 47, 1344.

[193] A. Gradys, P. Sajkiewicz, S. Adamovsky, A. Minakov, C. Schick, Thermochim. Acta 2007, 461, 153.

[194] A. Ferreira, P. Costa, H. Carvalho, J. M. Nobrega, V. Sencadas, S. Lanceros‐Mendez, J. Polym. Res. 2011, 18, 1653.

[195] V. Sencadas, R. G. Filho, S. Lanceros‐Mendez, J. Non‐Cryst. Solids 2006, 352, 2226.

[196] F. Mokhtari, M. Latifi, M. Shamshirsaz, J. Text. Inst. 2015, 107, 1037.

[197] J. Chang, M. Dommer, C. Chang, L. Lin, Nano Energy 2012, 1, 356.

[198] B. K. Panigrahi, D. Sitikantha, A. Bhuyan, H. S. Panda, K. Mohanta, Mater. Today: Proc. 2021, 41, 335.

[199] C. Chang, K. Limkrailassiri, L. Lin, Appl. Phys. Lett. 2008, 93, 123111.

[200] D. Sun, C. Chang, S. Li, L. Lin, Nano Lett. 2006, 6, 839. 16608294 10.1021/nl0602701

[201] C. Chang, V. H. Tran, J. Wang, Y.‐K. Fuh, L. Lin, Nano Lett. 2010, 10, 726. 20099876 10.1021/nl9040719

[202] Y. Xin, X. Qi, H. Tian, C. Guo, X. Li, J. Lin, C. Wang, Mater. Lett. 2016, 164, 136.

[203] J. Pu, X. Yan, Y. Jiang, C. Chang, L. Lin, Sens. Actuators, A 2010, 164, 131.

[204] J. Chang, L. Lin, in 2011 16th Int. Solid‐State Sens. Actuators Microsyst. Conf., IEEE, China 2011, pp. 747–750.

[205] Z. H. Liu, C. T. Pan, L. W. Lin, J. C. Huang, Z. Y. Ou, Smart Mater. Struct. 2014, 23, 025003.

[206] C.‐T. Pan, C.‐K. Yen, S.‐Y. Wang, Y.‐C. Lai, L. Lin, J. C. Huang, S.‐W. Kuo, RSC Adv. 2015, 5, 85073.

[207] M. Kanik, O. Aktas, H. S. Sen, E. Durgun, M. Bayindir, ACS Nano 2014, 8, 9311. 25133594 10.1021/nn503269b

[208] S. Cha, S. M. Kim, H. Kim, J. Ku, J. I. Sohn, Y. J. Park, B. G. Song, M. H. Jung, E. K. Lee, B. L. Choi, J. J. Park, Z. L. Wang, J. M. Kim, K. Kim, Nano Lett. 2011, 11, 5142. 22107106 10.1021/nl202208n

[209] Y. Mao, P. Zhao, G. McConohy, H. Yang, Y. Tong, X. Wang, Adv. Energy Mater. 2014, 4, 1301624.

[210] D. Chen, T. Sharma, J. X. J. Zhang, Sens. Actuators, A 2014, 216, 196.

[211] S. Chen, X. Li, K. Yao, F. E. H. Tay, A. Kumar, K. Zeng, Polymer 2012, 53, 1404.

[212] L. Jin, S. Ma, W. Deng, C. Yan, T. Yang, X. Chu, G. Tian, D. Xiong, J. Lu, W. Yang, Nano Energy 2018, 50, 632.

[213] J. Zhang, Z. Fang, C. Shu, J. Zhang, Q. Zhang, C. Li, Sens. Actuators, A 2017, 262, 123.

[214] B. Bera, M. D. Sarkar, IOSR J. Polym. Text. Eng. 2017, 4, 1.

[215] A. Talbourdet, F. Rault, G. Lemort, C. Cochrane, E. Devaux, C. Campagne, Smart Mater. Struct. 2018, 27, 075010.

[216] T. Yagi, M. Tatemoto, Polym. J. 1979, 11, 429.

[217] K. Aimi, S. Ando, P. Avalle, R. K. Harris, Polymer 2004, 45, 2281.

[218] T. Furukawa, M. Date, E. Fukada, Y. Tajitsu, A. Chiba, Jpn. J. Appl. Phys. 1980, 19, L109.

[219] Y. Higashihata, J. Sako, T. Yagi, Ferroelectrics 1981, 32, 85.

[220] T. Furukawa, G. E. Johnson, H. E. Bair, Y. Tajitsu, A. Chiba, E. Fukada, Ferroelectrics 1981, 32, 61.

[221] K. Tashiro, H. Tadokoro, M. Kobayashi, Ferroelectrics 1981, 32, 167.

[222] L. Zhang, S. Oh, T. Wong, C. Y. Tan, K. Yao, IEEE Tans. Ultrason., Ferroelectr., Freq. Control 2013, 60, 2013. 10.1109/TUFFC.2013.2786 24658732

[223] Z. Pi, J. Zhang, C. Wen, Z. Zhang, D. Wu, Nano Energy 2014, 7, 33.

[224] F. Oliveira, Y. Leterrier, J.‐A. Månson, O. Sereda, A. Neels, A. Dommann, D. Damjanovic, J. Polym. Sci., Part B: Polym. Phys. 2014, 52, 496.

[225] J.‐H. Lee, H.‐J. Yoon, T. Y. Kim, M. K. Gupta, J. H. Lee, W. Seung, H. Ryu, S.‐W. Kim, Adv. Funct. Mater. 2015, 25, 3203.

[226] V. Cauda, S. Stassi, K. Bejtka, G. Canavese, ACS Appl. Mater. Interfaces 2013, 5, 6430. 23777739 10.1021/am4016878

[227] V. Bhavanasi, D. Y. Kusuma, P. S. Lee, Adv. Energy Mater. 2014, 4, 1400723.

[228] D. Chen, K. Chen, K. Brown, A. Hang, J. X. J. Zhang, Appl. Phys. Lett. 2017, 110, 153902.

[229] A. Aliane, M. Benwadih, B. Bouthinon, R. Coppard, F. Domingues‐Dos, Santos, A. Daami, Org. Electron. 2015, 25, 92.

[230] S. Dey, M. Purahmad, S. S. Ray, A. L. Yarin, M. Dutta, in Nanotechnol. Mater. Devices Conf., IEEE 2012, pp. 21–24.

[231] L. Persano, C. Dagdeviren, Y. Su, Y. Zhang, S. Girardo, D. Pisignano, Y. Huang, J. A. Rogers, Nat. Commun. 2013, 4, 1633. 23535654 10.1038/ncomms2639

[232] N. K. Kalfoglou, H. L. Williams, J. Appl. Polym. Sci. 1973, 17, 3367.

[233] M. Latour, H. A. Dorra, Ferroelectrics 1982, 44, 197.

[234] B. Améduri, B. Boutevin, G. Kostov, Prog. Polym. Sci. 2001, 26, 105.

[235] M. Apostolo, V. Arcella, G. Storti, M. Morbidelli, Macromolecules 1999, 32, 989.

[236] T. S. Ahmed, J. M. DeSimone, G. W. Roberts, Macromolecules 2006, 39, 15.

[237] Fluoroelastomers Handbook, A volume in plastics design library, Science Direct, 2005.

[238] S. Mishra, L. Unnikrishnan, S. K. Nayak, S. Mohanty, Macromol. Mater. Eng. 2019, 304, 1800463.

[239] C. Zhao, J. Zhang, Z. L. Wang, K. Ren, Adv. Sustainable Syst. 2017, 1, 1700068.

[240] J. Zhu, L. Jia, R. Huang, J. Mater. Sci.: Mater. Electron. 2017, 28, 12080.

[241] S. J. Lee, A. P. Arun, K. J. Kim, Mater. Lett. 2015, 148, 58.

[242] D. Koyama, K. Nakamura, Appl. Acoust. 2010, 71, 439.

[243] T. Hattori, Y. Takahashi, M. Iijima, E. Fukada, J. Appl. Phys. 1996, 79, 1713.

[244] E. Fukada, M. Date, T. Ochiai, S. Koyama, Y. Sasaki, in 10th International Symposium on Electrets (ISE 10). Proceedings (Cat. No.99 CH36256), IEEE, Athens, Greece 1999, pp. 655–658.

[245] M. J. Moody, C. W. Marvin, G. R. Hutchison, J. Mater. Chem. C 2016, 4, 4387.

[246] W. Wang, Y. Zheng, X. Jin, Y. Sun, B. Lu, H. Wang, J. Fang, H. Shao, T. Lin, Nano Energy 2019, 56, 588.

[247] T. Cai, Y. Yang, E. Bi, React. Funct. Polym. 2020, 154, 104638.

[248] B. Gonzalo, J. L. Vilas, T. Breczewski, M. A. Pérez‐Jubindo, M. R. D. L. Fuente, M. Rodriguez, L. M. León, J. Polym. Sci., Part A: Polym. Chem. 2009, 47, 722.

[249] N. Wu, X. Cheng, Q. Zhong, J. Zhong, W. Li, B. Wang, B. Hu, J. Zhou, Adv. Funct. Mater. 2015, 25, 4788.

[250] Prateek, V. K. Thakur, R. K. Gupta, Chem. Rev. 2016, 116, 4260. 27040315 10.1021/acs.chemrev.5b00495

[251] Y. Zhao, Q. Liao, G. Zhang, Z. Zhang, Q. Liang, X. Liao, Y. Zhang, Nano Energy 2015, 11, 719.

[252] F. Wang, Y.‐W. Mai, D. Wang, R. Ding, W. Shi, Sens. Actuators, A 2015, 233, 195.

[253] R. Li, Z. Zhao, Z. Chen, J. Pei, Mater. Express 2017, 7, 536.

[254] J. Fu, Y. Hou, X. Gao, M. Zheng, M. Zhu, Nano Energy 2018, 52, 391.

[255] F. N. Meyers, K. J. Loh, J. S. Dodds, A. Baltazar, Nanotechnology 2013, 24, 185501. 23579369 10.1088/0957-4484/24/18/185501

[256] C.‐Y. Chen, J.‐H. Huang, J. Song, Y. Zhou, L. Lin, P.‐C. Huang, Y. Zhang, C.‐P. Liu, J.‐H. He, Z. L. Wang, ACS Nano 2011, 5, 6707. 21774517 10.1021/nn202251m

[257] J. S. Dodds, F. N. Meyers, K. J. Loh, IEEE Sens. J. 2012, 12, 1889.

[258] H. Chen, X. Dong, T. Zeng, Z. Zhou, H. Yang, Ceram. Int. 2007, 33, 1369.

[259] B. Wu, H. Wu, J. Wu, D. Xiao, J. Zhu, S. J. Pennycook, J. Am. Chem. Soc. 2016, 138, 15459. 27933925 10.1021/jacs.6b09024

[260] N. Abzan, M. Kharaziha, S. Labbaf, Mater. Des. 2019, 167, 107636.

[261] R. K. Layek, S. Samanta, D. P. Chatterjee, A. K. Nandi, Polymer 2010, 51, 5846.

[262] H. M. Ning, N. Hu, T. Kamata, J. H. Qiu, X. Han, L. M. Zhou, C. Chang, Y. Liu, L. K. Wu, J. H. Qiu, H. L. Ji, W. X. Wang, Y. Zemba, S. Atobe, Y. Li, Alamusi, H. Fukunaga, Smart Mater. Struct. 2013, 22, 065011.

[263] G. H. Kim, S. M. Hong, Y. Seo, Phys. Chem. Chem. Phys. 2009, 11, 10506. 19890538 10.1039/b912801h

[264] L. Wu, W. Yuan, N. Hu, Z. Wang, C. Chen, J. Qiu, J. Ying, Y. Li, J. Phys. D: Appl. Phys. 2014, 47, 135302.

[265] J. Cai, N. Hu, L. Wu, Y. Liu, Y. Li, H. Ning, X. Liu, L. Lin, Composites, Part A 2019, 121, 223.

[266] H. H. Singh, S. Singh, N. Khare, Compos. Sci. Technol. 2017, 149, 127.

[267] D. Ponnamma, A. Erturk, H. Parangusan, K. Deshmukh, M. B. Ahamed, M. A. A. Al‐Maadeed, Emergent Mater. 2018, 1, 55.

[268] L. Yang, M. Cheng, W. Lyu, M. Shen, J. Qiu, H. Ji, Q. Zhao, Composites, Part A 2018, 107, 536.

[269] S. Lotfian, C. Giraudmaillet, A. Yoosefinejad, V. K. Thakur, H. Y. Nezhad, ACS Omega 2018, 3, 8891. 31459021 10.1021/acsomega.8b00940 PMC6644386

[270] S.‐H. Shin, Y.‐H. Kim, M. H. Lee, J.‐Y. Jung, J. Nah, ACS Nano 2014, 8, 2766. 24517314 10.1021/nn406481k

[271] S. Bodkhe, G. Turcot, F. P. Gosselin, D. Therriault, ACS Appl. Mater. Interfaces 2017, 9, 20833. 28553704 10.1021/acsami.7b04095

[272] S. Bodkhe, P. S. M. Rajesh, F. P. Gosselin, D. Therriault, ACS Appl. Energy Mater. 2018, 1, 2474.

[273] S. Siddiqui, D.‐I. Kim, L. T. Duy, M. T. Nguyen, S. Muhammad, W.‐S. Yoon, N.‐E. Lee, Nano Energy 2015, 15, 177.

[274] S. Siddiqui, D.‐I. Kim, E. Roh, L. T. Duy, T. Q. Trung, M. T. Nguyen, N.‐E. Lee, Nano Energy 2016, 30, 434.

[275] J. Nunes‐Pereira, V. Sencadas, V. Correia, V. F. Cardoso, W. Han, J. G. Rocha, S. Lanceros‐Méndez, Composites, Part B 2015, 72, 130.

[276] J. Nunes‐Pereira, V. Sencadas, V. Correia, J. G. Rocha, S. Lanceros‐Méndez, Sens. Actuators, A 2013, 196, 55.

[277] M. Sahu, S. Hajra, K. Lee, P. L. Deepti, K. Mistewicz, H. J. Kim, Crystals 2021, 11, 85.

[278] C. Lee, D. Wood, D. Edmondson, D. Yao, A. E. Erickson, C. T. Tsao, R. A. Revia, H. Kim, M. Zhang, Ceram. Int. 2016, 42, 2734.

[279] K. Kakimoto, K. Fukata, H. Ogawa, Sens. Actuators, A 2013, 200, 21.

[280] X. Guan, B. Xu, J. Gong, Nano Energy 2020, 70, 104516.

[281] M.‐F. Lin, V. K. Thakur, E. J. Tan, P. S. Lee, RSC Adv. 2011, 1, 576.

[282] X. Chen, X. Li, J. Shao, N. An, H. Tian, C. Wang, T. Han, L. Wang, B. Lu, Small 2017, 13, 1604245. 10.1002/smll.201604245 28452402

[283] U. Yaqoob, A. S. M. I. Uddin, G.‐S. Chung, Appl. Surf. Sci. 2017, 405, 420.

[284] A. P. Indolia, M. S. Gaur, J. Therm. Anal. Calorim. 2013, 113, 821.

[285] S. Jana, S. Garain, S. K. Ghosh, S. Sen, D. Mandal, Nanotechnology 2016, 27, 445403. 27680679 10.1088/0957-4484/27/44/445403

[286] R. Bhunia, S. Das, S. Dalui, S. Hussain, R. Paul, R. Bhar, A. K. Pal, Appl. Phys. A 2016, 122, 637.

[287] P. Paufler, Cryst. Res. Technol. 1988, 23, 1360.

[288] M. S. S. Bafqi, R. Bagherzadeh, M. Latifi, J. Polym. Res. 2015, 22, 130.

[289] J. Han, D. Li, C. Zhao, X. Wang, J. Li, X. Wu, Sensors 2019, 19, 830.

[290] V. S. Nguyen, D. Rouxel, B. Vincent, L. Badie, F. D. D. Santos, E. Lamouroux, Y. Fort, Appl. Surf. Sci. 2013, 279, 204.

[291] R. Hadji, V. S. Nguyen, B. Vincent, D. Rouxel, F. Bauer, IEEE Trans. Ultrason., Ferroelectr., Freq. Control 2012, 59, 163. 22293747 10.1109/TUFFC.2012.2168

[292] V. S. Nguyen, L. Badie, E. Lamouroux, B. Vincent, F. D. D. Santos, M. Aufray, Y. Fort, D. Rouxel, J. Appl. Polym. Sci. 2013, 129, 391.

[293] P. Thakur, A. Kool, N. A. Hoque, B. Bagchi, F. Khatun, P. Biswas, D. Brahma, S. Roy, S. Banerjee, S. Das, Nano Energy 2018, 44, 456.

[294] H. H. Singh, S. Singh, N. Khare, Polym. Adv. Technol. 2018, 29, 143.

[295] M. Choi, G. Murillo, S. Hwang, J. W. Kim, J. H. Jung, C.‐Y. Chen, M. Lee, Nano Energy 2017, 33, 462.

[296] H. Parangusan, D. Ponnamma, M. A. A. Al‐Maadeed, Sci. Rep. 2018, 8, 754. 29335498 10.1038/s41598-017-19082-3 PMC5768784

[297] H. Parangusan, D. Ponnamma, M. A. A. AlMaadeed, RSC Adv. 2017, 7, 50156.

[298] H. Parangusan, D. Ponnamma, M. A. A. AlMaadeed, Soft Matter 2018, 14, 8803. 30345447 10.1039/c8sm01655k

[299] D. Ponnamma, H. Parangusan, A. Tanvir, M. A. A. AlMa'adeed, Mater. Des. 2019, 184, 108176.

[300] H. Fashandi, M. M. Abolhasani, P. Sandoghdar, N. Zohdi, Q. Li, M. Naebe, Cellulose 2016, 23, 3625.

[301] S. K. Si, S. K. Karan, S. Paria, A. Maitra, A. K. Das, R. Bera, A. Bera, L. Halder, B. B. Khatua, Mater. Chem. Phys. 2018, 213, 525.

[302] I. Chinya, S. Sen, Mater. Today: Proc. 2018, 5, 10047.

[303] I. Chinya, A. Pal, S. Sen, J. Alloys Compd. 2017, 722, 829.

[304] I. Chinya, A. Sasmal, A. Pal, S. Sen, CrystEngComm 2019, 21, 3478.

[305] M. Mishra, A. Roy, S. Dash, S. Mukherjee, IOP Conf. Ser.: Mater. Sci. Eng. 2018, 338, 012026.

[306] V. Tiwari, G. Srivastava, Ceram. Int. 2015, 41, 8008.

[307] Y. J. Choi, M.‐J. Yoo, H.‐W. Kang, H.‐G. Lee, S. H. Han, S. Nahm, J. Electroceram. 2013, 30, 30.

[308] C. K. Wong, F. G. Shin, J. Appl. Phys. 2005, 97, 064111.

[309] Y. H. Son, S. Y. Kweon, S. J. Kim, Y. M. Kim, T. W. Hong, Y. G. Lee, Integr. Ferroelectr. 2007, 88, 44.

[310] C.‐W. Nan, G. J. Weng, J. Appl. Phys. 2000, 88, 416.

[311] P. Han, S. Pang, J. Fan, X. Shen, T. Pan, Sens. Actuators, A 2013, 204, 74.

[312] S. Bairagi, S. W. Ali, Eur. Polym. J. 2019, 116, 554.

[313] S. A. Haddadi, S. A. A. Ramazani, S. Talebi, S. Fattahpour, M. Hasany, Ind. Eng. Chem. Res. 2017, 56, 12596.

[314] S. Hwang, P. P. Hsu, J. Ind. Eng. Chem. 2013, 19, 1377.

[315] I. A. Rahman, V. Padavettan, J. Nanomater. 2012, 2012, 132424.

[316] F. Zhang, X. Ma, C. Cao, J. Li, Y. Zhu, J. Power Sources 2014, 251, 423.

[317] B. Dutta, N. Bose, E. Kar, S. Das, S. Mukherjee, J. Polym. Res. 2017, 24, 220.

[318] B. Dutta, E. Kar, N. Bose, S. Mukherjee, ACS Sustainable Chem. Eng. 2018, 6, 10505.

[319] D. Singh, A. Choudhary, A. Garg, ACS Appl. Mater. Interfaces 2018, 10, 2793. 29278484 10.1021/acsami.7b16973

[320] P. Martins, C. Caparros, R. Gonçalves, P. M. Martins, M. Benelmekki, G. Botelho, S. Lanceros‐Mendez, J. Phys. Chem. C 2012, 116, 15790.

[321] Z.‐W. Ouyang, E.‐C. Chen, T.‐M. Wu, Mater. Chem. Phys. 2015, 149–150, 172.

[322] M. E. Mackay, A. Tuteja, P. M. Duxbury, C. J. Hawker, B. V. Horn, Z. Guan, G. Chen, R. S. Krishnan, Science 2006, 311, 1740. 16556836 10.1126/science.1122225

[323] H. Parangusan, D. Ponnamma, M. A. A. AlMaadeed, ACS Omega 2019, 4, 6312. 31459771 10.1021/acsomega.9b00243 PMC6648750

[324] J. Yang, J. Wang, Q. Zhang, F. Chen, H. Deng, K. Wang, Q. Fu, Polymer 2011, 52, 4970.

[325] K. P. Pramoda, A. Mohamed, I. Y. Phang, T. Liu, Polym. Int. 2005, 54, 226.

[326] S. Ramasundaram, S. Yoon, K. J. Kim, C. Park, J. Polym. Sci., Part B: Polym. Phys. 2008, 46, 2173.

[327] R. Neppalli, S. Wanjale, M. Birajdar, V. Causin, Eur. Polym. J. 2013, 49, 90.

[328] Y. Ma, W. Tong, W. Wang, Q. An, Y. Zhang, Compos. Sci. Technol. 2018, 168, 397.

[329] N. Jahan, F. Mighri, D. Rodrigue, A. Ajji, Appl. Clay Sci. 2018, 152, 93.

[330] W. Rahman, S. K. Ghosh, T. R. Middya, D. Mandal, Mater. Res. Express 2017, 4, 095305.

[331] S. Tiwari, A. Gaur, C. Kumar, P. Maiti, Energy 2019, 171, 485. 10.1039/c9na00214f PMC9418055 36133603

[332] M. Khalifa, A. Mahendran, S. Anandhan, RSC Adv. 2016, 6, 114052.

[333] S. Shetty, A. Mahendran, S. Anandhan, Soft Matter 2020, 16, 5679. 32519712 10.1039/d0sm00341g

[334] S. Harstad, N. D'Souza, N. Soin, A. A. El‐Gendy, S. Gupta, V. K. Pecharsky, T. Shah, E. Siores, R. L. Hadimani, AIP Adv. 2017, 7, 056411.

[335] K. Maity, B. Mahanty, T. K. Sinha, S. Garain, A. Biswas, S. K. Ghosh, S. Manna, S. K. Ray, D. Mandal, Energy Technol. 2017, 5, 234.

[336] H. Paik, Y.‐Y. Choi, S. Hong, K. No, Sci. Rep. 2015, 5, 13209. 26336795 10.1038/srep13209 PMC4559747

[337] Y. Ahn, J. Y. Lim, S. M. Hong, J. Lee, J. Ha, H. J. Choi, Y. Seo, J. Phys. Chem. C 2013, 117, 11791.

[338] Alamusi, J. Xue, L. Wu, N. Hu, J. Qiu, C. Chang, S. Atobe, H. Fukunaga, T. Watanabe, Y. Liu, H. Ning, J. Li, Y. Li, Y. Zhao, Nanoscale 2012, 4, 7250. 23281491 10.1039/c2nr32185h

[339] H.‐J. Chen, S. Han, C. Liu, Z. Luo, H.‐P. D. Shieh, R.‐S. Hsiao, B.‐R. Yang, Sens. Actuators, A 2016, 245, 135.

[340] A. A. Issa, M. A. Al‐Maadeed, A. S. Luyt, D. Ponnamma, M. K. Hassan, C 2017, 3, 30.

[341] B. Li, C. Xu, J. Zheng, C. Xu, Sensors 2014, 14, 9889. 24901980 10.3390/s140609889 PMC4118383

[342] S. K. Ghosh, D. Mandal, Nano Energy 2018, 53, 245.

[343] E. Kar, N. Bose, B. Dutta, S. Banerjee, N. Mukherjee, S. Mukherjee, Energy Convers. Manage. 2019, 184, 600.

[344] S. Yu, W. Zheng, W. Yu, Y. Zhang, Q. Jiang, Z. Zhao, Macromolecules 2009, 42, 8870.

[345] Z. H. Liu, C. T. Pan, L. W. Lin, H. W. Lai, Sens. Actuators, A 2013, 193, 13.

[346] M. C. Celina, T. R. Dargaville, P. M. Chaplya, R. L. Clough, MRS Online Proc. Libr. 2004, 851, 499.

[347] H. Yu, T. Huang, M. Lu, M. Mao, Q. Zhang, H. Wang, Nanotechnology 2013, 24, 405401. 24029779 10.1088/0957-4484/24/40/405401

[348] C.‐M. Wu, M.‐H. Chou, W.‐Y. Zeng, Nanomaterials 2018, 8, 420.

[349] A. Mandal, A. K. Nandi, ACS Appl. Mater. Interfaces 2013, 5, 747. 23281687 10.1021/am302275b

[350] K. Ke, P. Pötschke, D. Jehnichen, D. Fischer, B. Voit, Polymer 2014, 55, 611.

[351] M. Sharma, V. Srinivas, G. Madras, S. Bose, RSC Adv. 2016, 6, 6251.

[352] L. He, G. Xia, J. Sun, Q. Zhao, R. Song, Z. Ma, J. Colloid Interface Sci. 2013, 393, 97. 23245886 10.1016/j.jcis.2012.10.060

[353] D. V. Kosynkin, A. L. Higginbotham, A. Sinitskii, J. R. Lomeda, A. Dimiev, B. K. Price, J. M. Tour, Nature 2009, 458, 872. 19370030 10.1038/nature07872

[354] L. Yang, H. Ji, K. Zhu, J. Wang, J. Qiu, Compos. Sci. Technol. 2016, 123, 259.

[355] C. Baur, J. R. DiMaio, E. McAllister, R. Hossini, E. Wagener, J. Ballato, S. Priya, A. Ballato, D. W. Smith, J. Appl. Phys. 2012, 112, 124104.

[356] H. W. Kroto, J. R. Heath, S. C. O'Brien, R. F. Curl, R. E. Smalley, Nature 1985, 318, 162.

[357] L. Wu, M. Jing, Y. Liu, H. Ning, X. Liu, S. Liu, L. Lin, N. Hu, L. Liu, Composites, Part B 2019, 164, 703.

[358] M. M. Abolhasani, K. Shirvanimoghaddam, M. Naebe, Compos. Sci. Technol. 2017, 138, 49.

[359] H. C. Bidsorkhi, A. G. D'Aloia, G. De Bellis, A. Proietti, A. Rinaldi, M. Fortunato, P. Ballirano, M. P. Bracciale, M. L. Santarelli, M. S. Sarto, Mater. Today Commun. 2017, 11, 163.

[360] Y. Zhang, S. Jiang, M. Fan, Y. Zeng, Y. Yu, J. He, J. Mater. Sci.: Mater. Electron. 2013, 24, 927.

[361] S. Garain, S. Jana, T. K. Sinha, D. Mandal, ACS Appl. Mater. Interfaces 2016, 8, 4532. 26829464 10.1021/acsami.5b11356

[362] T. K. Sinha, S. K. Ghosh, R. Maiti, S. Jana, B. Adhikari, D. Mandal, S. K. Ray, ACS Appl. Mater. Interfaces 2016, 8, 14986. 27266368 10.1021/acsami.6b01547

[363] R. Barstugan, M. Barstugan, I. Ozaytekin, Composites, Part B 2019, 158, 141.

[364] M. A. Rahman, B.‐C. Lee, D.‐T. Phan, G.‐S. Chung, Smart Mater. Struct. 2013, 22, 085017.

[365] M. A. Rahman, G.‐S. Chung, J. Alloys Compd. 2013, 581, 724.

[366] C. Kumar, A. Gaur, S. K. Rai, P. Maiti, Nano‐Struct. Nano‐Objects 2017, 12, 174.

[367] R. M. Habibur, U. Yaqoob, S. Muhammad, A. S. M. I. Uddin, H. C. Kim, Mater. Chem. Phys. 2018, 215, 46.

[368] S. K. Karan, D. Mandal, B. B. Khatua, Nanoscale 2015, 7, 10655. 26030744 10.1039/c5nr02067k

[369] M. Pusty, L. Sinha, P. M. Shirage, New J. Chem. 2019, 43, 284.

[370] B. Jaleh, A. Jabbari, Appl. Surf. Sci. 2014, 320, 339.

[371] S. K. Karan, R. Bera, S. Paria, A. K. Das, S. Maiti, A. Maitra, B. B. Khatua, Adv. Energy Mater. 2016, 6, 1601016.

[372] K. Roy, D. Mandal, AIP Conf. Proc. 2018, 1942, 050125.

[373] M. Pusty, A. Sharma, L. Sinha, A. Chaudhary, P. Shirage, ChemistrySelect 2017, 2, 2774.

[374] M. El Achaby, F. Z. Arrakhiz, S. Vaudreuil, E. M. Essassi, A. Qaiss, Appl. Surf. Sci. 2012, 258, 7668.

[375] W. S. Hummers, R. E. Offeman, J. American Chem. Socie. 1958, 80, 1339.

[376] S. Singh, N. Khare, RSC Adv. 2015, 5, 96562.

[377] Z. Y. Jiang, G. P. Zheng, K. Zhan, Z. Han, J. H. Yang, J. Phys. D: Appl. Phys. 2015, 48, 245303.

[378] A. Gebrekrstos, G. Madras, S. Bose, ACS Omega 2018, 3, 5317. 31458741 10.1021/acsomega.8b00237 PMC6641698

[379] H. Kim, F. Torres, M. T. Islam, M. D. Islam, L. A. Chavez, C. A. G. Rosales, B. R. Wilburn, C. M. Stewart, J. C. Noveron, T.‐L. B. Tseng, Y. Lin, MRS Commun. 2017, 7, 960.

[380] D. Ponnamma, M. A. A. Al‐Maadeed, Sustainable Energy Fuels 2019, 3, 774.

[381] E. A. Bakar, M. A. Mohamed, P. C. Ooi, M. F. M. R. Wee, C. F. Dee, B. Y. Majlis, Org. Electron. 2018, 61, 289.

[382] A. Al‐Saygh, D. Ponnamma, M. AlMaadeed, P. P. Vijayan, A. Karim, M. Hassan, Polymers 2017, 9, 33. 10.3390/polym9020033 PMC6432156 30970716

[383] D. Ponnamma, A. K. Sharma, P. Saharan, M. A. A. Al‐Maadeed, J. Electron. Mater. 2020, 49, 2677.

[384] D. Ponnamma, P. Vijayan P, M. A. A. Al‐Maadeed, Mater. Des. 2017, 117, 203.

[385] A. Samadi, S. M. Hosseini, M. Mohseni, Org. Electron. 2018, 59, 149.

[386] A. Samadi, R. Ahmadi, S. M. Hosseini, Org. Electron. 2019, 75, 105405.

[387] M. Abbasipour, R. Khajavi, A. A. Yousefi, M. E. Yazdanshenas, F. Razaghian, J. Mater. Sci.: Mater. Electron. 2017, 28, 15942.

[388] E. B. Araújo, in Advances in Ceramics – Electric and Magnetic Ceramics, Bioceramics, Ceramics and Environment, (Ed: C. Sikalidis), IntechOpen, London 2011, Ch. 3.

[389] S. Das, A. K. Biswal, K. Parida, R. N. P. Choudhary, A. Roy, Appl. Surf. Sci. 2018, 428, 356.

[390] S. Bodkhe, P. S. M. Rajesh, S. Kamle, V. Verma, J. Polym. Res. 2014, 21, 434.

[391] J. S. Andrew, D. R. Clarke, Langmuir 2008, 24, 670. 18189433 10.1021/la7035407

[392] K.‐I. Park, S. B. Bae, S. H. Yang, H. I. Lee, K. Lee, S. J. Lee, Nanoscale 2014, 6, 8962. 24967905 10.1039/c4nr02246g

[393] C. Baek, J. H. Yun, H. S. Wang, J. E. Wang, H. Park, K.‐I. Park, D. K. Kim, Appl. Surf. Sci. 2018, 429, 164.

[394] Z.‐H. Lin, Y. Yang, J. M. Wu, Y. Liu, F. Zhang, Z. L. Wang, J. Phys. Chem. Lett. 2012, 3, 3599. 26290994 10.1021/jz301805f

[395] J. Yan, Y. G. Jeong, ACS Appl. Mater. Interfaces 2016, 8, 15700. 27237223 10.1021/acsami.6b02177

[396] N. R. Alluri, A. Chandrasekhar, V. Vivekananthan, Y. Purusothaman, S. Selvarajan, J. H. Jeong, S.‐J. Kim, ACS Sustainable Chem. Eng. 2017, 5, 4730.

[397] C. K. Jeong, I. Kim, K.‐I. Park, M. H. Oh, H. Paik, G.‐T. Hwang, K. No, Y. S. Nam, K. J. Lee, ACS Nano 2013, 7, 11016. 24229091 10.1021/nn404659d

[398] K. Y. Lee, D. Kim, J.‐H. Lee, T. Y. Kim, M. K. Gupta, S.‐W. Kim, Adv. Funct. Mater. 2014, 24, 37.

[399] S.‐H. Shin, Y.‐H. Kim, M. H. Lee, J.‐Y. Jung, J. H. Seol, J. Nah, ACS Nano 2014, 8, 10844. 25265473 10.1021/nn5046568

[400] X. S. Wang, Z. C. Wu, J. F. Webb, Z. G. Liu, Appl. Phys. A 2003, 77, 561.

[401] J. H. Jung, M. Lee, J.‐I. Hong, Y. Ding, C.‐Y. Chen, L.‐J. Chou, Z. L. Wang, ACS Nano 2011, 5, 10041. 22098313 10.1021/nn2039033

[402] J. Chun, N.‐R. Kang, J.‐Y. Kim, M.‐S. Noh, C.‐Y. Kang, D. Choi, S.‐W. Kim, Z. L. Wang, J. M. Baik, Nano Energy 2015, 11, 1.

[403] S. Xu, Y. Yeh, G. Poirier, M. C. McAlpine, R. A. Register, N. Yao, Nano Lett. 2013, 13, 2393. 23634729 10.1021/nl400169t

[404] N. Abinnas, P. Baskaran, S. Harish, R. S. Ganesh, M. Navaneethan, K. D. Nisha, S. Ponnusamy, C. Muthamizhchelvan, H. Ikeda, Y. Hayakawa, Appl. Surf. Sci. 2017, 418, 362.

[405] Q. Zheng, H. Zhang, H. Mi, Z. Cai, Z. Ma, S. Gong, Nano Energy 2016, 26, 504.

[406] T. Zhai, Q. Zheng, Z. Cai, L.‐S. Turng, H. Xia, S. Gong, ACS Appl. Mater. Interfaces 2015, 7, 7436. 25822398 10.1021/acsami.5b01679

[407] R. Ding, H. Liu, X. Zhang, J. Xiao, R. Kishor, H. Sun, B. Zhu, G. Chen, F. Gao, X. Feng, J. Chen, X. Chen, X. Sun, Y. Zheng, Adv. Funct. Mater. 2016, 26, 7708.

[408] R. Ding, X. Zhang, G. Chen, H. Wang, R. Kishor, J. Xiao, F. Gao, K. Zeng, X. Chen, X. W. Sun, Y. Zheng, Nano Energy 2017, 37, 126.

[409] Z. Deng, Y. Dai, W. Chen, X. Pei, J. Liao, Nanoscale Res. Lett. 2010, 5, 1217. 20596350 10.1007/s11671-010-9629-7 PMC2894195

[410] T.‐Y. Ke, H.‐A. Chen, H.‐S. Sheu, J.‐W. Yeh, H.‐N. Lin, C.‐Y. Lee, H.‐T. Chiu, J. Phys. Chem. C 2008, 112, 8827.

[411] S. Hajra, M. Sahu, D. Oh, H. J. Kim, Ceram. Int. 2021, 47, 15695.

[412] J. Yan, Y. G. Jeong, Compos. Sci. Technol. 2017, 144, 1.

[413] C. Luo, S. Hu, M. Xia, P. Li, J. Hu, G. Li, H. Jiang, W. Zhang, Energy Technol. 2018, 6, 922.

[414] H. Sun, H. Tian, Y. Yang, D. Xie, Y.‐C. Zhang, X. Liu, S. Ma, H.‐M. Zhao, T.‐L. Ren, Nanoscale 2013, 5, 6117. 23728508 10.1039/c3nr00866e

[415] K.‐I. Park, C. K. Jeong, J. Ryu, G.‐T. Hwang, K. J. Lee, Adv. Energy Mater. 2013, 3, 1539.

[416] C. K. Jeong, J. Lee, S. Han, J. Ryu, G.‐T. Hwang, D. Y. Park, J. H. Park, S. S. Lee, M. Byun, S. H. Ko, K. J. Lee, Adv. Mater. 2015, 27, 2866. 25824939 10.1002/adma.201500367

[417] P. Lee, J. Lee, H. Lee, J. Yeo, S. Hong, K. H. Nam, D. Lee, S. S. Lee, S. H. Ko, Adv. Mater. 2012, 24, 3326. 22610599 10.1002/adma.201200359

[418] C. K. Jeong, K.‐I. Park, J. Ryu, G.‐T. Hwang, K. J. Lee, Adv. Funct. Mater. 2014, 24, 2620.

[419] M. M. Alam, D. Mandal, ACS Appl. Mater. Interfaces 2016, 8, 1555. 26760435 10.1021/acsami.5b08168

[420] N. A. Hoque, P. Thakur, S. Roy, A. Kool, B. Bagchi, P. Biswas, M. M. Saikh, F. Khatun, S. Das, P. P. Ray, ACS Appl. Mater. Interfaces 2017, 9, 23048. 28613807 10.1021/acsami.7b08008

[421] M. Fortunato, C. R. Chandraiahgari, G. De Bellis, P. Ballirano, F. Sarto, A. Tamburrano, M. S. Sarto, Nanomaterials 2018, 8, 743. 10.3390/nano8090743 PMC6165421 30235819

[422] S. Jana, S. Garain, S. Sen, D. Mandal, Phys. Chem. Chem. Phys. 2015, 17, 17429. 26077827 10.1039/c5cp01820j

[423] D. Dhakras, V. Borkar, S. Ogale, J. Jog, Nanoscale 2012, 4, 752. 22234432 10.1039/c2nr11841f

[424] F. Mokhtari, M. Shamshirsaz, M. Latifi, J. Foroughi, Polymers 2020, 12, 2697. 10.3390/polym12112697 PMC7696415 33207703

[425] B. Yu, M. Mao, H. Yu, T. Huang, W. Zuo, H. Wang, M. Zhu, Macromol. Mater. Eng. 2017, 302, 1700214.

[426] C. Chen, Z. Bai, Y. Cao, M. Dong, K. Jiang, Y. Zhou, Y. Tao, S. Gu, J. Xu, X. Yin, W. Xu, Compos. Sci. Technol. 2020, 192, 108100.

[427] P. Thakur, A. Kool, B. Bagchi, N. A. Hoque, S. Das, P. Nandy, RSC Adv. 2015, 5, 28487.

[428] K.‐I. Park, J. H. Son, G.‐T. Hwang, C. K. Jeong, J. Ryu, M. Koo, I. Choi, S. H. Lee, M. Byun, Z. L. Wang, K. J. Lee, Adv. Mater. 2014, 26, 2450. 10.1002/adma.201305659 24523251

[429] V. Bhavanasi, V. Kumar, K. Parida, J. Wang, P. S. Lee, ACS Appl. Mater. Interfaces 2016, 8, 521. 26693844 10.1021/acsami.5b09502

[430] K.‐I. Park, S. Xu, Y. Liu, G.‐T. Hwang, S.‐J. L. Kang, Z. L. Wang, K. J. Lee, Nano Lett. 2010, 10, 4939. 21050010 10.1021/nl102959k

[431] J. Lim, H. Jung, C. Baek, G.‐T. Hwang, J. Ryu, D. Yoon, J. Yoo, K.‐I. Park, J. H. Kim, Nano Energy 2017, 41, 337.

[432] K. Y. Lee, B. Kumar, J.‐S. Seo, K.‐H. Kim, J. I. Sohn, S. N. Cha, D. Choi, Z. L. Wang, S.‐W. Kim, Nano Lett. 2012, 12, 1959. 22409420 10.1021/nl204440g

[433] K. Shi, B. Sun, X. Huang, P. Jiang, Nano Energy 2018, 52, 153.

[434] N. R. Alluri, B. Saravanakumar, S.‐J. Kim, ACS Appl. Mater. Interfaces 2015, 7, 9831. 25901640 10.1021/acsami.5b01760

[435] B. Saravanakumar, S. Soyoon, S.‐J. Kim, ACS Appl. Mater. Interfaces 2014, 6, 13716. 25068976 10.1021/am5031648

[436] S. Lee, R. Hinchet, Y. Lee, Y. Yang, Z.‐H. Lin, G. Ardila, L. Montès, M. Mouis, Z. L. Wang, Adv. Funct. Mater. 2014, 24, 1163.

[437] S. Lee, S.‐H. Bae, L. Lin, Y. Yang, C. Park, S.‐W. Kim, S. N. Cha, H. Kim, Y. J. Park, Z. L. Wang, Adv. Funct. Mater. 2013, 23, 2445.

[438] W. Guo, C. Tan, K. Shi, J. Li, X.‐X. Wang, B. Sun, X. Huang, Y.‐Z. Long, P. Jiang, Nanoscale 2018, 10, 17751. 30211423 10.1039/c8nr05292a

[439] B. Dudem, D. H. Kim, L. K. Bharat, J. S. Yu, Appl. Energy 2018, 230, 865.

[440] M. Lee, J. Bae, J. Lee, C.‐S. Lee, S. Hong, Z. L. Wang, Energy Environ. Sci. 2011, 4, 3359.

[441] Y. Hu, C. Xu, Y. Zhang, L. Lin, R. L. Snyder, Z. L. Wang, Adv. Mater. 2011, 23, 4068. 21812038 10.1002/adma.201102067

[442] L. Lin, Y. Hu, C. Xu, Y. Zhang, R. Zhang, X. Wen, Z. L. Wang, Nano Energy 2013, 2, 75.

[443] R. Zhang, L. Lin, Q. Jing, W. Wu, Y. Zhang, Z. Jiao, L. Yan, R. P. S. Han, Z. L. Wang, Energy Environ. Sci. 2012, 5, 8528.

